# Comprehensive comparative analysis of kinesins in photosynthetic eukaryotes

**DOI:** 10.1186/1471-2164-7-18

**Published:** 2006-01-31

**Authors:** Dale N Richardson, Mark P Simmons, Anireddy SN Reddy

**Affiliations:** 1 Department of Biology, Colorado State University, Fort Collins, CO, USA, 80523; 2 Department of Biology and Program in Cell and Molecular Biology, Colorado State University, Fort Collins, CO, USA, 80523

## Abstract

**Background:**

Kinesins, a superfamily of molecular motors, use microtubules as tracks and transport diverse cellular cargoes. All kinesins contain a highly conserved ~350 amino acid motor domain. Previous analysis of the completed genome sequence of one flowering plant (Arabidopsis) has resulted in identification of 61 kinesins. The recent completion of genome sequencing of several photosynthetic and non-photosynthetic eukaryotes that belong to divergent lineages offers a unique opportunity to conduct a comprehensive comparative analysis of kinesins in plant and non-plant systems and infer their evolutionary relationships.

**Results:**

We used the kinesin motor domain to identify kinesins in the completed genome sequences of 19 species, including 13 newly sequenced genomes. Among the newly analyzed genomes, six represent photosynthetic eukaryotes. A total of 529 kinesins was used to perform comprehensive analysis of kinesins and to construct gene trees using the Bayesian and parsimony approaches. The previously recognized 14 families of kinesins are resolved as distinct lineages in our inferred gene tree. At least three of the 14 kinesin families are not represented in flowering plants. *Chlamydomonas*, a green alga that is part of the lineage that includes land plants, has at least nine of the 14 known kinesin families. Seven of ten families present in flowering plants are represented in *Chlamydomonas*, indicating that these families were retained in both the flowering-plant and green algae lineages.

**Conclusion:**

The increase in the number of kinesins in flowering plants is due to vast expansion of the Kinesin-14 and Kinesin-7 families. The Kinesin-14 family, which typically contains a C-terminal motor, has many plant kinesins that have the motor domain at the N terminus, in the middle, or the C terminus. Several domains in kinesins are present exclusively either in plant or animal lineages. Addition of novel domains to kinesins in lineage-specific groups contributed to the functional diversification of kinesins. Results from our gene-tree analyses indicate that there was tremendous lineage-specific duplication and diversification of kinesins in eukaryotes. Since the functions of only a few plant kinesins are reported in the literature, this comprehensive comparative analysis will be useful in designing functional studies with photosynthetic eukaryotes.

## Background

Cytoskeletal networks (microtubules [MTs], actin and intermediate filaments) play important roles in many fundamental processes in eukaryotes including cell growth, cell division and development of organisms [1,2]. Understanding cytoskeleton organization, dynamics and functions is an active area of research in biology. Molecular motors that organize and remodel cytoskeleton and transport various cellular components (e.g, vesicles, organelles, chromosomes, RNA and protein complexes) play fundamental roles in all aspects of cell and developmental biology of eukaryotes [1,2]. High throughput genomic sequencing projects have greatly facilitated the identification of the full complement of molecular motors in several phylogenetically diverse species ranging from simple unicellular to complex multicellular organisms [1,2].

Molecular motors that function on cytoskeletal networks belong to three groups: kinesins, dyneins and myosins. These motors utilize energy derived from ATP hydrolysis and transport cargo unidirectionally on one of the filamentous cytoskeletal tracks (MTs or F-actin) in the cell. Both kinesins and dyneins use MTs as tracks for motility whereas myosins use actin filaments (F-actin) [3,4]. Kinesins constitute a superfamily of MT motor proteins ubiquitous in all eukaryotic organisms [1,2,5,6]. In the mid 1980s, the first kinesin was discovered in squid giant axons as a "novel force generating protein" involved in vesicular transport [7]. Since then, an explosion of research has centered upon the continual discovery, classification and functional characterization of the kinesin superfamily. Members of the kinesin superfamily have a highly conserved motor domain of ~350 amino acid residues, which contains ATPase and MT binding activities, located at the N terminus, C terminus or internally [1,8]. A short neck region that often contains family-specific features and is adjacent to the motor domain works in concert with the catalytic core to produce movement [8,9]. In addition, many kinesins have a less conserved coiled-coil region that is important for dimerization and a non-conserved tail domain that is thought to interact with specific cargo. All kinesins bind MTs and perform a variety of force-generating tasks such as movement of chromosomes, vesicles, organelles and RNA protein complexes, spindle formation and elongation, activation of protein kinases, movement of loosely bound rafts of soluble cargo, and MT polymerization and dynamics [5,9-13].

Since the motor-domain sequence is conserved in all kinesins, it has been used to search completed eukaryotic genome sequences for encoded kinesins. Based on phylogenetic analyses of known kinesins using the conserved motor domain sequences, fourteen families designated as Kinesin-1 to Kinesin-14 are recognized [14]. Members of most families have an N-terminal motor domain whereas one family (Kin-13) has an internal motor and one family (Kin-14) has a C-terminal motor. Kinesins move unidirectionally on MTs. Kinesins with the N-terminal motor show plus end motility whereas the C-terminal motors move toward the minus ends of MTs [1,15-17].

While a "complete" inventory of Arabidopsis kinesins has been reported, functional studies of plant kinesins are limited to a few loci [18,19]. Several plant kinesins have been shown to function in mitosis, meiosis and/or cytokinesis [20-28]. KCBP, a C-terminal minus-end-directed calmodulin-binding kinesin of the Kinesin-14 family, is involved in trichome morphogenesis and cell division [29-31]. This kinesin is negatively regulated by calmodulin as well as another novel calcium-binding protein (KIC) with a single EF hand [32,33]. An internal kinesin of the Kinesin-13 family in Arabidopsis is also involved in trichome morphogenesis [34]. AtFRAl, an N-terminal kinesin family member is involved in oriented deposition of cellulose myofibrils; mutants show aberrant deposition of cellulose microfibrils in secondary walls of fibers that are less organized when compared to the wild type [35]. Two Arabidopsis kinesins are targeted to mitochondria whereas another kinesin interacts with geminivirus replicating protein [36,37]. An interesting prospect of MT and microfilament crosstalk has recently been exemplified by studies with a plant-specific kinesin (GhKCHl) from cotton. This member of the Kinesin-14 family has a calponin homology domain, which appears to be important in mediating dynamic interaction between actin filaments and MTs [38]. The motility properties of only a few plant kinesins have been analyzed [39-41].

Thus far, genome-wide analysis of kinesin encoding genes in plants was performed only with one plant species (*Arabidopsis thaliand*), which uncovered 61 kinesins. The number of kinesins in Arabidopsis is the largest as compared to human, mouse and other completed genomes [42,43]. Recently, genome sequences of six phylogenetically divergent photosynthetic eukaryotes (two cultivars of rice, poplar, a green alga, a red alga and a diatom) have been completed. In addition, genomes of seven other non-plant systems including *Giardia*, which may represent the deepest known branch in the eukaryotic lineage ([44-47]; but see [48,49]) have also been sequenced. The availability of these genome sequences offers opportunities to address a number of important questions related to kinesin evolution and function. These include: i) do other plants, like Arabidopsis, have a large repertoire of kinesins? ii) are there any kinesin families that are specific to plants or a particular lineage? iii) how many kinesin families are represented in all eukaryotes? iv) what is the evolutionary relationship among plant kinesins and between plant and non-plant kinesins? v) what is the full complement of kinesins in early-derived simple unicellular photosynthetic eukaryotes as well as in organisms that represent early diverging eukaryotic lineages? vi) how have these kinesins contributed to evolution of kinesins in the most recent complex multicellular flowering plants? vii) do plant kinesins have any domains that are unique to plants? and viii) what is the contribution of gene duplications and losses to kinesin evolution? To address these questions, we have mined 529 kinesin sequences from 19 phylogenetically diverse species. We have performed comprehensive analyses with this data set and inferred gene trees using Bayesian and parsimony methods. Our gene-tree analyses included 249 sequences from photosynthetic eukaryotes and 280 from non-photosynthetic systems. Many of these sequences were not included in any previous analyses. Although flowering plants have the largest number of kinesins, three or four of the 14 kinesin families are not represented in flowering plants whereas three of them may not be present in any photosynthetic eukaryote. Results presented here also indicate that flowering plants have the most kinesins primarily due to expansion of the Kinesin-7 and Kinesin-14 families. Our gene-tree analysis revealed seven of the ten families found in flowering plants are represented in a simple unicellular chlorophyte alga. Ten of the 14 families are represented in *Giardia lamblia *([44-47]; but see [48,49]), an early derived eukaryote, suggesting that most families were already present early in the evolution of extant eukaryotes. Plant kinesins have several domains that are not shared with non-plant systems suggesting functional specificity and diversification in plants.

## Results and Discussion

### Kinesins in photosynthetic and non-photosynthetic eukaryotes

In this study we have analyzed genome sequences of 19 eukaryotic species, which represent almost all major lineages (opisthokonts, amoebozoa, plants, alveolates, heterokonts, discicristates and excavates) of eukaryotes [50], for kinesins. Species were selected so as to include most of the eight major lineages in the consensus phylogenetic tree presented by Baldauf [50]. Inclusion of representative members of non-plant groups is expected to help us identify plant-specific kinesins. Of the eight major eukaryotic lineages [50], only one lineage (cercozoa) was not sampled in our analysis because none of the species in this lineage has been fully sequenced. Among the 19 species analyzed, seven represent phylogenetically divergent photosynthetic eukaryotes that belong to monocots (two rice cultivars, *Oryza sativa *ssp. *japonica *cv. *nipponbare *and *Oryza sativa *ssp. *indica *cv *93-11*), dicots (*Arabidopsis thaliana *and *Populus trichocarpa*), a chlorophyte alga (*Chlamydomonas reinhardtii*), a red alga (*Cyanidioschyzon merolae*) and a diatom (*Thalassiosira pseudonana*). So far, kinesins have been analyzed only in one plant (Arabidopsis) system [43] whereas the genome sequences of six other photosynthetic eukaryotes have been completed recently. The red (*C. merolae*) and green (*C. reinhardtii*) algae were included in our analysis because they are inferred to be early derived members of the lineage that gave rise to modern heterokonts and embryophyta [51], respectively. Inclusion of these species allows for the analysis of evolutionary relationships between kinesins of algae and flowering plants. The 12 non-plant species sampled include opisthokonts, amoebozoa, alveolates, heterokonts, discicristates and excavates. We have included members (*Giardia*, *Leishmania*, *Plasmodiuni*) of three extant lineages that diverged before the plant-animal split [46]. In addition, *Giardia *is thought to be a member of the earliest extant branch on the eukaryotic tree based on the phylogeny inferred from several different genes, as well as a proteome-based eukaryotic phylogeny [44-47]. It would be interesting to see how many kinesin families were present before the divergence of plants, animals and fungi as it is likely that these families would represent a "basic set" of kinesin motors [52]. We have also included *Dictyostelium discoideum*, which is believed to have diverged after the plant-animal split but before the divergence of fungi from animals [46].

**Figure 1:**
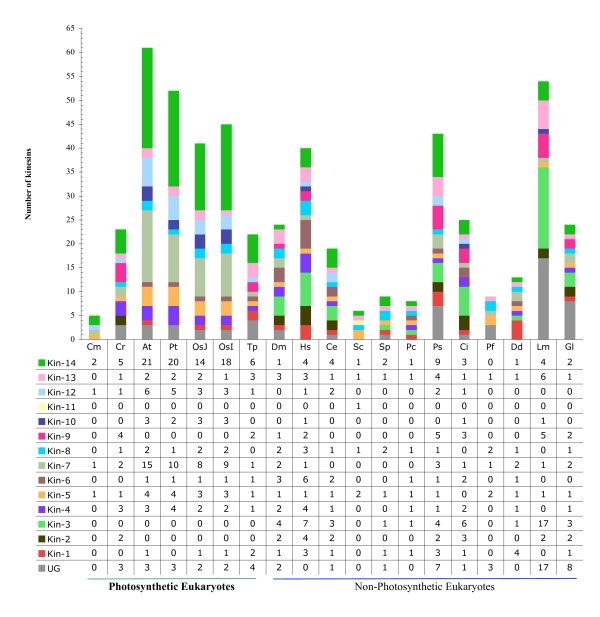
**Number and distribution of kinesins**. Tabular and graphical representation of the number of kinesins found in completely sequenced genomes used in our analysis. The number of kinesins is shown on the y-axis with the 19 species displayed across the x-axis. Different colors represent the distribution of kinesins into specific families. The data table below the chart details the specific number of kinesins in each family per species. For individual sequence IDs, please see Tables 2 through 7 and Additional files 1 through 12. *Cm, Cyanidoschyzon merolae; Cr, Chlamydomonas reinhardtii; At, Arabidopsis thaliana; Pt, Populus trichocarpa; OsJ, Oryza sativa ssp*. *Japonica; OsI, Oryza sativa ssp. Indica; Tp, Thallassiosira pseudonana; Dm, Drosophila melanogaster; Hs, Homo sapiens; Ce, Caenorhabditis elegans; Sc, Saccharomyces cerevisiae; Sp, Schizosaccharomyces pombe; pc, Phaenerochaete chryosporium; Ps, Phytopthora sojae, Ci, Ciona intestinalis; Pf; Plasmodiumfalciparum; Dd, Dictyostelium discoideum; Lm, Leishmania major; Gl, Giardia lamblia*.

As detailed in the methods section, we have used a variety of approaches to systematically analyze the completed genome sequences of 19 species to identify the kinesins. A total of 529 kinesins were identified and used in our phylogenetic analysis. Table 1 shows the number of kinesins in each of these species and lists the databases used in our searches. The details of the kinesins including gene IDs, gene organization, domain and family information for each species, except Arabidopsis, are presented in Tables 2 to 7 and Additional files 1 to 12. The details of Arabidopsis kinesins were reported previously [43]. The number of kinesins varies considerably among species. In general flowering plants have the highest number of kinesins (Fig. 1). Arabidopsis still has the largest repertoire of kinesins [61] amongst the completed plant genomes, with *P*. *trichocarpa *next [52]. *Oryza sativa *ssp. *japonica *and *O.s. indica *have 41 and 45 kinesins, respectively. Some changes in this number may occur as refinement of newly sequenced genomes proceeds. Not only does Arabidopsis have the most kinesins of all plants; it has the most of all 19 species analyzed (Fig. 1). It is surprising to see the large difference in kinesin number between the two species of early-derived unicellular photosynthetic eukaryotes (C. *reinhardtii *and *C. merolae*). The green alga, *C. reinhardtii*, has about five times [23] the number of kinesins as the red algae, *C. merolae*, which has the least number of kinesins (only five) of all species sampled. *Dictyostelium discoideum*, the social soil amoeba, has 13 kinesins. This is consistent with what one would expect from a free living amoeba that must search for its food and be active in cytoskeletal motility, in addition to being able to shift from a unicellular state to a multicellular fruiting body by coordinated aggregation of individual cells [46]. Another interesting species with many kinesins is the intracellular immune system pathogen *L. major*. This parasite has the most kinesins among non-photosynthetic eukaryotes (Fig. 1). *Leishmania *has a considerably larger repertoire of kinesins than the amoeboid parasite, many of which appear to have evolved by multiple gene duplications (see below). Even though *cis-*splicing machinery exists in this parasite, its 54 kinesins are all translated from single exon genes (Chris Peacock, pers, comm.). The reason why this parasite has so many kinesins is an interesting prospect to consider. How many of these kinesins are actually functional remains to be seen. Whether or not these kinesins function in facilitating *Leishmania-*host-cell interaction is currently unknown.

### Construction and analyses of kinesin gene trees

We used 529 kinesin motor domain sequences in our gene-tree analysis. The alignment of amino acid sequences was performed using DIALIGN-T [53]. DIALIGN-T represents an improvement over DIALIGN 2.2.1 [54] in that it is less liable to favor short sequence fragments of high similarity over longer fragments of lower similarity [53]. In contrast to programs such as Clustal X [55], which perform global alignments, DIALIGN finds regions of local similarity without necessarily aligning the entire sequences with one another [56]. Because DIALIGN does not perform alignments following a guide tree, the alignments produced were expected to be relatively robust to artifacts that may be introduced when aligning divergent sequences in a pairwise manner [57]. DIALIGN has been shown to perform well relative to other alignment programs in aligning conserved domains within rapidly evolving (with respect to both indels and substitutions) regions [53,58,59]. Four kinesin trees were constructed with motor domain amino acid sequences using both the parsimony and Bayesian approaches. For each approach, amino-acid-characters and amino-acid-plus-gap-characters were used. The DIALIGN-T alignment (see Additional file 13), data matrices (see Additional files 14 to 17) used to construct the tree and two parsimony and one Bayesian trees (see Additional files 18 to 20) are available online.

**Figure 2:**
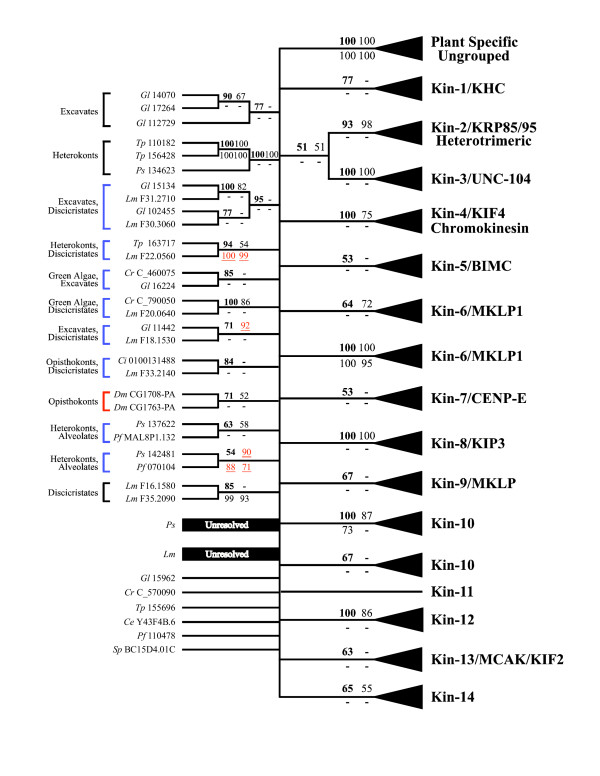
**Unrooted Bayesian tree of the 529 kinesins based on their motor domain ami no-acid-plus-gap-characters**. Support values from each parsimony and Bayesian analysis are presented adjacent to the nodes. Support values above each branch correspond to Bayesian posterior probabilities whereas values below each branch correspond to parsimony jackknife support. In both cases the leftmost values are for the amino-acid-plus-gap-characters analyses and the rightmost values are for amino-acid-characters analyses. Bayesian posterior probabilities for the amino-acid-plus-gap-characters are also shown in bold. If a branch was unresolved in one of the other three analyses, it is indicated by "-" at the respective node. If a branch was contradicted in one of these other three analyses, it is indicated by underlined red font at the respective node with the single highest posterior probability or jackknife support value for the contradicting clade(s) shown. Ungrouped kinesins are presented on the left side of the main polytomy, with the exception of the plant-specific ungrouped family that is shown on the upper right of the tree. The *Ps *and *Lm *unresolved blocks each contain 4 and 9 sequences, respectively (See Additional files 7 and 11 these sequence IDs). Brackets denote the major eukaryotic groupings in accordance with Baldauf's nomenclature [50]. Blue brackets indicate taxa that are from multiple groups, black brackets indicate protozoan species and red brackets are reserved for opisthokonts. Although Pt00151235 is grouped within this family by Bayesian analysis, we favor the parsimony resolution of it as a member of the Kinesin-10 family. For full names of species see Fig. 1 legend.

**Figure 3:**
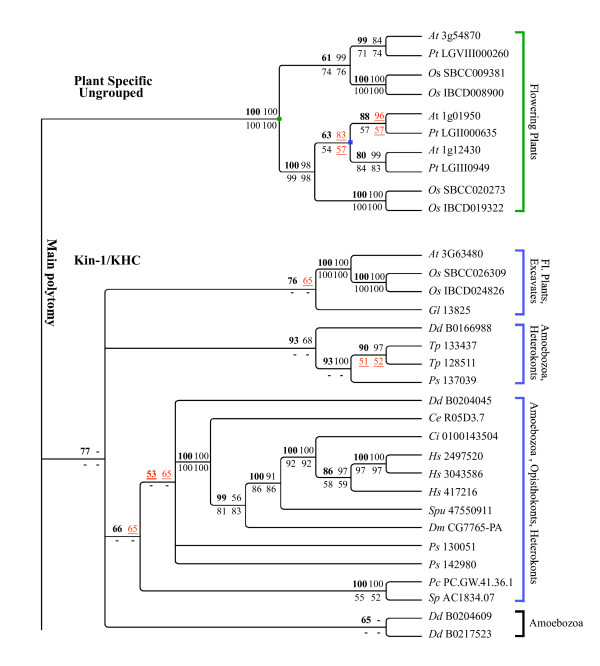
**Expanded view of plant-specific ungrouped kinesins and Kinesin-1 family**. Expansion of the plant-specific ungrouped and Kinesin-1 clades from Fig. 2. Green brackets indicate plant specific groups, mixed clades are shown in blue brackets and black brackets indicate protozoan species. Green circle and blue square indicate gene duplications in flowering plants and dicots, respectively. See Fig. 2 legend for an explanation of support values. Support values in italicized blue font indicate those clades supported in the parsimony analyses except for the exclusion of *Ps *128382 (see text). For full names of species see Fig. 1 legend. Fl. Plants, flowering plants.

**Figure 4:**
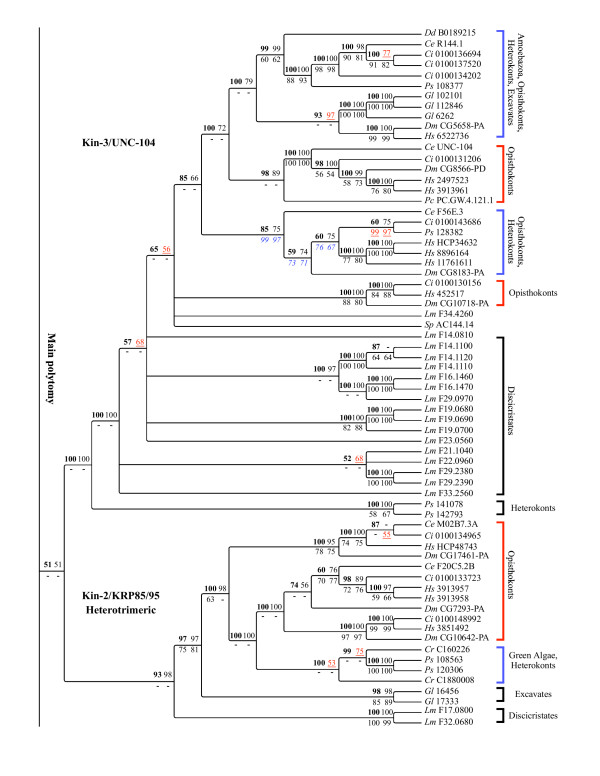
**Expanded view of Kinesin-2 and Kinesin-3 families**. Expansion of the Kinesin-2 and Kinesin-3 families from Fig. 2. There are no plant kinesins present in either of these families. Red brackets indicate opisthokonts, mixed clades are shown in blue brackets and black brackets indicate protozoan species. See Fig. 2 legend for an explanation of support values. For full names of species see Fig. 1 legend.

**Figure 5:**
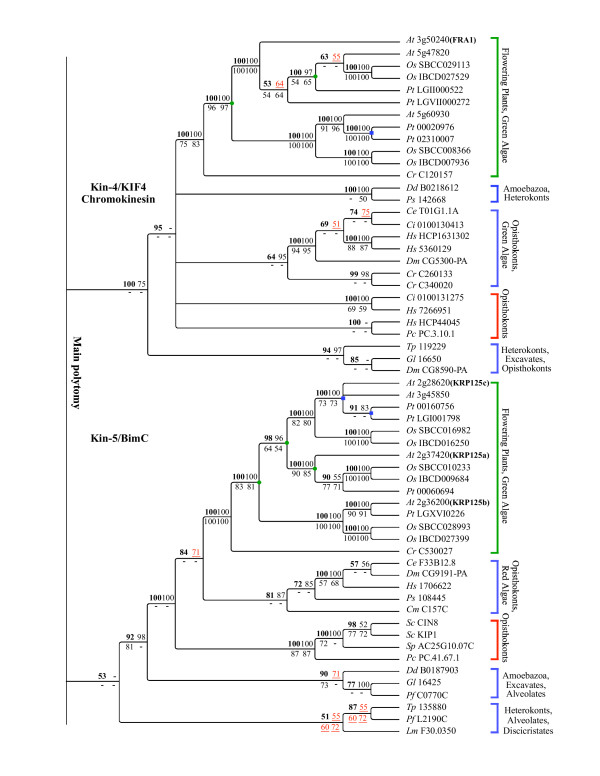
**Expanded view of Kinesin-4 and Kinesin-5 families**. Expansion of the Kinesin-4 and Kinesin-5 families from Figure 2. Experimentally studied Arabidopsis kinesins are indicated in parenthesis with their published names in bold. Green brackets indicate plant-specific groups, mixed clades are shown in blue brackets and red brackets indicate groupings composed of opisthokonts. Green circles and blue squares indicate gene duplications in flowering plants and dicots, respectively. See Fig. 2 legend for an explanation of support values. For full names of species see Fig. 1 legend.

**Figure 6:**
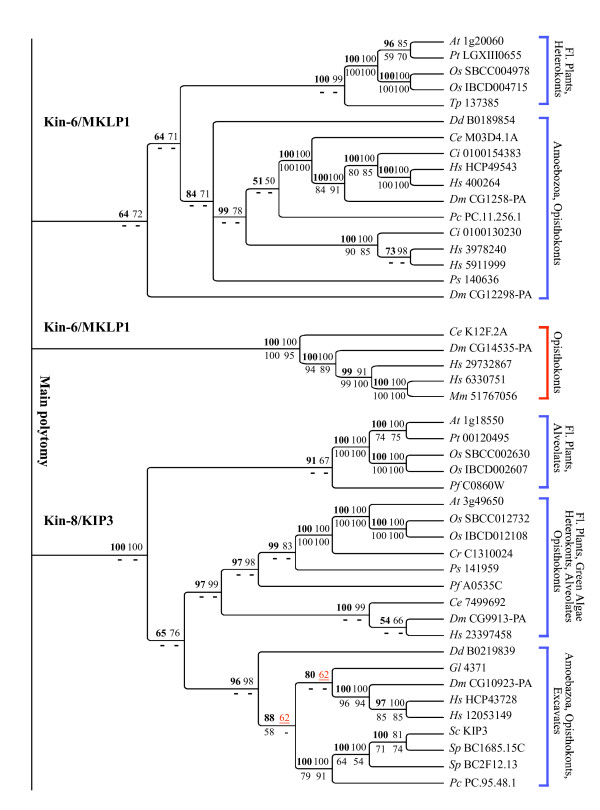
**Expanded view of Kinesin-6 and Kinesin-8 families**. Expansion of the Kinesin-6 and Kinesin-8 families from Fig. 2. Mixed clades are shown in blue brackets and red brackets indicate groupings composed of opisthokonts. See Fig. 2 legend for an explanation of support values. For full names of species see Fig. 1 legend. Fl. Plants, flowering plants.

**Figure 7:**
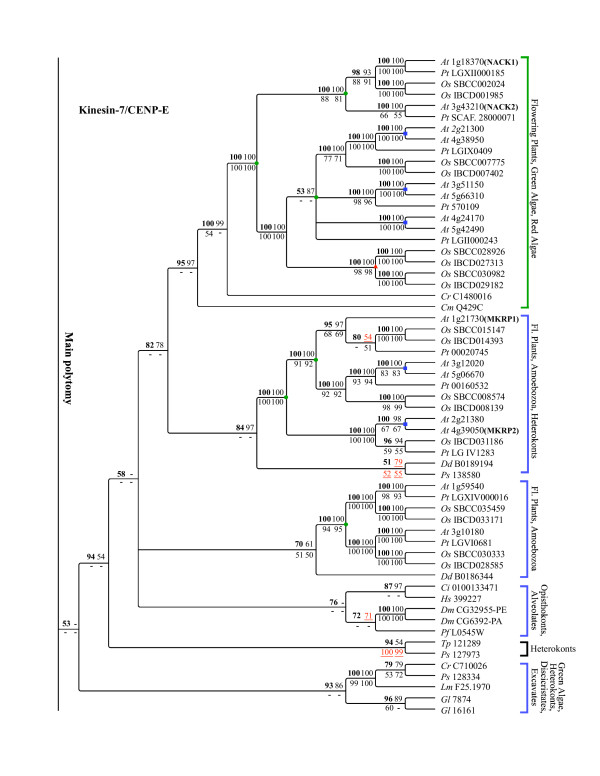
**Expanded view of Kinesin-7 family**. Expansion of the Kinesin-7 family from Fig. 2. Experimentally studied Arabidopsis kinesins are indicated in parenthesis with their published names in bold. Green brackets indicate plant specific groups, mixed clades are shown in blue brackets and black brackets indicate protozoan species. Green circles, blue squares and a red triangle indicate gene duplications in flowering plants, dicots and monocots, respectively. See Fig. 2 legend for an explanation of support values. For full names of species see Fig. 1 legend. Fl. Plants, flowering plants.

**Figure 8:**
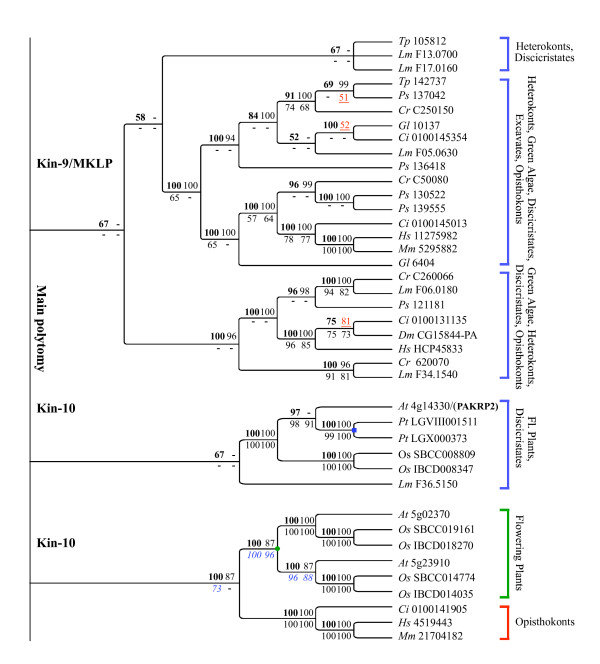
**Expanded view of Kinesin-9 and Kinesin-10 families**. Expansion of the Kinesin-9 and Kinesin-10 families from Fig. 2. An experimentally studied *Arabidopsis *kinesin is indicated in parenthesis with its published name in bold. Green brackets indicate plant-specific groups, mixed clades are shown in blue brackets and red brackets indicate groupings composed of opisthokonts. Green circle and blue square indicate gene duplications in flowering plants and dicots, respectively. See Fig. 2 legend for an explanation of support values. Support values in italicized blue font indicate those clades supported in the parsimony analyses except for the inclusion of *Pt *00151235 (see text). For full names of species see Fig. 1 legend. Fl. Plants, flowering plants.

**Figure 9:**
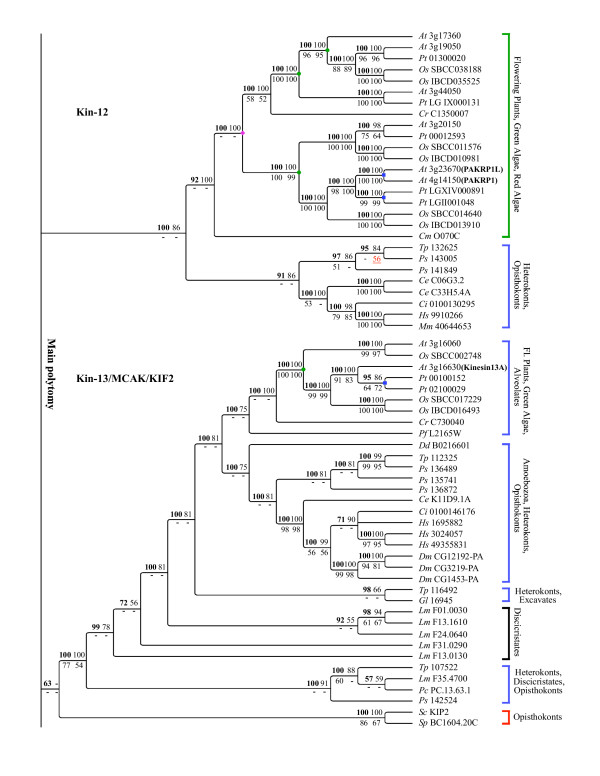
**Expanded view of Kinesin-12 and Kinesin-13 families**. Expansion of the Kinesin-12 and Kinesin-13 families from Fig. 2. Experimentally studied *Arabidopsis *kinesins are indicated in parenthesis with their published names in bold. Green brackets indicate plant specific groups, mixed clades are shown in blue brackets, black brackets indicate protozoan species and red brackets indicate groupings composed of opisthokonts. Purple diamond indicates a gene duplication after the divergence of red and green algae from one another yet prior to the divergence of green algae from flowering plants. Green circles and blue squares indicate gene duplications in flowering plants and dicots, respectively. See Fig. 2 legend for an explanation of support values. Support values in italicized blue font indicate those clades supported in the parsimony analyses except for the exclusion of *Pt *00151235 (see text). For full names of species see Fig. 1 legend. Fl. Plants, flowering plants.

**Figure 10:**
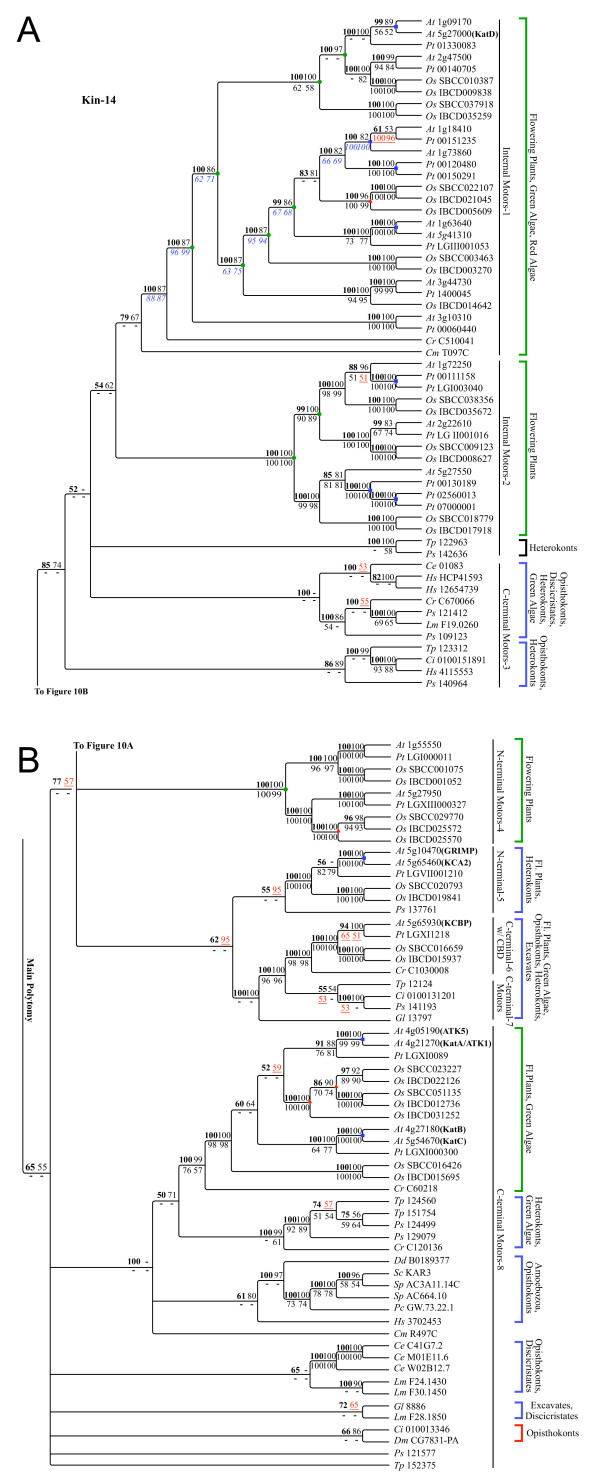
**Expanded view of Kinesin-14 family**. Expansion of the Kinesin-14 family from Fig. 2. Motor domain localizations are indicated adjacent to taxon labels. Experimentally studied Arabidopsis kinesins are indicated in parenthesis with its published name in bold. Green brackets indicate plant specific groups, mixed clades are shown in blue brackets, black brackets indicate protozoan species and red brackets indicate groupings composed of opisthokonts. Green circles, blue squares and a red triangle indicate gene duplications in flowering plants, dicots and monocots, respectively. See Fig. 2 legend for an explanation of support values. For full names of species see Fig. 1 legend.

The abbreviated unrooted Bayesian tree for the amino-acid-plus-gap-characters analysis is presented in Fig. 2 as our best estimate of the relationships within the kinesin gene family. The expanded view of kinesin subfamilies is presented in Figures 3 to 10. This analysis was favored over those that did not incorporate gap characters because it incorporated all available characters from the motor domain and is, therefore, favored by the total-evidence criterion [60,61]. Furthermore, inclusion of the gap characters increased both the number of clades resolved (439 → 453 Bayesian; 288 → 296 parsimony) and average branch support (90.3% → 92.0% Bayesian; 87.3% → 87.5% parsimony) in both the Bayesian and parsimony analyses, which is consistent with the general patterns found by Simmons *et al*., [62]. The Bayesian analysis was favored over the parsimony analysis because the inferred Bayesian tree is more resolved than the parsimony tree and the additional resolution is largely congruent with previous analyses with respect to resolution of the 14 kinesin families. The other three gene trees from both Bayesian and parsimony analyses are available as supplemental data (see Additional files 18 to 20).

Each branch of the Bayesian tree in Fig. 2 indicates the posterior probability from the amino-acid-plus-gap-characters analysis at the upper left of the branch. To allow for inspection of the support for each branch provided by the other analyses, the posterior probability from the amino-acid-characters-only analysis is at the upper right of each branch, the parsimony jackknife support from the amino-acid-plus-gap-characters analysis is at the lower left, and the parsimony jackknife support from the amino-acid-characters-only analysis is at the lower right. If a branch was unresolved in one of these other three analyses, it is indicated by "-" at the respective location. If a branch was contradicted in one of these other three analyses, it is indicated by underlined red font at the respective location with the single highest posterior probability or jackknife support value for the contradicting clade(s) shown.

### Conflict between parsimony and Bayesian trees

There were three cases of well supported, conflicting resolution in which both Bayesian trees were contradicted by both parsimony trees. First, in the parsimony trees *Pt *00151235 was well supported as nested within the second Kinesin-10 clade, whereas it was well supported as nested within the Kinesin-14 clade in the Bayesian trees. Aside from this single sequence, the Kinesin-10 and Kinesin-14 clades were largely congruent in all four trees (Figs. 8, 10). The parsimony resolution is supported by the Kinesin-10-specific domains that *Pt *00151235 has, whereas the sequence lacks the Kinesin-14-specific domains. Therefore, we favor the parsimony resolution of *Pt *00151235 as a member of the Kinesin-10 family (Table 4). Second, in the parsimony trees *Tp *121289 was well supported as the sister group of *Tp *163717, whereas the clades of (*Tp *121289, *Ps *127973) and (*Tp *163717, *Lm *F22.0560) were well supported in the Bayesian trees with the former clade resolved with the Kinesin-7 family (Figs. 2, 7). There are no additional domains of *Tp *121289 or *Tp *163717 to distinguish between these alternative resolutions. Third, in the parsimony trees *Ps *128382 was unresolved in the main polytomy, whereas it was well supported as nested within the Kinesin-3 clade in the Bayesian trees (Fig. 4). The Bayesian resolution is supported by the Kinesin-3-specific domain that *Ps *128382 has.

To test for long-branch attraction [63] in the parsimony analyses between *Pt *00151235 and the second Kinesin-10 clade, the amino-acid-plus-gap-characters parsimony jackknife analysis was repeated after eliminating all nine other sequences in the second Kinesin-10 clade [64]. However, this explanation was not supported because *Pt *00151235 was unresolved in the main polytomy rather than moving to within the Kinesin-14 clade. Likewise, to test for long-branch attraction in the parsimony analyses in the second case of conflicting resolution, the parsimony jackknife analysis was repeated after eliminating *Tp *163717. However, this explanation was not supported because both *Tp *121289 and *Ps *127973 were unresolved in the main polytomy rather than being resolved as sister groups. Therefore, we were unable to discard either of the two alternative hypotheses regarding the relationships of *Tp *121289 and *Tp *163717. Because *Ps *128382 was unresolved in the main polytomy on the parsimony trees, it was not possible to apply this test to it.

### Many new kinesins are not grouped into recognized kinesin families

All 14 currently recognized kinesin families [14] are represented in our inferred gene tree (Fig. 2). All members in each family are presented in Figs. 3 to 10. However, 11% of the sequences (58 of 529 sequences) were not resolved among the lineages corresponding to previously recognized families (Fig. 2). Most of these kinesins are from *Leishmania*, *Giardia*, *Phytopthora*, *Chlamydomonas *and *Thallassiosira*. These sequences may represent novel kinesin families and/or early-derived members of the 14 recognized kinesin families that are not resolved as such in our inferred gene tree. Several clades that are not part of the known families contained members from two eukaryotic groups, indicating that they are not unique to one of the eight main eukaryotic lineages [48]. A strongly supported clade of ten kinesins (Figs. 2, 3 plant-specific ungrouped) from flowering plants were not resolved into any of the 14 kinesin families. However, these formed a distinct clade (Fig. 2 and Fig. 3 plant-specific ungrouped) with strong support values from all four analyses. Interestingly, the members of this group also have an armadillo domain that is not present in any invertebrates or vertebrates (see domain analysis section below).

### At least three of the 14 kinesin families are absent in flowering plants

The distribution of kinesin families in the 19 species sampled is shown in Fig. 1. Some families (e.g. Kinesin-5; Kinesin-13 and Kinesin-14) are present in almost all of the main eukaryotic lineages. Although flowering plants have the largest number of kinesins, at least three of the 14 families are conspicuously absent in flowering plants. It is unclear whether either three or four (Kinesin-2, Kinesin-3, Kinesin-9 and/or Kinesin-11) of these 14 families are absent in flowering plants because of the entirely unresolved flowering-plant clade (Figs. 2 and 3). Members of Kinesin-2 form either homo- or heterodimers and are present in ciliated and flagellated cells and function in organelle-intraflagellar transport [2,9]. Interestingly, the flagellated unicellular photosynthetic eukaryote *Chlamydomonas *has one Kinesin-2, which is also involved in intraflagellar transport [65]. Since flowering plants lack cilia or flagella in their life cycle, this family of kinesins is lost in this lineage. It would be interesting to see if the land plants that have ciliated/flagellated cells in their life cycle (e.g., bryophytes, pteridophytes and gymnosperms) retained this family of kinesins. Unfortunately, genomes of plants that belong to these groups have not been sequenced to address this. Members of the Kinesin-3 family are involved in organelle transport. The Kinesin-3 family is expanded in animals with seven members in humans, the largest of any family. Interestingly, 17 of the 54 *Leishmania *kinesins are grouped within the Kinesin-3 family and this grouping is strongly supported by both Bayesian analyses. It appears that lineage-specific duplication of genes contributed to expansion of this family. The fork-head-associated (FHA) domain present in vertebrate members of this family is absent in all Kinesin-3 members of *Leishmania*, suggesting that the acquisition of this domain occurred after the divergence of the *Leishmania *and animal lineages. The Kinesin-1 family, which is also involved in transport of vesicles, is underrepresented in plants. It was previously speculated [43] that there might be a higher plant Kinesin-1/KHC family member but it was not conclusive. It appears that Arabidopsis and *O. sativa *have one Kinesin-1/KHC gene and the diatom has two, whereas there may not be a Kinesin-1 in *Chlamydomonas*. Overall, cargo-transporting kinesins are either absent (Kinesin-2 and Kinesin-3) or underrepresented (Kinesin-1) in flowering plants. The cargo-transport functions of some of these kinesins are either not needed in flowering plants or performed by members of other families of kinesins or cargo transporting myosins, which are expanded in plants [2,66]. Although Kinesin-9 family members are absent in flowering plants, they are present in two photosynthetic eukaryotes (four in *Chlamydomonas *and two in diatoms). It appears that this family is lost in flowering plants. The functions of the Kinesin-9 family are unknown [9]. Members of the Kinesin-11 family function in signal transduction and contain a few highly divergent kinesins and are absent not only in plants but in many other lineages [67,68].

Forty *indica *kinesins have orthologs in *japonica *and three of these (IBCD021045, IBCD025572 and IBCD012736 in kinesin 14 family, see Figure 10A&B) are duplicated in *indica *only. The duplications found in *indica *may have occurred recently or *japonica *sequence may not be complete. In addition, two kinesins in *indica *(IBCD031186 in kinesin 7 family, see Figure 7; IBCD014642 in kinesin 14 family, see Figure 10A) have no counterparts in *japonica *whereas one kinesin in *japonica *(SBCC002748, see Figure 9) has no counterpart in *indica*, suggesting that either a specific kinesin is lost in one cultivar or it is due to differences in unsequenced gaps in the genome of these two rice cultivars.

### Two families (Kinesin-7 and Kinesin-14) are vastly expanded in plants

Kinesin-14, the C-terminal motor family, and the Kinesin-7 family have greatly expanded in plants through gene duplication. Kinesin-14 is a diverse family containing eukaryotic representatives from almost all major eukaryotic groups; therefore, the members of this family are likely to play important evolutionarily conserved cellular roles. Kinesin-14 family members show minus-end motility and perform multiple functions in cell division and organelle transport [9,18]. Kinesin-14 is the largest family of kinesins in flowering plants (about 35% of Arabidopsis kinesins fall in this group). Flowering plant kinesins in this family contain several domains that are not present in non-plant kinesins. In the Kinesin-14 family, there are several subfamilies in which the motor domain is located in the middle or at the N or C terminus. It is interesting to observe the many plant-specific kinesin duplications (Fig. 10). Based on the inferred phylogenetic relationships among the plant species sampled [50,69-71], we infer a minimum of nine duplications in the top clade of flowering-plant lineage (see 10A, internal motor-1 and -2) prior to the divergence of monocots from dicots, seven duplications within the dicot lineage, and one duplication within the monocot lineage (see Fig. 10A). Because the green and red algae Kinesin-14 sequences are resolved as sister to the upper Kinesin-14 flowering-plant clade, we also infer that there was a plant-specific duplication prior to the divergence of the red and green algae. One of the two copies was then lost in both the red and green algae lineages, yet retained in the flowering-plant lineage.

The first subfamily of the Kinesin-14 family has plant-specific kinesins that are shared by the both the green and the red algae (Fig. 10A). Members of flowering plants in this group have a calponin-homology (CH) domain that is not present in green or red algae, suggesting that this domain was gained in the flowering-plant lineage (see domain analysis section). The second group that is well supported by all four analyses only includes kinesins from flowering plants (Fig. 10A). Interestingly, members of these two top most groups have an internal motor domain instead of the C-terminal motor domain for which this family is named. Another group that is restricted to the plant kingdom deals with the N-terminal flowering plant-specific group in the Kinesin-14 family (See Fig. 10B). Perhaps the N-terminal and internal motor groups in the family could have arisen in flowering plants by domain shuffling of a C-terminal motor. The members of the seventh group with a C-terminal motor contain a myosin tail homology 4 (MyTH4) region and talin-like region (also known as Band 4.1 or FERM) that are not present in any non-plant kinesins. The last large subfamily of the Kinesin-14 family is reflective of true C-terminal motors in plants, diatoms, animals and protozoans (Fig. 10B). From this analysis it appears that the Kinesin-14 family is composed of multiple subfamilies instead of the two previously reported Kinesin-14A and Kinesin-14B families [14]. Based on the location of the motor and the presence of other domains, there could be up to eight subfamilies within the Kinesin-14 family (five of which are likely to be plant-specific). There appears to be several dicot- and monocot-specific duplications in plant kinesins of this family. Several of the subfamilies resulted primarily due to the emergence of novel kinesins in the plant lineage. The members of this family with the C-terminal motor domain have been shown to be minus-end motors [8,72]. Although plant motors with a C-terminal domain translocate toward the minus-end of MTs [39-41], it is not known if the internal and N-terminal plant motors are also minus-end motors. The functions of several Arabidopsis Kinesin-14 family members that contain the motor domain at the C terminus (e.g., KatA/ATKl [*At*4g21270], ATK5 [*At*4g05190], KCBP [*At*5g65930]), N terminus (GRIMP/KCA1 [*At*5gl0470]) and KCA2 [*At*5g65460]) or in the middle (KatD [*At5g*27000]) have been analyzed. Several of these (ATK1, ATK5, KCBP) are involved in some aspect of cell division [20,23,24,28,29,31], suggesting that the plant kinesins of this family play important roles in cell division. The cotton homolog of Arabidopsis KatD localizes to cortical MTs and microfilaments and interacts directly with F-actin [38]. GRIMP/KCA1 interacts with a geminivirus replication protein and localizes to segregating chromosomes and spindle poles [37]. Both GRIMP/KCA1 and KCA2 interact with a cyclin-dependent kinase (CDKA;1), which controls cell cycle progression, and localizes to MTs and phragmoplast [73], suggesting a role for these also in cell division. The fact that plants have unique MT arrays such as the preprophase band and phragmoplast that play critical roles in plant cell division, lack centrosomes to organize MTs to establish a bipolar spindle [1] and have no (or few) dyneins, [74,75], which are also minus-end motors, suggests that plants would require novel kinesins to perform these plant-specific roles and to cover the functions performed by dyneins in animals. In addition, several reports indicate that plants transport macromolecules (e.g. RNA and proteins) and viruses form cell to cell through plasmodesmata [1,76]. Such transport activities may also require kinesins. Hence, it is possible that the expansion of kinesins in plants accounts for the need for plant-specific motors in flowering plants.

Kinesin-7/CENP-E is the second largest kinesin family in plants and one clade (plant-specific clade in Fig. 7) contains kinesins from only photosynthetic eukaryotes with a green algal kinesin as a sister group to those from the flowering plants. The flowering-plant-specific clade is strongly supported by both Bayesian and parsimony analyses. Hence, multiple members in a species in this clade (e.g., seven in Arabidopsis) are inferred to have arisen by at least six gene duplications in the flowering-plant lineage prior to the divergence of monocots from dicots, five in the dicot lineage, and one in the monocot lineage. The functions of two members of this clade have been reported. Kinesins encoded by *At*1g18370 (also called NACK1/HINKEL) [25,26] and *At*3g43210 (also called STUD/TETRASPORE/NACK2) [25,27] encode functionally related motors. Loss-of-function mutants of these kinesins revealed their role in cytokinesis [25-27,77]. Interestingly the NACK1 activates a MAP kinase (MAPK) [25]. The second clade in this family also contains flowering plants, amoebozoa and heterokonts, but not opisthokonts (vertebrates, invertebrates and fungi). Two Arabidopsis members of this group are targeted to mitochondria [36], implying an unknown function for these kinesins in this organelle. The third group contains kinesins from flowering plants and amoebozoa. Members of these groups are inferred to be more closely related to one another than to the small, animal subfamily. Because of extensive duplication in the CENP-E family in plants, the members of this family may have been recruited to perform plant-specific functions. In animals, members of this family function in capturing kinetechore MTs. Studies with some members of plant kinesins that belong to Kinesin-7 indicate their role in cytokinesis and some unknown function in mitochondria [36,77]. Overall, our analyses indicate that there was tremendous diversification in Kinsin-14 and Kinesin-7 families in flowering plants.

### Seven of the ten kinesin families in flowering plants are present in *Chlamydomonas*

*Chlamydomonas*, a member of chlorophyte algae, represents the sister group of the flowering plants given our taxon sampling [46,50]. Hence, the analysis of kinesin families in this species should provide some insights into evolution of kinesins in flowering plants. *Chlamydomonas*, despite the fact that it is unicellular, has 23 kinesins (Table 3). Twenty of these were grouped into nine recognized families whereas the remaining three are ungrouped (Table 3). Kinesin-1, -3, -6, -10 and -11 families are absent in *Chlamydomonas*. Of the ten kinesin families present in flowering plants, seven are present in *Chlamydomonas *(Fig. 2). Three families (Kinesin-1, -6 and -10) of flowering plants are inferred to have been lost in the *Chlamydomonas *lineage. Two families (Kinesin-2 and Kinesin-9) that are present in *Chlamydomonas *were lost in the flowering-plant lineage. One of these families (Kinesin-2) is involved in intraflagellar transport. As mentioned above, the absence of flagella/cilia in flowering plants may have resulted in the loss of this family.

**Figure 11:**
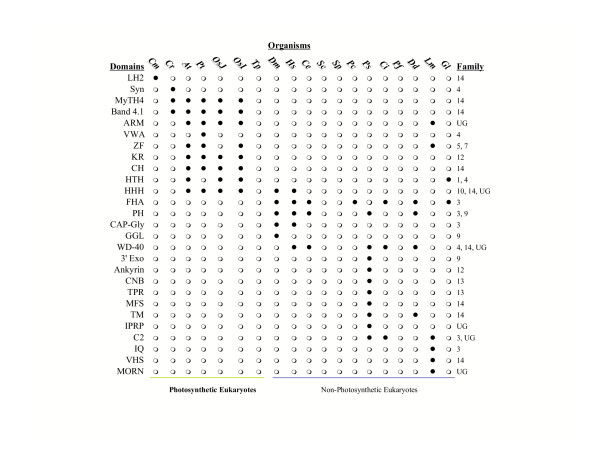
**Distribution of domains found within kinesins of 19 species**. Schematic showing the presence or absence of particular domains and which families these domains are found in across the 19 species sampled. Species are listed horizontally across the top of the figure with the various domains listed vertically. Presence of a domain is indicated by a solid circle and its absence by a hollow circle. LH2, Lipoxygenase; Syn, N-terminal Syntaxin; MyTH4, Myosin Tail Homology 4; ARM, Armadillo; VWA, Von Willebrand Factor, Type A; ZF, Zinc finger; KR, Kinesin-Related; CH, Calponin Homology; HTH, Helix-Turn-Helix; HHH, Helix-Hairpin-Helix; FHA, Fork Head Associated; PH, Pleckstrin homology; CAP-Gly, Glycine rich domain found in Cytoskeleton Associated Proteins (CAPs); GGL, G-protein gamma motif like; WD-40, A 40 amino acid repeat motif with W and D dipeptides at the terminus; 3' Exo, 3' Exoribonuclease; CNB, Cyclic Nucleotide Binding; TPR, Tetratricopeptide repeat; MFS, Major Facilitator Superfamily; TM, Transmembrane; IPRP; Inositol polyphospate related phosphatase; C2, Protein kinase C conserved region 2; VHS, Domain present in Vps-27, Hrs, STAM; MORN, Membrane occupation and recognition nexus. In *T. pseudonana*, most kinesins are short (Table 7 and Figure 12), which could be due to poor quality of the gene models. Hence, it is possible that they contain additional domains. For full names of species see Fig. 1 legend.

The red alga (*C. merloae*) has only five kinesins that belong to four families (Table 2) whereas the diatom (*T. pseudonana*) has 25 kinesins (about the same number as in the green alga) that fall into nine known kinesin families with four kinesins unresolved (Table 7). Although the green alga and the diatom have nine families, unlike *Chlamydomonas*, the diatom has Kinesin-1 and Kinesin-6 but may not have members of the Kinesin-2 or Kinesin-8 families. Remarkably, Kinesin-14 is the largest family in all photosynthetic eukaryotes. Among the 14 recognized families, only four (Kinesin-5, -7, -12 and 14) were shown to be present in all photosynthetic eukaryotes (Fig. 11). The absence of myosins and dyneins in the red alga (C. *merolae*) suggests that kinesins play important roles in this species [48,78].

### *Giardia*, an early-derived eukaryote, has ten of the fourteen kinesin families

In *Giardia *there are 24 kinesins (see Additional file 12). This is more than half the number of kinesins found in humans [6]. However, *Giardia *has no recognizable myosin [48], suggesting that kinesins perform most of the transport functions. Sixteen of the 24 kinesins in *Giardia *were resolved into ten known families whereas the remaining eight were unresolved (see Additional file 12). If *Giardia *is indeed part of the earliest derived extant lineage of the eukaryotes and therefore existed prior to the plant-animal split [44,46,47] the ten families with representatives in *Giardia *are inferred to represent the basic set of kinesin families in early eukaryotes. The families that are not represented in *Giardia *are Kinesin-6, -10, -11 and -12. Hence, these families may have emerged later in eukaryotic evolution through gene duplication. Four of the ungrouped kinesins in *Giardia *did not group with kinesins from other species whereas the remaining four grouped either with *Leishmania *or *Chlamydomonas *kinesins (see Fig. 2). Many of the domains found in flowering plants and animals are not present in *Giardia *(Fig. 11).

### Other kinesin families in plants

All flowering-plant kinesins from the Kinesin-4 family form a well-supported clade as the sister group of a *Chylamydomonas *sequence (Fig. 5). Two *Chlamydomonas *kinesins in this family did not group with flowering plants, though this resolution was not supported in the parsimony analyses (Fig. 5). A member of this family is involved in cell wall deposition [35]. Kinesin-5 family motors function in cell division and spindle formation [79]. All flowering-plant kinesins of the Kinesin-5/BIMC family form a well-supported clade sister to the single *Chlamydomonas *sequence (Fig. 5). Plant members of this family, like their animal counterparts, are likely to function in cell division [80]. Members of the Kinesin-6 family that function in cytokinesis in animals are not inferred to have undergone any gene duplications in plants (in which both copies have been retained as functional genes). There is only one kinesin of this family in each of the flowering plant species analyzed here and none in the green or red algae. Since cytokinesis in plants is quite different from animals [1], it appears that members of other kinesins families perform this function. Kinesin-8 members are found in plants, fungi and animals. *Oryza *and *Arabidopsis *have two Kinesin-8 genes whereas *Populus *has one (Fig. 6). The non-plant members function in nuclear migration and mitochondrial transport. The function of plant members of this family remains unknown. Plant kinesins associated with the Kinesin-10 family were resolved as two separate clades from the main polytomy (Fig. 8) indicating that two copies of Kinesin-10 were present in flowering plants prior to the divergence of monocots from dicots.

The Kinesin-12 family members function in organelle transport [9]. This family includes kinesins from both plants and animals. There are multiple members of this family in each flowering plant species. The flowering plant members formed two distinct clades, one as a sister group to *Chlamydomonas*, with the red algae sequence as sister to both (Fig. 9). Based on this resolution, we infer at least one gene duplication after the divergence of red and green algae from one another yet prior to the divergence of green algae from flowering plants, three duplications in the flowering-plant lineage prior to the divergence of monocots from dicots, and two duplications within the dicot lineage. Two plant members of this group localize to a plant-specific structure called the phragmoplast [22]. Members of the Kinesin-13 family, most of which have internal motors, transport vesicles and have MT depolymerizing activity [81,82]. Plant members of this family also form a distinct clade with the *Chlamydomonas *kinesin as a sister group (Fig. 9). These internal-motor plant kinesins are distinct from the other internal-motor plant kinesins (found only in plants) in the Kinesin-14 family.

### Domain analysis

**Figure 12:**
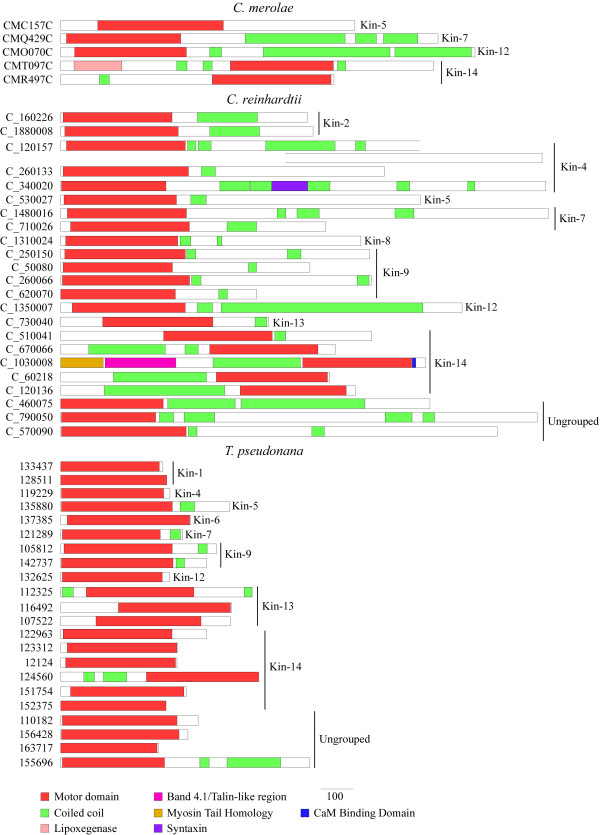
**Domain schematics for unicellular photosynthetic eukaryotes**. Schematic diagrams showing the domain architecture of kinesins in *C. merolae, C. reinhardtii *and *T. pseudonana*. Family classifications are shown to the right of the proteins.

**Figure 13:**
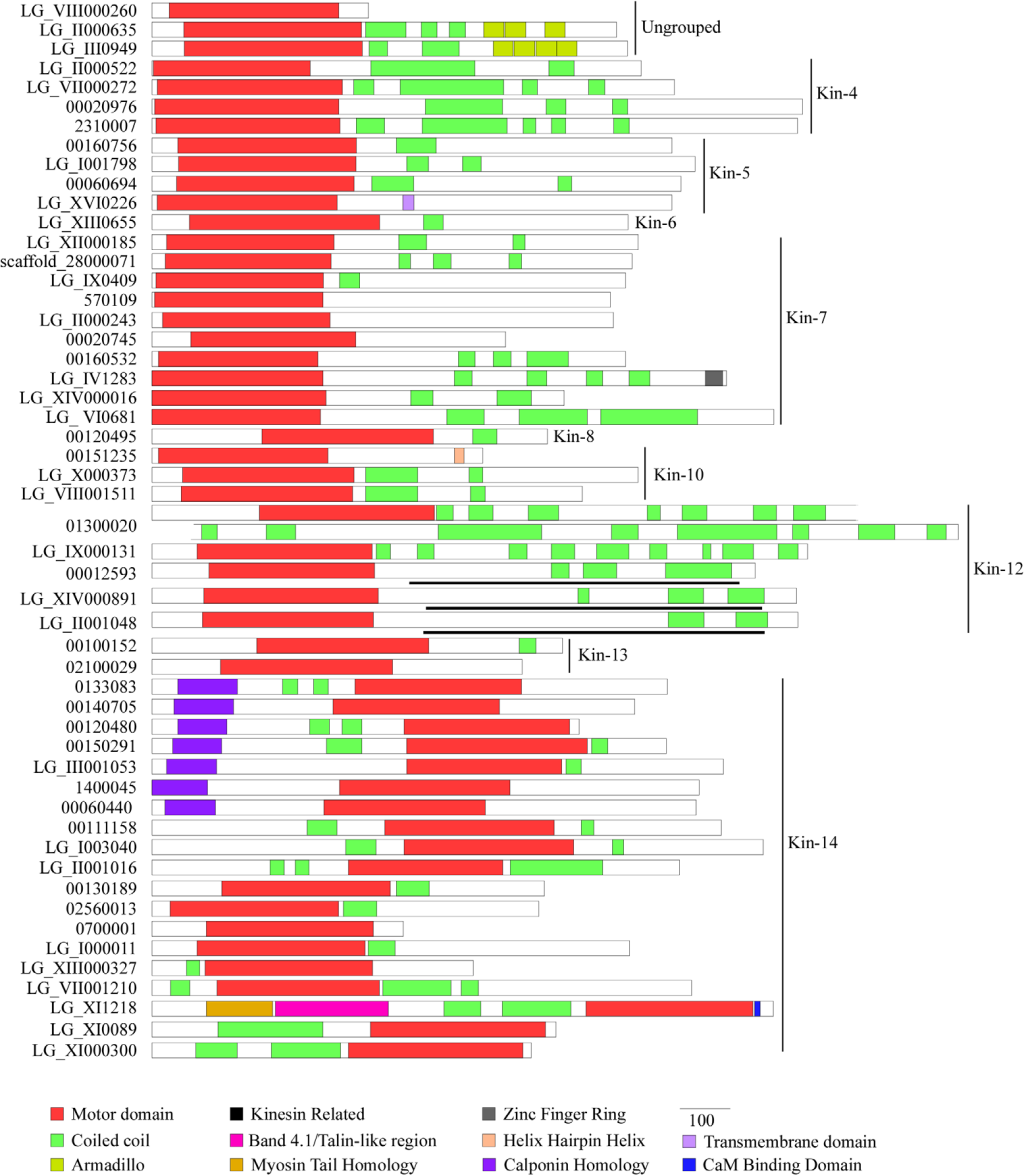
**Domain schematics for *P. trichocarpa***. Schematic diagram showing the domain architecture of kinesins in *P. trichocarpa*. Family classifications are shown to the right of the proteins.

**Figure 14:**
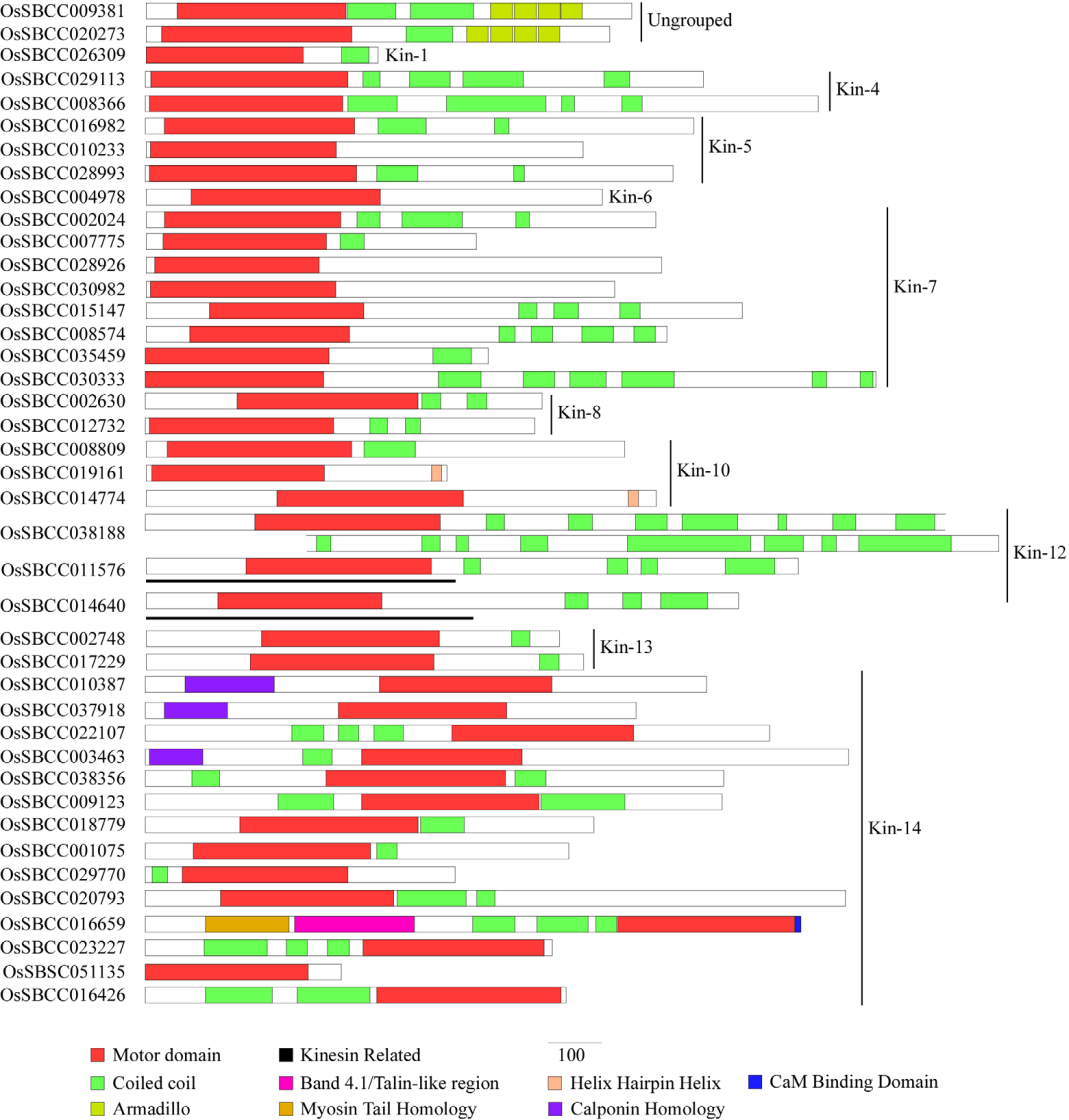
**Domain schematics for *O. sativa ssp. japonica***. Schematic diagram showing the domain architecture of kinesins in *O. sativa *ssp. *japonica*. Family classifications are shown to the right of the proteins.

Domain analysis was performed on all eukaryotes used in this study as described in the Methods section. The most prevalent domain is the coiled-coil region; almost every kinesin sequence analyzed has a coiled-coil prediction (based on the SMART algorithm [83] (see Tables 2, 3, 4, 5, 6, 7 and Additional files 1 to 12). Among the kinesins analyzed here, about 30 known functional domains (not including the motor domain and coiled-coil region) are found. Fig. 11 shows the list of functional domains and their presence in various species. A schematic diagram of kinesins depicting all the domains in the green alga, red alga and diatom are shown in Fig. 12 whereas domain figures of *Populus *and one species of *Oryza *are shown in Figs. 13 and 14, respectively. Domains in Arabidopsis kinesins were reported previously [43]. Various known domains in non-plant systems are indicated in Additional files 1 to 12. Interestingly, not a single domain is present in all kinesins. Instead, most domains are restricted to a particular lineage (Fig. 11) suggesting that most of these are gained later in evolution and have novel functions. This is also supported by the fact that in *Giardia *most of these domains (except fork head associated and helix-turn-helix domains) are absent. Some domains such as myosin tail homology domain 4 (MyTH4) and band 4.1 (also called talin-like region or FERM) are restricted to green algae and flowering plants (Fig. 11). Although MyTH4 and band 4.1 domains are present in several animal proteins including some myosins, they are not found in non-plant kinesins. Interestingly, in Arabidopsis the MyTH4 and band 4.1 are present in one kinesin and are not present in any other protein encoded in the genome [83]. We have previously shown that MyTH4 and talin-like regions are involved in binding to MTs [84], suggesting that it may be involved in cross-linking and/or bundling MTs. It was recently shown that MyTH4 and band4.1 in myosins also bind MTs [85], hence these domains are likely to function in cross-linking actin and MT cytoskeleton and/or transfer of cargo between two different cytoskeletal elements.

Calponin homology (CH) and kinesin-related (KR) domains are found in flowering plants but not in green and red algae or heterotrophs (Fig. 11). There are several kinesins with one CH domain and a KR domain in each flowering plant analyzed here (see Figs. 13 and 14). All CH domain kinesins belong to the Kinesin-14 family and were resolved as the first clade in this family (see 10A). The only other protein family that has the CH domain is fimbrin. Plant fimbrins have four copies of the CH domain and bind F-actin. The CH domain is a protein module of about 110 residues found in cytoskeletal and signal transduction proteins either as a single copy or multiple copies in tandem. Proteins with a tandem pair of CH domains cross-link F-actin, bundle actin or connect intermediate filaments to cytoskeleton [86]. Proteins with a single copy are involved in signal transduction [87]. Although plant kinesins have only one CH domain, recently it was shown that a kinesin with this domain interacts with F-actin, suggesting that the kinesins with this domain may be involved in interaction between actin and MT cytoskeleton [38]. The function of the KR domain is not known. However, the kinesins with this domain associate with the phragmoplast [21,22] and belong to the Kinesin-12 family (Fig. 9). Several flowering-plant kinesins and one non-plant (*Leishmania*) kinesin have armadillo/betacatenin-like (ARM) repeats that are known to mediate protein-protein interactions. Diverse proteins contain ARM repeats that form a superhelix of helices and function in intracellular signaling and cytoskeletal regulation. Although none of the vertebrate and invertebrate kinesins have an ARM, a Kinesin-2 family-associated protein called KAP3 in animals contains the ARM repeat [2,88]

Animal kinesins have some domains that are not found in photosynthetic eukaryotes. These include fork-head associated (FHA), pleckstrin homology (PH), CAP-Gly domains and WD-40 repeats. The FHA domain is known to interact with phosphothreonine in proteins. Cap-gly is a glycine-rich domain of about 40 amino acids that is found in cytoskeleton-associated proteins (CAPs). The WD-40 repeats are also short (about 40 residues) motifs that often terminate in Trp-Asp (W-D) dipeptide and facilitate the formation of multi-protein complexes. Two of these domains (FHA and PH) are present in several protozoans (Fig. 11), suggesting that these domains may have been present in kinesins in the most recent common ancestor of all extant eukaryotes.

The only domain common to both plants and animals (both invertebrates and vertebrates) is the helix-hairpin-helix (HHH) DNA binding motif in the Kinesin-10 family that functions in chromosome segregation (Fig. 11). A member of this family has been shown to bind DNA ([89] and it is likely that others with this domain also bind DNA and function in chromosome segregation. However, the HHH domain is not present in fungi or the green or red algae, suggesting that plant and animals may have acquired this domain independently. Overall, the domain distribution in kinesins suggests several domains were added to plant and animal kinesins after the plant-animal split. Interestingly, there are several domains present only in either *Leishmania *or *Phytopthora sojae*, which suggests tremendous diversification of kinesins in lower eukaryotes that may have to do with their unique life cycle and cell biology. In diatom, most kinesins are short (Table 7 and Figure 12), which could be due to poor quality of the gene models.

### Genome duplication in flowering plants may have contributed to the expansions of kinesins in this group

**Figure 15:**
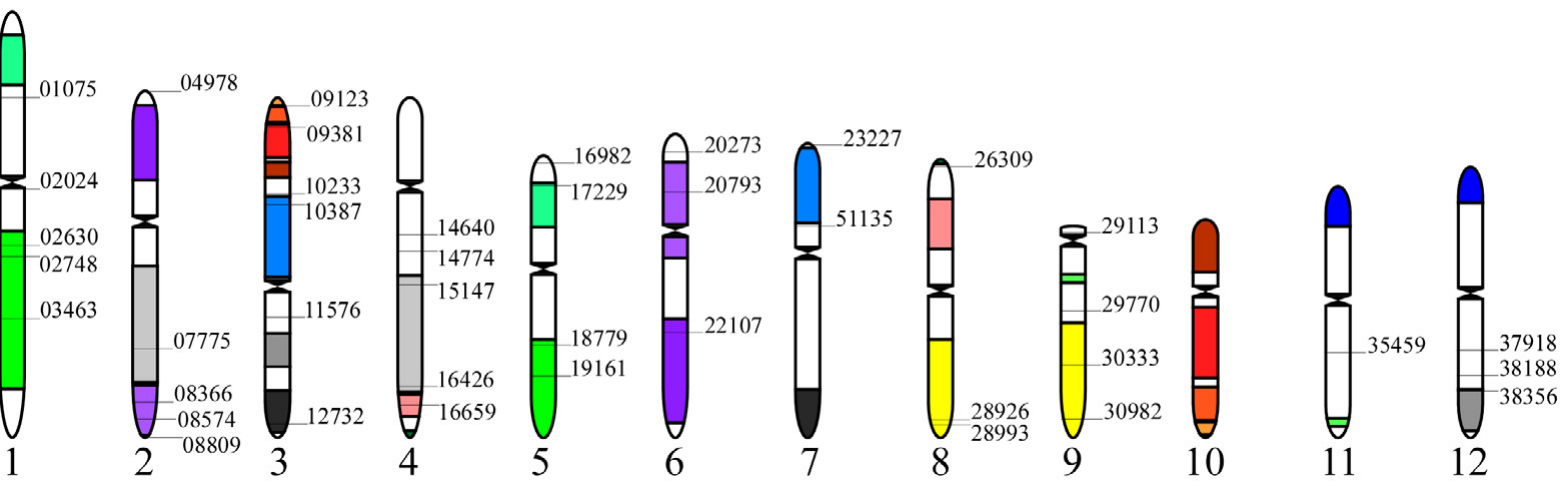
**Duplication map for *O. sativa ssp. japonica *kinesins**. Figure showing the approximated distribution of *Oryza *kinesins on a chromosomal duplication map based upon [94]. All accession numbers should be prefixed with "OSBCCO" if searching [100].

Whole genome duplications are believed to be a driving force for genome evolution in angiosperms since many modern diploids appear to be ancient polyploids [90,91]. In Arabidopsis, the duplicated segments represent about 58% of the genome and several kinesins are present in the duplicated regions [43,92]. In *Oryza*, 18 pairs of duplicated segments cover 65.7% of the mapped super-scaffolds [93]. To visually represent the number of *O. sativa ssp. japonica *kinesins that fall within these segmental duplicated regions, an approximated chromosome map was generated according to the genomic map presented by Guyot and Keller, [94]. Figure 15 depicts the distribution of kinesins across the 12 *Oryza *chromosomes. Roughly 26 of the 41 *japonica *kinesins are within duplicated segments. Chromosome 3 has the most kinesin genes (6), whereas chromosome 10 has none. The remaining chromosomes contain one or more kinesin encoding genes. The duplicated region in the long arm of chromosome 1 contains three kinesins (OSBCC02630, OSBCC02748, OSBCC03463); the corresponding duplicated block on chromosome 5 has only two (OSBCC18779, OSBCC19161), which could be suggestive of a gene loss event. The duplicated block on the short arm of chromosome 2 also may have experienced a gene loss event as it is bereft of any kinesins, whereas its counterpart on the long arm of chromosome 6 contains a single (OSBCC22107) kinesin.

### Intron/exon organization of kinesin genes

Information on the presence of introns in kinesins of all species analyzed here is presented in Tables 2 to 7 and Additional files 1 to 12. All kinesins in *Chlamydomonas *and flowering plants have many introns whereas introns are absent in kinesin genes of red alga, *Giardia *and *Leishmania*. Because of the large number of introns in kinesin genes of most species, the diversity of kinesin motors may increase by alternative splicing of kinesin pre-mRNAs. Although the extent of alternative splicing of kinesin pre-mRNAs in plants is not known, there are examples in animals where alternative splicing of some kinesins results in generation of isoforms with different domains and with distinct functions [95,96].

## Conclusion

Flowering plants have the largest number of kinesins among all species yet sequenced. Gene duplication and functional diversification of specific families (e.g., Kinesin-14 and Kinesin-7) appears to have contributed to the high number of kinesins in flowering plants. Addition of novel domains to kinesins in lineage-specific groups contributed partly to the functional diversification of kinesins. The Kinesin-14 family, which typically contains a C-terminal motor, has many plant kinesins that have the motor domain in the middle or at the N terminus as well as at the C terminus. The presence of most kinesin families of flowering plants in *Chlamydomonas *indicates that these families were retained in both lineages. Since plants have no or few dyneins, it appears that the kinesin family of MT motors has expanded in plants. Despite the large number of kinesins in flowering plants, three or four of the 14 recognized families are absent. The vast expansion of some kinesin families in flowering plants suggests that they are likely to perform plant-specific functions. Many kinesins in *Leishamania*, *Giardia *and *Chlamydomonas *were not resolved with known kinesin families and may represent novel kinesin families and/or early-derived members of the 14 recognized kinesin families that are not resolved as such in our inferred gene tree. Lineage-specific domain architecture in the plant and opisthokont lineages and absence of these domains in kinesins of other eukaryotes suggests acquisition of these domains more recently. The gene-tree analysis presented here is important for understanding kinesin evolution and should provide a framework to study cellular roles of kinesins. The challenge ahead is to elucidate the functions of individual kinesins and their regulation.

## Methods

### Identification and analyses of kinesins in recently completed genome sequences

All BLAST searches were conducted by using three distinct motor domain sequences from the Kinesin-1 (human KHC, N-terminal motor), Kinesin-13 (mouse KIF2, internal motor domain) and Kinesin-14 (AtKCBP, C-terminal motor domain) families. Unless otherwise noted, BLAST searches were done using all three motor domains as queries. In all BLASTP searches we used an E value cut off of 1. With this cut off value, all database searches yielded kinesins and many unrelated proteins. We then performed domain analysis on all hits as described below in section II. All proteins with kinesin motor domain are retained whereas the rest are eliminated.

#### i) Oryza sativa *ssp*. japonica

All of the available genome sequences of this subspecies were extensively analyzed for kinesins as described below.

##### a) Searches at NCBI and Bioverse

BLASTP searches at [97] using the "Oryza sativa" (ssp. *japonica*) protein database were performed. Sequences with each motor-domain search were concatenated into a single file and duplicates were removed. The same query sequences were used in BLASTP searches against the nr database at NCBI [98]. After the output files were parsed and compared with the original PlantBlast searches, a preliminary total of 41 kinesins were identified. To identify the kinesins that may not have been annotated in the genome using the gene-prediction programs, TBLASTN searches were performed against the PlantBlast "Oryza sativa" DNA sequence database. This search resulted in identification of one new kinesin that was not part of the preliminary total obtained via BLASTP searches.

Analysis of Bioverse *Oryza *database at [99] using the keyword, "kinesin" yielded 72 hits. The amino acid sequences from these 72 hits were extracted for BLASTP searches against the nr and PlantBlast databases at NCBI and against each other. This analysis resulted in identification of two new additional kinesins (bringing the total to 44), whereas the rest corresponded either to previous kinesin predictions or were not kinesins.

##### b) Searches at *Oryza *genome databases

The protein predictions for ssp. *japonica *were downloaded from rice genome database [100]. Recently, the analysis of two subspecies of *Oryza *(*indica *and *japonica*) was refined [93]. The Syngenta predictions were refined by using a new program, BGF (Beijing Gene Finding), developed by the Gene Finding Team at BGI for gene identification in eukaryotic genomic DNA sequences. It is based on Dynamic Programming and HSMM (Hidden Semi-Markov Model) algorithm with a special emphasis on *Oryza *genomes [101]. BLASTP searches were performed. The output files were parsed, concatenated and duplicates were removed. Fifty-five possible kinesins were found and their full-length amino acid sequences were retrieved. FGENESH (another gene finding program) predictions were also downloaded from [100] and used for BLAST searches. The predicted kinesins here were blasted against the BGF Syngenta predictions and no new kinesins were found. The kinesins obtained from NCBI were used in a BLASTP search against the BGF Syngenta predictions. All sequences from NCBI were accounted for by the BGF Syngenta sequences. Closer inspection of the output file revealed that two submissions made by independent investigators to GenBank, AAF78897 (817aa) and CAE05519 (1094aa) are duplicates, with AAF78897 being a truncated version of CAE05519. AAF78897 was eliminated from the original NCBI list because the CAE05519 sequence is similar to the BGF predicted OSSBC014640 (1109aa) in sequence length. Also predictions XP_450031.1 and XP_450032.1 are duplicates. XP_450032.1 (971aa) is the truncated version of XP_450031.1 (1035aa), which better corresponds to OSSBCC029113 (1045aa). Hence, XP_450032.1 was eliminated. XP_483647.1 and XP_483646.1 (986aa and 1003aa, respectively) are also duplicates, but we eliminated XP_483646.1 from the NCBI list because the 986 amino acids of XP_483647.1 were a better match with OSSBC028926 (965aa). Consequently, theoriginal list of 44 NCBI kinesins has been decreased to 41. This analysis showed that all 41 kinesin sequences derived from NCBI searches were referenced to the BGF Syngenta *predicted japonica *sequences. Therefore, BGF Syngenta kinesins were used in order to foster continuity for gene-tree analyses. Analysis of all 55 BGF kinesins using Interproscan [102] resulted in (see Section II) a total of 41 kinesins.

#### ii) Oryza sativa *ssp*. indica

For the subspecies *indica *cv. *93-11*, BGF and FGENESH protein databases were downloaded from the ftp site at [100]. BLASTP searches were performed as above using similar criteria. After parsing the output files, the 54 FGENESH predictions were reciprocally blasted against the 55 BGF *indica *sequences and it was found that all FGENESH predictions were included in the BGF predictions. Analysis of these sequences using Interproscan as described in Section II yielded a total of forty-five *indica *kinesins.

#### iii) Arabidopsis thaliana

*A. thaliana *accession numbers for kinesins were obtained [43] and used to retrieve the full-length sequences of all 61 kinesins from TAIR [103].

#### iv) Populus trichocarpa

A predicted protein database is not yet available for *P. trichocarpa*. This database presented a unique problem as the gene/protein predictions were done using four (FGENESH, EUGENE, GRAIL, GENEWISE) eukaryotic gene-prediction programs. Protein predictions at [104] from each program were searched using the keyword, "kinesin". This analysis yielded 115 putative kinesins (36 FGENESH, 50 EUGENE, 5 GRAIL/GENWISE, 24 EST_EXT FGENESH). Duplicate removal and domain analyses produced a set of 52 unique sequences (see Section II).

#### v) Cyanidioschyzon merolae

The C. *merolae *annotated coding sequences and translated ORF databases at [105,106] were used for BLASTP searches. This search yielded five kinesins, which were extracted using the search function at [106] and inputting the locus accessions.

#### vi) Chlamydomonas reinhardtii

BLASTP searches were performed against the Version 2 protein models database at [104]. The hits were parsed of duplicates and 35 sequences were extracted from the database. This analysis yielded 23 kinesins (see Section II).

#### vii) Thalassiosira pseudonana

*T. pseudonana *release 1.0 predicted proteins database was downloaded from JGI and used for BLASTP searches. Twenty-seven kinesins were extracted, but only 22 were used in our gene tree analyses (see Section II).

#### viii) Ciona intestinalis

*C. intestinalis *release 1.0 predicted-proteins database was downloaded from JGI and analyzed in the manner described above. Thirty-five potential kinesins were found, but the last hit was only 93 amino acids long and was discarded.

#### ix) Phanerochaete chryosporium

Due to the lack of a predicted protein database for *P. chryosporium*, potential kinesin sequences were acquired by means of advanced-keyword searches at JGI with the query, "kinesin". Eleven hits were found and downloaded in FASTA format but only eight were used in gene-tree analyses (see Section II).

#### x) Phytophthora sojae

The *P. sojae *predicted protein database was downloaded from [104] and used for BLASTP searches. Interproscan analysis of 56 hits led to 43 possible kinesins.

#### xi) Giardia lamblia

A translated ORF database for *G. lamblia *was downloaded from [107]. BLASTP searches of this database returned 24 hits. These sequences were extracted from the database and analyzed using Interproscan.

#### xii) Homo sapiens

The IDs of 36 kinesins obtained from the Kinesin HomePage [108] were used to acquire the protein sequences using batch entrez at NCBI [109]. Sequences were run through Interproscan for domain analysis and only 32 sequences were kept (see Section II).

Since this number is smaller than what was found in previously published studies, the CELERA protein database was downloaded from [110] and used for BLASTP searches. Forty-four putative kinesins were obtained and analyzed by Interproscan.

Sequences harboring motor domains that were less than 290 amino acids were discarded. The remaining 26 sequences were blasted against the 32 NCBI kinesins to remove duplicates. Eight unique CELERA sequences were appended to the 32 NCBI sequences for a working total of 40 human kinesins.

#### xiii) Drosophila melanogaster

BLASTP searches using HsKHC at [111] were performed. Sequences were downloaded and reciprocally blasted against each other. Five sequences (CG1453-PA, -PB, -PC, -PD and -PE) are replicates, thus only CG1453-PA was retained for analysis. Likewise, 8183-A and 8183-B are duplicates and 8183-A was kept. Also CG9913-A and B are duplicates and only 9913-A was kept. After Interproscan analysis 25 possible kinesins were found.

#### xiv) Caenorhabditis elegans

BLASTP searches were performed at [112]. Nineteen sequences were retrieved and run through Interproscan. Sequence F22F4.3, which has a short motor domain (248 amino acids) was removed and corresponding GI|7499692 from NCBI, which has a longer predicted protein was used instead.

#### xv) Dictyostelium discoideum

Protein sequences were downloaded from [113] and used for BLASTP searches. Thirteen possible kinesin hits were retrieved and analyzed by Interproscan.

#### xvi) Plasmodium falciparum

*P. falciparum-*predicted protein databases (Pfa3D7_WholeGenome_Annotated_PEP_2004.11.23 and Pfa3D7_WholeGenome_Automatic_PEP_2004.11.23) were downloaded from [114] and used in BLASTP searches. Nine sequences were found from the annotated PEP database, whereas 25 sequences were recovered from the automated PEP database.

The 25 automated PEP sequences were extracted and reciprocally blasted to eliminate duplicates. A cross blast was performed between the two databases that resulted in 9 kinesins.

#### xvii) Leishmania major

The *L. major *amino acid database was downloaded from [115] and used for BLASTP searches. Fifty-five sequences were retrieved and reciprocally blasted to search for duplicates. The final number of kinesins in this species is 54 after one duplicate was removed.

#### xviii) Saccharomyces cerevisiae

BLASTP searches at [116] recovered six kinesins.

#### xix) Schizosaccharomyces pombe

BLASTP searches at [117] resulted in nine kinesins.

Additional searches of six frame translations of the genome sequences have not yielded any new kinesins.

### Analysis of domains and retrieval of motor domain sequences

#### a) Interproscan analyses

Interproscan [118] was downloaded to perform batch analyses of the full-length sequences of the putative kinesins from all species. Start and end positions of motor domains were obtained by SMART predictions. The 55 full-length *BGF-japonica *sequences were scanned; inspection of *O*sSBCC020712 (*indica *ortholog, *Os*IBCD019766), a 192 amino acid protein provided the impetus for establishing the criteria necessary to generate a working list of kinesins for gene-tree analyses. Global alignments of all *japonica *kinesins to AtKCBP were examined and any sequences missing conserved domains such as ATP binding sites or with motor domains that were generally shorter than 290 amino acids were discarded. Adherence to these criteria reduced the 54 potential *japonica *kinesins to 41. Following these criteria, ssp. *indica *sequences were reduced to 45. The 115 *P. trichocarpa *hits (FGENESH, EUGENE, EST_EXT FGENESH) were scanned and filtered of any sequences that did not contain motor domains. Of these, 52 hits (21 FGENESH, 21 EUGENE, 10 EST_EXT FGENESH) contained the motor domain. The remaining sequences that did not contain the motor domain were eliminated as well as those that contained truncated motor domains (FGENESHl_pg.C_scaffold_70000181 and FGENESHl_pg.C_LG_I000890). Thirty-five *C. reinhardtii *sequences were scanned and all sequences with motor domains shorter than 290 amino acids were discarded to yield 23. Some *T. pseudonana *proteins were extremely short and consisted only of truncated motor domains. Consequently, the working number of kinesins was lowered to 22. Thirty-four *C. intestinalis *sequences were scanned and the number of kinesins was reduced to 29 due to truncated motor domains or very short sequences.

Scans of the 11 *P. chryosporium *sequences reduced the number of kinesins in this species to eight. *Homo sapiens *sequences GI6225915 and GI3978240 are duplicates. Sequence GI19923949 with a motor domain (232aa) was excluded. Twenty-six *D. melanogaster *kinesins were scanned and only 25 were truly predicted kinesins. The 55 *L. major *sequences were scanned and one sequence (LmjF25.2410) was removed because it had no motor domain.

#### b) Extraction of motor domain

Motor-domain sequences were extracted from their entire protein sequences by using the EMBOSS seqret utility [119] with base ranges obtained from SMART predictions. Concatenation of all motor domain files together yielded a final number of 529 kinesin sequences for gene-tree analysis. All coiled-coil predictions were found by utilization of SMART predictions at [83]. A total of 529 sequences were included in the analysis. Ten sequences had one or two ambiguous amino acids, for a total of 12. Prior to alignment, all 12 internal stop codons (from eight sequences with one to three internal stops each) were changed to amino acid ambiguities ("X").

### Gene-tree construction

Alignment of amino acid kinesin-motor-domain sequences was performed using DIALIGN-T 0.1.2 [53] with the default settings (length of a low-scoring region = 4; maximum fragment length that is allowed to contain regions of low quality = 40). Amino acids from individual sequences that DIALIGN did not align (5,126) were replaced with ambiguities. The DIALIGN output file was 6,046 positions long. Of these, 2,584 positions included aligned amino acid(s) and 834 (32%) of those positions were parsimony-informative.

SeqState 1.2 [120] was use to implement simple indel coding [121] for gaps that were flanked by aligned residues at both the amino and carboxy termini. Of the 1,190 non-terminal gap characters, 668 were parsimony-informative. Fifty-one percent of the cells were either missing data or inapplicables for the parsimony-informative gap characters, as were 60% of the cells for the parsimony-informative amino acid characters. To examine the effect of incorporating gap characters into the study, gene-tree analyses were performed both with and without the gap characters. Gene-tree analyses were performed using amino acid characters. Although amino acid characters have problems with convergence [122,123] and composite coding [124], that nucleotide characters are not subject to, they are expected to perform relatively better when high genetic distances occur among closely related terminals included in the analysis [125], as is the case here. This expectation is based on silent substitutions undergoing saturation (i.e., multiple hits along individual branches). Gene-tree inference was performed using both parsimony and Bayesian MCMC [126] approaches. Parsimony tree searches were performed using PAUP* 4.0b10 [127] with all characters assigned equal weights. Jackknife analyses were performed using 1,000 replicates with each replicate consisting of one tree-bisection-reconnection heuristic search and only one tree held. Following Farris *et al*., [128], the deletion probability for each character was set at 36.7879% and "Jac" resampling was emulated, resulting in support values roughly equivalent to those provided by the bootstrap [129].

Bayesian tree searches were performed using MrBayes 3.1 [130] with a mixed amino acid model. All analyses were performed with four chains per analysis and trees sampled every 100 generations. A preliminary analysis of over 3.6 million generations for the amino-acid-characters-only data matrix was performed using MrBayes 3.0b4. To speed convergence in the final analyses, the MAP tree topology found in this preliminary analysis was specified as the initial user tree with 20 permutations [131].

For the amino-acid-characters-only data matrix, three independent runs were performed for between 1,001,000 and 1,628,500 generations each. All three runs asymptotically approached the same stationarity within the first 200,000 generations, and the remaining 30,919 trees were used to infer the posterior probabilities for individual clades. The analysis that reached 1,628,500 generations was performed in parallel on two dual-processor 1.8 GHz Power Mac G5 computers and ran for approximately 6 weeks. The other two independent runs were executed for seven weeks on a cluster of dual-processor 1 GHz IBM PCs. The data matrix that included gap characters was analyzed using a mixed model with the binary Felsenstein (1981)-type model applied to the gap-characters partition with the ascertainment bias set to variable, as suggested by Ronquist *et al*., [131]. The no-common-mechanism model [132] was not applied to the gap characters because MrBayes 3.1 crashed when applying this model together with a parametric model in a mixed-model analysis. Seven independent runs were performed for between 272,500 and 1,118,100 generations each. All seven runs asymptotically approached the same stationarity, six of which did so within the first 150,000 generations, and the seventh within the first 450,000 generations because of a late stepwise increase in likelihoods. The remaining 28,000 trees were used to infer the posterior probabilities for individual clades. The analysis that reached 1,118,100 generations was executed in parallel for approximately five weeks on two dual processor 1.8 GHz Power Mac G5 computers, whereas the other six independent runs were executed for seven weeks on the previously mentioned cluster.

Majority-rule consensus trees for the Bayesian analyses were calculated using PAUP*. Note that although Bayesian analyses appear to be more efficient than parsimony analyses [133], they also can produce inflated support values [134-136]. Also of concern for Bayesian analyses of these data matrices, which include high proportions of cells with missing data and inapplicables, is that smaller clades may receive high support despite ambiguous resolution of "wildcard" terminals [137]. This may account, in part, for the greater resolution in the Bayesian trees (439 and 453 clades resolved in the amino-acid-only and amino-acid-plus-gap-character analyses, respectively) than in the parsimony trees (288 and 296 clades resolved) given the many completely unresolved terminals in both the Bayesian (36 and 20 terminals) and parsimony (107 and 112 terminals) trees.

The rooting of the kinesin family used by Goodson *et al*., [138], Kim and Endow [139], and Reddy and Day [43] is arbitrary. Outgroup sequences are to be selected such that all members of the ingroup are more closely related to one another than any one of them is to the outgroups (i.e., the ingroup should be monophyletic relative to the outgroup; [140]. Although ScSMY1 is "a highly divergent kinesin protein" [139], this does not satisfy the criterion of selecting an outgroup. See Lawrence *et al*., [42] for an alternative rooting, wherein ScSMY1 is nested within the Kinesin-I family. Following Hirokawa [3], Miki *et al*., [6], Iwabe and Miyata [52], Schoch *et al*., [141] and Abdel-Ghany *et al*., [142], our gene trees are presented as unrooted. All gene trees (except Fig. 2) were drawn using a combination of automatic and manual methods. The use of TreeGraph 1.0b8 [143] greatly facilitated the making of complex tree figures. Though TreeGraph allows one to specify node labels, ours were inputted manually using an external drawing program. TreeGraph can be downloaded from [144].

### Analysis of gene structure and expression data

Gene-structure information was obtained by performing searches with gene identifiers at the appropriate web pages. For *G. lamblia*, the complete contig assembly was downloaded and used as a database for BLASTN searches using the 24 *G. lamblia *sequences in nucleotide format (obtained from [107]). All 24 sequences returned contig hits that were 100% identical with no gaps, indicating that there are no introns in *G. lamblia *kinesins. For the eight human Celera sequences, a Celera transcript database was downloaded and searched with the Celera protein IDs for corresponding transcripts that have been annotated with exon number.

Expression data were collected by performing BLAST searches against appropriate databases (*O. sativa *ssp. *japonica*, *H. sapiens *EST database, *L. major *EST database). A full sequence file containing *japonica *cDNAs was downloaded from [145] and used for BLASTP searches using the 41 BGF Syngenta kinesins as queries. No cDNA data were available for *indica *sequences. For the NCBI human kinesins, EST data were determined by performing a TBLASTN search against the human EST database using the 32 NCBI sequences. *Leishmania major *expression data were obtained by performing a TBLASTN search using the 54 full-length sequences against a database of 2,184 EST sequences. There were no hits found.

### Mapping of kinesins in *O. sativa *ssp. *japonica *chromosomes

Chromosomal duplications in ssp. *japonica *on the genomic map were based on Guyot and Keller [94]. Chromosomes were rescaled appropriately and kinesins were mapped according to base pair positions obtained from [100].

## Authors' contributions

ASNR conceived of the study and coordinated the work. ASNR, DNR and MPS participated in the design of the study. DNR performed all database searches, acquired the sequence data and prepared all figures and tables. MPS and DNR performed the alignments and phylogenetic analyses. ASNR, DNR and MPS participated in data analysis and interpretation, and writing of the manuscript. All authors read and approved the final manuscript.

## Supplementary Material

Additional file 1 **Supplemental Table 1**. *D. melanogaster *kinesins and their structural features. Click here for file

Additional file 12 **Supplemental Table 12**. *G. lamblia *kinesins and their structural features. Click here for file

Additional file 13 **Supplemental Fig 13**. DIALIGN-T alignment of kinesin motor domains. Click here for file

Additional file 14 **Supplemental Fig 14**. Data matrix for parsimony analysis of kinesin motor domains using both amino acid and gap characters. Click here for file

Additional file 17 **Supplemental Fig 17**. Data matrix for Bayesian analysis of kinesin motor domains using both amino acid and gap characters. Click here for file

Additional file 18 **Supplemental Fig 18**. Unrooted parsimony jackknife tree inferred only from amino acid characters. Click here for file

Additional file 20 **Supplemental Fig 20**. Unrooted Bayesian tree with posterior probabilities inferred only from amino acid characters. Click here for file

Additional file 2 **Supplemental Table 2**. *H. sapiens *kinesins and their structural features. Click here for file

Additional file 3 **Supplemental Table 3**. *C. elegans *kinesins and their structural features. Click here for file

Additional file 4 **Supplemental Table 4**. *S. cerevisiae *kinesins their structural features. Click here for file

Additional file 5 **Supplemental Table 5**. *S. pombe *kinesins and their structural features. Click here for file

Additional file 6 **Supplemental Table 6**. *P. chryosporium *kinesins and their structural features. Click here for file

Additional file 7 **Supplemental Table 7**. *P. sojae *kinesins and their structural features. Click here for file

Additional file 8 **Supplemental Table 8**. *C. intestinalis *kinesins and their structural features. Click here for file

Additional file 9 **Supplemental Table 9**. *P. falciparum *kinesins and their structural features. Click here for file

Additional file 10 **Supplemental Table 10**. *D. discoideum *kinesins and their structural features. Click here for file

Additional file 11 **Supplemental Table 11**. *L. major *kinesins and their structural features. Click here for file

Additional file 15 **Supplemental Fig 15**. Data matrix for Bayesian analysis of kinesin motor domains using only amino acid characters. Click here for file

Additional file 16 **Supplemental Fig 16**. Data matrix for Bayesian analysis of kinesin motor domains using only amino acid characters. Click here for file

Additional file 19 **Supplemental Fig 19**. Unrooted parsimony jackknife tree inferred from both amino acid and gap characters. Click here for file

## Figures and Tables

**Table 1:** Number of kinesins in the completed genomes of 19 Eukaryotes

**Species**	*#*	**Reference**	**Databases used in our analyses**
*C. merolae*	*5*	Analyzed here	[106]
*C. reinhardtii*^*a*^	23	Analyzed here	[104]
*A. thaliana*	61	[43]	[103]
*P. trichocarpa*^*a*^	52	Analyzed here	[104]
*O.s japonica *cv. *nipponbare*^*a*^	41	Analyzed here	[97, 100]
*O.s indica *cv. *9311*^*a*^	45	Analyzed here	[100]
*T. pseudonana*^*a*^	22	Analyzed here	[104]
*D. melanogaster*	25	[146]	[111]
*H. sapiens*^*b*^	40	[6]	[98, 110]
*C. elegans*	19	[147]	[112]
*S. cerevisiae*	6	Yeastgenome.org	[116]
*S. pombe*	9	*pombe *GeneDB	[117]
*P. chryosporium*	8	Analyzed here	[104]
*P. sojae*^*a*^	43	Analyzed here	[104]
*C. intestinalis*^*a*^	25	Analyzed here	[104]
*P. falciparum*	9	Analyzed here	[114]
*D. discoideum*	13	[148] Analyzed here	[113]
*L. major*	54	Analyzed here	[115]
*G. lamblia*	24	Analyzed here	[107]

Table 1 represents individual number of kinesins found in 19 eukaryotic species. Five of the 529 sequences are from Mouse and are not shown here. Thirteen of these species were analyzed in this paper. ^a^There are potentially more kinesins than listed; see individual tables for more information. ^b^There may be up to 45 potential kinesins as reported. Only 44 were found in our database searches, 4 of which contained incomplete motor domains and were not listed in the total.

**Table 2:** *C. merolae *kinesins and their structural features

**Gene ID**	**Protein length**	**EST**	**Additional Domains**	**MD location**	**# of exons**	**Family**
CMC157C	916	Yes		N	1	5
CMQ429C	1175	Yes	CC	N	1	7
CMO070C	1290	No	CC	N	1	12
CMT097C	1160	Yes	CC, LH2	I	1	14
CMR497C	851	Yes	CC	C	1	14

CC, Coiled-coil; LH2, Lipoxygenase; N, N-terminal; I, Internal; C, C-terminal.

**Table 3:** *C. reinhardtii *kinesins and their structural features

**Gene ID**	**Protein length**	**EST**	**Additional Domains**	**MD location**	**# of exons**	**Family**
C_160226	768	No	CC	N	12	2
C_1880008	786	Yes	CC	N	21	2
C_120157	1917	No	CC	N	23	4
C_260133	1008	Yes	CC	N	23	4
C_340020	1510	No	CC, N- Syn	N	20	4
C_530027	1115	No	CC	N	13	5
C_1480016	1520	No	CC	N	18	7
C_710026	825	No	CC	N	24	7
C_1310024	935	No	CC	N	19	8
C_250150	961	No	CC	N	17	9
C_50080	776	No	CC	N	12	9
C_260066	967	No	CC	N	9	9
C_620070	610	No	CC	N	11	9
C_1350007	1248	No	CC	N	19	12
C_730040	647	No	CC	I	8	13
C_510041	968	No	CC	I	14	14
C_670066	855	No	CC	C	17	14
C_1030008	1136	Yes	MyTH4, B4.1, CC	C	18	14
C_60218	837	No	CC	C	18	14
C_120136	918	No	CC	C	18	14
C_460075	1152	No	CC	N	15	UG
C_790050	1484	No	CC	N	24	UG
C_570090	1366	No	CC	N	12	UG

Other potential kinesins that had either truncated motor domains or unfinished sequences are: C_60056, C_200191, C_130219, C_230013, C_300121, C_70192, C_990021, C_7460001, C_770005, C_930065. MyTH4, Myosin tail homology 4; B4.1, Band 4.1; CC, Coiled-coil; N-Syn, N terminal syntaxin; UG, Ungrouped; N, N-terminal; I, Internal; C, C-terminal.

**Table 4:** *P. trichocarpa *kinesins and their structural features

**Gene ID**	**Protein length**	**EST**	**Additional Domains**	**MD location**	**# of exons**	**Family**
LG_VIII000260	429	Yes		I	10	UG
LG_II000635	921	No	CC, ARM	N	19	UG
LG_III0949	943	Yes	CC, ARM	N	20	UG
LG_II000522	970	No	CC, VWA	N	23	4
LG_VII000272	1036	Yes	CC	N	25	4
00020976^a^	1290	Yes	CC	N	27	4
02310007^a^	1280	No	CC	N	27	4
00160756^a^	1031	No	CC	N	22	5
LG_I001798	1077	No	CC	N	22	5
00060694^a^	1049	No	CC	N	23	5
LG_XVI0226	1031	Yes	TM	N	23	5
LG_XIII0655	944	Yes	CC	N	23	6
LG_XII000185	964	Yes	CC	N	13	7
scaffold 28000071	952	No	CC	N	14	7
LG_IX0409	939	Yes	CC	N	14	7
570109^b^	909	Yes		N	17	7
LG_II000243	915	No		N	13	7
00020745^a^	701	No		N	18	7
00160532^a^	939	Yes	CC	N	23	7
LG_IV1283	1139	Yes	CC, ZF	N	25	7
LG_XIV000016	817	Yes	CC	N	19	7
LG_VI0681	1233	Yes	CC	N	27	7
00120495^a^	784	Yes	CC	I	7	8
00151235^a^	656	No	HHH	N	17	10
LG_VIII001511	853	No	CC	N	8	10
LG_X000373	964	Yes	CC	N	6	10
01300020^a^	2921	No	CC	N	35	12
LG_IX000131	1300	No	CC	N	22	12
00012593^a^	1196	No	CC, KR	N	15	12
LG_XIV000891	1278	Yes	CC, KR	N	17	12
LG_II001048	1281	No	CC, KR	N	17	12
00100152^a^	814	Yes	CC	I	12	13
02100029^a^	734	Yes		I	11	13
01330083^a^	1022	No	CC, CH	I	19	14
00140705^a^	957	Yes	CH	I	18	14
00120480^a^	847	No	CC, CH	C	18	14
00150291^a^	1020	No	CC, CH	C	19	14
LG_III001053	1133	No	CC, CH	I	21	14
1400045^b^	1085	Yes	CH	I	16	14
00060440^a^	1079	No	CH	I	17	14
00111158^a^	1129	No	CC	I	16	14
LG_I003040	1212	No	CC	I	16	14
LG_II001016	1046	Yes	CC	I	19	14
00130189^a^	778	No	CC	I	13	14
02560013^a^	767	No	CC	I	12	14
07000001^a^	498	Yes		I	10	14
LG_I000011	947	No	CC	N	11	14
LG_XIII000327	637	No	CC	I	10	14
LG_VII001210	1070	No	CC	N	21	14
LG_XI1218	1232	Yes	MyTH4, B4.1, CC	C	23	14
LG_XI0089	801	Yes	CC	C	16	14
LG_XI000300	752	Yes	CC	C	16	14

^a^For sequence acquisition at JGI, prefix with "eugene3." ^b^For sequence acquisition at JGI, prefix with "estext_fgeneshl_pg_vl.C_". Other possible kinesins with truncated motor domains: fgenesl_pg.C_LG_I000890, 0344001^b^, 00700259^b ^and 08910001^b^. MyTH4, Myosin tail homology 4; B4.1, Band 4.1; CC, Coiled-coil; KR, Kinesin-Related; VWA, Von Willebrand factor type A; CH, Calponin homology; ARM, Armadillo repeat; HHH, Helix-hairpin-helix; ZF, Zing finger; TM, Transmembrane domain; UG, Ungrouped; N, N-terminal; I, Internal; C, C-terminal.

**Table 5:** *O. sativa ssp. japonica *kinesins and their structural features

**Gene ID**	**Protein length**	**cDNA Accession^a^**	**Additional Domains**	**MD location**	**# of exons**	**Family**
OsSBCC009381	909		CC, ARM	N	20	UG
OsSBCC020273	868		CC, ARM	N	19	UG
OsSBCC026309	434		CC	N	15	1
OsSBCC029113	1045	AK067738 AK100974^b^	CC	N	25	4
OsSBCC008366	1260	AK106279	CC, HTH	N	25	4
OsSBCC016982	1027	AK068757	CC	N	21	5
OsSBCC010233	818			N	18	5
OsSBCC028993	988		CC	N	21	5
OsSBCC004978	854	AK102833		N	23	6
OsSBCC002024	954	AK103077	CC	N	14	7
OsSBCC007775	618	AK100979	CC	N	11	7
OsSBCC028926	965	AK065731 AK102208^b^		N	12	7
OsSBCC030982	877	AK072633 AK100918^b^		N	13	7
OsSBCC015147	1116		CC	N	23	7
OsSBCC008574	975	AK121333	CC	N	23	7
OsSBCC035459	642		CC	N	14	7
OsSBCC030333	1368		CC	N	32	7
OsSBCC002630	743		CC	I	8	8
OsSBCC012732	729	AK103310	CC	N	16	8
OsSBCC008809	896		CC	N	6	10
OsSBCC019161	563	AK073413	HHH	N	11	10
OsSBCC014774	955	AK101769	HHH	I	19	10
OsSBCC038188	2798		CC	N	36	12
OsSBCC011576	1221	AK121995	CC, KR	N	16	12
OsSBCC014640	1109		CC, KR	N	16	12
OsSBCC002748	774		CC	I	13	13
OsSBCC017229	819		CC	I	12	13
OsSBCC010387	1051	AK106465	CH	I	16	14
OsSBCC037918	919	AK065586		I	17	14
OsSBCC022107	1169	AK064200	CC	I	20	14
OsSBCC003463	1317	AK101026	CH, CC	I	25	14
OsSBCC038356	1003	AK106725	CC	I	15	14
OsSBCC009123	1080	AK063381	CC	I	21	14
OsSBCC018779	840	AK1 02894	CC	I	13	14
OsSBCC001075	793	AK070313	CC	N	10	14
OsSBCC029770	580	AK071093	CC	N	10	14
OsSBCC020793	1311		CC	N	24	14
OsSBCC016659	1274	AK073209	MyTH4, B4.1, CC	C	23	14
OsSBCC023227	762	AK063949	CC	C	14	14
OsSBCC051135	367	AK063949		N	9	14
OsSBCC016426	788	AK122184 AK064973^b^	CC	C	17	14

^a^Available cDNA accession numbers for KOME database [145]. ^b^Indicates KOME cDNA accessions that may represent alternatively spliced isoforms. Other possible kinesins with truncated motor domains OsSBCC015355, OsSBCC019553, OsSBCC035936, OsSBCC025788, OsSBCC033202, OsSBCC015813, OsSBCC012593, OsSBCC000978, OsSBCC005893, OsSBCC005892, OsSBCC007770, OsSBCC006810, OsSBCC035600. MyTH4, Myosin tail homology 4; B4.1, Band 4.1; CC, Coiled-coil; CH, Calponin homology; HTH, Helix turn helix; HHH, Helix-hairpin-helix; ARM, Armadillo repeat; KR, Kinesin-Related; UG, Ungrouped; N, N-terminal; I, Internal; C, C-terminal.

**Table 6:** *O. sativa ssp. indica *kinesins and their structural features

**Gene ID**	**Protein length**	**Additional Domains**	**MD location**	**# of exons**	**Family**
OsIBCD008900	899	CC, ARM	N	20	UG
OsIBCD019322	891	CC, ARM	N	19	UG
OsIBCD024826	434	CC	N	15	1
OsIBCD027529	1045	CC	N	25	4
OsIBCD007936	1260	CC, HTH	N	25	4
OsIBCD016250	1027	CC	N	21	5
OsIBCD009684	1637	CC, ZF	N	29	5
OsIBCD027399	951	CC	N	20	5
OsIBCD004715	2588	ARM	N	40	6
OsIBCD001985	954	CC	N	14	7
OsIBCD007402	828	CC	N	12	7
OsIBCD027313	947		N	12	7
OsIBCD029182	1102	CC	N	15	7
OsIBCD014393	1140	CC	N	23	7
OsIBCD008139	948	CC	N	23	7
OsIBCD031186	1014	CC	N	23	7
OsIBCD033171	642	CC	N	14	7
OsIBCD028585	1236	CC	N	31	7
OsIBCD002607	768	CC	I	6	8
OsIMBCD012108	783	CC	N	17	8
OsIBCD008347	904	CC	N	6	10
OsIBCD018270	563	HHH	N	11	10
OsIBCD014035	722	HHH	N	18	10
OsIBCD035525	2887	CC	N	40	12
OsIBCD010981	1129	CC, KR	N	15	12
OsIBCD0139 10	1085	CC, KR	N	15	12
OsIBCD016493	811	CC	I	12	13
OsIBCD009838	1007	CH	I	17	14
OsIBCD035259	913		I	18	14
OsIBCD021045	1225	CC, CH	I	20	14
OsIBCD005609	892	CC, CH	C	18	14
OsIBCD003270	938	CC, CH	C	22	14
OsIBCD014642	1014	CC, CH	I	19	14
OsIBCD035672	994	CC	I	15	14
OsIBCD008627	1080	CC	I	21	14
OsIBCD017918	831	CC	I	13	14
OsIBCD001052	793	CC	N	10	14
OsIBCD025572	604	CC	N	10	14
OsIBCD025570	502	CC	N	7	14
OsIBCD019841	1311	CC	N	24	14
OsIBCD015937	1274	MyTH4, B4.1, CC	C	23	14
OsIBCD022126	762	CC	C	14	14
OsIBCD012736	486		I	10	14
OsIBCD031252	622	CC	C	18	14
OsIBCD015695	788	CC	C	17	

Other possible kinesins with truncated motor domains: OsIBCD000942, OsIBCD001990, OsIBCD002704, OsIBCD006479, OsIBCD011989, OsIBCD014306, OsIBCDOlSlll, OsIBCD019933, OsIBCD024322. MyTH4, Myosin tail homology 4; B4.1, Band 4.1; CC, Coiled-coil; CH, Calponin homology; HTH, Helix-turn-helix; ZF, Zinc Finger; HHH, Helix-hairpin-helix; ARM, Armadillo repeat; KR, Kinesin related; UG, Ungrouped; N, N-terminal; I, Internal; C, C-terminal.

**Table 7:** *T. pseudonana *kinesins and their structural features

**Gene ID**	**Protein length**	**EST**	**Additional Domains**	**MD location**	**# of exons**	**Family**
133437	318	No		ND	3	1
128511	330	No		ND	2	1
119229	340	Yes		N	5	4
135880	529	No	CC	N	8	5
137385	404	No		C	4	6
121289	380	No	CC	N	6	7
105812	485	No	CC	N	2	9
142737	454	No	CC	N	3	9
132625	338	No		N	4	12
112325	597	No	CC	I	1	13
116492	532	No		C	4	13
107522	528	No		I	1	13
122963	453	No		N	5	14
123312	365	Yes		C	3	14
12124	362	No		C	4	14
124560	606	No	CC	C	9	14
151754	391	No		C	1	14
152375	328	No		ND	4	14
110182	428	No		N	3	UG
156428	396	No		N	2	UG
163717	305	No		ND	3	UG
155696	757	No	CC	N	6	UG

Possible kinesins with truncated motor domain: 10932, 170556, 159990, 154168, 142297. CC, Coiled-coil; ND, Not determined; UG, Ungrouped; N, N-terminal; I, Internal; C, C-terminal.
