# Supplementary material for: Comprehensive comparative analysis of kinesins in photosynthetic eukaryotes
Source: BMC Genomics. 2006 Jan 31;7:18. doi: 10.1186/1471-2164-7-18 (PMC1434745; doi:10.1186/1471-2164-7-18)
Supplement: Additional file 1 — Supplemental Table 1. D. melanogaster kinesins and their structural features. [file 1471-2164-7-18-S1.pdf]

Supplemental Table 1 - *D. melanogaster* kinesins and their structural features

Gene ID	Protein length	EST	Additional Domains	MD location	# of exons	Family
CG7765-PA	975	Yes	CC	N	4	1
CG17461-PA	574	Yes	CC	N	4	2
CG7293-PA	784	Yes	CC	N	3	2
CG10642-PA	677	No	CC	N	1	2
CG5658-PA	1265	Yes	CC, FHA	N	12	3
CG8566-PD	1670	Yes	CC, FHA, PH	N	26	3
CG8183-PA	1921	Yes	CC, FHA, CAP-Gly	N	18	3
CG10718-PA	1121	Yes	CC, FHA	N	2	3
CG5300-PA	1048	Yes	CC	N	12	4
CG8590-PA	1212	Yes	CC	N	6	4
CG9191-PA	1066	Yes	CC	N	6	5
CG1258-PA	887	Yes	CC	N	6	6
CG12298-PA	628	Yes	CC	I	3	6
CG14535-PA	1131	No		N	6	6
CG32955-PE	1931	Yes	CC	N	18	7
CG6392-PA	2013	No	CC	N	10	7
CG9913-PA	728	No	CC	N	7	8
CG10923-PA	814	Yes	CC	N	5	8
CG15844-PA	844	Yes	GGL	I	9	9
CG12192-PA	729	Yes		I	1	13
CG3219-PA	626	Yes		I	1	13
CG1453-PA	805	Yes	CC	I	9	13
CG7831-PA	700	Yes	CC	C	3	14
CG1708-PA	1201	Yes	CC	N	4	UG
CG1763-PA	666	Yes	HHH	N	7	UG

CC, Coiled-coil; FHA, Fork head associated; PH, Pleckstrin homology; CAP-Gly, Glycine rich domain found in Cytoskeleton Associated Proteins (CAPs); GGL, G-protein gamma subunit like motif; HHH, Helix-hairpin-helix; UG, Ungrouped; N, N-terminal; I, Internal; C, C-terminal.
