# Supplementary material for: Comprehensive comparative analysis of kinesins in photosynthetic eukaryotes
Source: BMC Genomics. 2006 Jan 31;7:18. doi: 10.1186/1471-2164-7-18 (PMC1434745; doi:10.1186/1471-2164-7-18)
Supplement: Additional file 12 — Supplemental Table 12. G. lamblia kinesins and their structural features. [file 1471-2164-7-18-S12.pdf]

Supplemental Table 12 - *G. lamblia* kinesins and their structural features

Gene ID	Protein length	SAGE Tag	Additional Domains	MD location	# of exons	Family
13825	979	Yes	CC, HTH	N	1	1
16456	642	Yes	CC	N	1	2
17333	718	No	CC	N	1	2
102101	1026	Yes		N	1	3
112846	1095	Yes	FHA	N	1	3
6262	1073	Yes	FHA	N	1	3
16650	1056	No	CC	N	1	4
16425	1066	No	CC	N	1	5
7874	777	No	CC	N	1	7
16161	837	No	CC	N	1	7
4371	777	Yes	CC	N	1	8
10137	756	No	CC	N	1	9
6404	767	Yes	CC	N	1	9
16945	714	No		I	1	13
13797	1172	No	CC	C	1	14
8886	625	No	CC	C	1	14
14070	793	Yes		N	1	UG
17264	796	No	CC	N	1	UG
112729	1028	No	CC	N	1	UG
15134	1039	Yes		N	1	UG
102455	1194	Yes	CC	N	1	UG
16224	905	Yes		N	1	UG
11442	639	No	CC	N	1	UG
15962	923	Yes	CC	N	1	UG

CC, Coiled-coil; FHA, Fork head associated; HTH, Helix-turn-helix; UG, Ungrouped; N, N-terminal; I, Internal; C, C-terminal.
