# Supplementary material for: Comprehensive comparative analysis of kinesins in photosynthetic eukaryotes
Source: BMC Genomics. 2006 Jan 31;7:18. doi: 10.1186/1471-2164-7-18 (PMC1434745; doi:10.1186/1471-2164-7-18)
Supplement: Additional file 18 — Supplemental Fig 18. Unrooted parsimony jackknife tree inferred only from amino acid characters. [file 1471-2164-7-18-S18.pdf]

Supplemental Figure 18. Unrooted parsimony jackknife tree inferred only from amino acid characters

```
Parsimony motor domain amino acid characters only 50% Majority-rule
consensus of 1000 trees (all trees equally weighted)

/-------------------------------------------------------- AT 1G01950(1)
|
|                                                  /----- AT 1G09170(2)
|                                        /---52----+
|                                        |         \----- AT 5G27000(51)
|                                        |
|                                        |         /----- AT 2G47500(20)
|                                        |    /-84-+
|                                        |    |    \----- PT 00140705(404)
|                                        +-82-+
|                                        |    |    /----- OS SBCC010387(307)
|                        /------58-------+    \100-+
|                        |               |         \----- OS IBCD009838(348)
|                        |               |
|                        |               |         /----- OS SBCC037918(331)
|                        |               +---100---+
|                        |               |         \----- OS IBCD035259(377)
|                        |               |
|                        |               \--------------- PT 01330083(410)
|                        |
|                        |                         /----- AT 1G18410(5)
|                        |                    /100-+
|                        |                    |    \----- AT 1G73860(13)
|                        |               /-69-+
|                        |               |    |    /----- PT 00120480(401)
|                        |               |    \100-+
|                        |               |         \----- PT 00150291(405)
|                   /-71-+               |
|                   |    |               |         /----- AT 1G63640(11)
|                   |    |               |    /100-+
|                   |    |          /-68-+    |    \----- AT 5G41310(54)
|                   |    |          |    +-77-+
|                   |    |          |    |    \---------- PT LG III001053(420)
|                   |    |          |    |
|                   |    |          |    |         /----- OS SBCC022107(321)
|                   |    |          |    |    /100-+
|                   |    |     /-94-+    |    |    \----- OS IBCD021045(364)
|                   |    |     |    |    \-99-+
|                   |    |     |    |         \---------- OS IBCD005609(340)
|              /-99-+    |     |    |
|              |    |    |     |    |              /----- OS SBCC003463(298)
|              |    |    |     |    \-----100------+
|              |    |    \-75--+                   \----- OS IBCD003270(338)
|              |    |          |
|              |    |          |                   /----- AT 3G44730(32)
|              |    |          |              /-99-+
|              |    |          |              |    \----- PT 1400045(384)
|         /-87-+    |          \------95------+
|         |    |    |                         \---------- OS IBCD014642(355)
|         |    |    |
|         |    |    |                              /----- AT 3G10310(22)
|         |    |    \-------------100--------------+
|         |    |                                   \----- PT 00060440(397)
|         |    |
|         |    \----------------------------------------- CR C 510041(128)
|         |
|         |                                        /----- AT 1G18370(4)
|         |                                   /100-+
|         |                                   |    \----- PT LG XII000185(434)
|         |                              /-91-+
|         |                              |    |    /----- OS SBCC002024(295)
|         |                              |    \100-+
|         |                    /---81----+         \----- OS IBCD001985(336)
|         |                    |         |
|         |                    |         |         /----- AT 3G43210(30)
|         |                    |         \---55----+
|         |                    |                   \----- PT SCAFFOLD 2800(428)
|         |                    |
|         |                    |                   /----- AT 2G21300(14)
|         |                    |              /100-+
|         |                    |              |    \----- AT 4G38950(45)
|         |                    |         /100-+
|         |                    |         |    \---------- PT LG IX0409(388)
|         |                    |    /-71-+
|         +--------100---------+    |    |         /----- OS SBCC007775(300)
|         |                    |    |    \---100---+
|         |                    |    |              \----- OS IBCD007402(341)
|         |                    |    |
|         |                    |    |              /----- AT 3G51150(36)
|         |                    |    |         /100-+
|         |                    |    |         |    \----- AT 5G66310(61)
|         |                    |    +---96----+
|         |                    |    |         \---------- PT 570109(385)
```

```
|         |                   |    |
|         |                   |    |              /----- AT 4G24170(43)
|         |                   \100-+-----100------+
|         |                        |              \----- AT 5G42490(55)
|         |                        |
|         |                        |              /----- OS SBCC028926(324)
|         |                        |         /100-+
|         |                        |         |    \----- OS IBCD027313(369)
|         |                        +---98----+
|         |                        |         |    /----- OS SBCC030982(329)
|         |                        |         \100-+
|         |                        |              \----- OS IBCD029182(373)
|         |                        |
|         |                        \-------------------- PT LG II000243(418)
|         |
|         |                                       /----- AT 1G18550(6)
|         |                                  /-75-+
|         |                                  |    \----- PT 00120495(402)
|         +----------------100----------------+
|         |                                  |    /----- OS SBCC002630(296)
|         |                                  \100-+
|         |                                       \----- OS IBCD002607(337)
|         |
|         |                                       /----- AT 1G20060(7)
|         |                                  /-70-+
|         |                                  |    \----- PT LG XIII0655(392)
|         +----------------100----------------+
|         |                                  |    /----- OS SBCC004978(299)
|         |                                  \100-+
|         |                                       \----- OS IBCD004715(339)
|         |
|         |                             /--------------- AT 1G21730(8)
|         |                             |
|         |                             |         /----- OS SBCC015147(312)
|         |                        /-69-+    /100-+
|         |                        |    |    |    \----- OS IBCD014393(354)
|         |                        |    \-51-+
|         |                        |         \---------- PT 00020745(395)
|         |                        |
|         |                   /-92-+              /----- AT 3G12020(23)
|         |                   |    |         /-83-+
|         |                   |    |         |    \----- AT 5G06670(48)
|         |                   |    |    /-94-+
|         |                   |    |    |    \---------- PT 00160532(407)
|         |                   |    \-92-+
|         |                   |         |         /----- OS SBCC008574(302)
|         |              /-100-+        \---99----+
|         |              |    |                   \----- OS IBCD008139(343)
|         |              |    |
|         |              |    |                   /----- AT 2G21380(15)
|         |              |    |              /-67-+
|         |              |    |              |    \----- AT 4G39050(46)
|         +------55------+    \-----100------+
|         |              |                   |    /----- OS IBCD031186(374)
|         |              |                   \-55-+
|         |              |                        \----- PT LG IV1283(387)
|         |              |
|         |              \------------------------------ DD B0189194(65)
|         |
|         |                                       /----- AT 1G55550(9)
|         |                                  /100-+
|         |                                  |    \----- PT LG I000011(415)
|         |                        /---97----+
|         |                        |         |    /----- OS SBCC001075(294)
|         |                        |         \100-+
|         |                        |              \----- OS IBCD001052(335)
|         |                        |
|         +-----------99------------+              /----- AT 5G27950(53)
|         |                        |    /---100---+
|         |                        |    |         \----- PT LG XIII000327(426)
|         |                        |    |
|         |                        \100-+         /----- OS SBCC029770(327)
|         |                             |    /-93-+
|         |                             |    |    \----- OS IBCD025572(368)
|         |                             \100-+
|         |                                  \---------- OS IBCD025570(367)
|         |
|         |                                       /----- AT 1G59540(10)
|         |                                  /-93-+
|         |                                  |    \----- PT LG XIV000016(435)
|         |                             /100-+
|         |                             |    |    /----- OS SBCC035459(330)
|         |                             |    \100-+
|         |                             |         \----- OS IBCD033171(376)
|         |                        /-95-+
|         |                        |    |         /----- AT 3G10180(21)
|         |                        |    |    /100-+
|         |                        |    |    |    \----- PT LG VI0681(389)
|         |                        |    \100-+
|         +-----------50------------+         |    /----- OS SBCC030333(328)
|         |                        |         \100-+
|         |                        |              \----- OS IBCD028585(372)
|         |                        |
|         |                        \-------------------- DD B0186344(63)
|         |
```

```
|         |                              /--------------- AT 1G72250(12)
|         |                              |
|         |                              |         /----- OS SBCC038356(333)
|         |                         /-99-+    /100-+
|         |                         |    |    |    \----- OS IBCD035672(379)
|         |                         |    \-51-+
|         |                         |         |    /----- PT 00111158(400)
|         |                         |         \100-+
|         |                    /-89-+              \----- PT LG I003040(417)
|         |                    |    |
|         |                    |    |              /----- AT 2G22610(16)
|         |                    |    |         /-74-+
|         |                    |    |         |    \----- PT LG II001016(419)
|         |                    |    \---100---+
|         |                    |              |    /----- OS SBCC009123(304)
|         |                    |              \100-+
|         +--------100---------+                   \----- OS IBCD008627(345)
|         |                    |
|         |                    |         /--------------- AT 5G27550(52)
|         |                    |         |
|         |                    |    /-81-+    /---------- PT 00130189(403)
|         |                    |    |    |    |
|         |                    |    |    \100-+    /----- PT 02560013(413)
|         |                    |    |         \100-+
|         |                    \-98-+              \----- PT 07000001(414)
|         |                         |
|         |                         |              /----- OS SBCC018779(317)
|         |                         \-----100------+
|         |                                        \----- OS IBCD017918(360)
|         |
|         |                                        /----- AT 2G28620(17)
|         |                                        |
|         |                                        +----- AT 3G45850(33)
|         |                                   /-73-+
|         |                                   |    +----- PT 00160756(408)
|         |                                   |    |
|         |                         /---80----+    \----- PT LG I001798(416)
|         |                         |         |
|         |                         |         |    /----- OS SBCC016982(315)
|         |                         |         \100-+
|         |                         |              \----- OS IBCD016250(358)
|         |                    /-54-+
|         |                    |    |    /--------------- AT 2G37420(19)
|         |                    |    |    |
|         |                    |    |    |         /----- OS SBCC010233(306)
|         |                    |    \-85-+    /100-+
|         |                    |         |    |    \----- OS IBCD009684(347)
|         |              /-81--+         \-71-+
|         |              |     |              \---------- PT 00060694(398)
|         |              |     |
|         |              |     |                   /----- AT 2G36200(18)
|         |              |     |              /-91-+
|         |              |     |              |    \----- PT LG XVI0226(393)
|         |         /100-+     \-----100------+
|         |         |    |                    |    /----- OS SBCC028993(325)
|         |         |    |                    \100-+
|         |         |    |                         \----- OS IBCD027399(370)
|         |         |    |
|         |         |    \------------------------------- CR C 530027(129)
|         |         |
|         |         +------------------------------------ DD B0187903(64)
|         |         |
|         |         +------------------------------------ TP 135880(87)
|         |         |
|         |         |                              /----- CE F23B12.8(99)
|         |         |                              |
|         |         +--------------68--------------+----- DM CG9191 PA(209)
|         |         |                              |
|         |         |                              \----- HS 1706622(224)
|         +---72----+
|         |         +------------------------------------ GL 16425(148)
|         |         |
|         |         +------------------------------------ PS 108445(252)
|         |         |
|         |         +------------------------------------ CM C157C(490)
|         |         |
|         |         |                              /----- SC CIN8(495)
|         |         |                         /-72-+
|         |         |                         |    \----- SC KIP1(497)
|         |         |                         |
|         |         +-----------87------------+---------- PC PC.41.67.1(507)
|         |         |                         |
|         |         |                         \---------- SP AC25G10.07C(520)
|         |         |
|         |         \------------------------------------ PF C0770C(513)
|         |
|         |                                        /----- AT 3G16060(24)
|         |                         /------97------+
|         |                         |              \----- OS SBCC002748(297)
|         |                         |
|         |                         |         /---------- AT 3G16630(25)
|         |                    /100-+         |
|         |                    |    |    /-83-+    /----- PT 00100152(399)
|         |                    |    |    |    \-72-+
|         |                    |    |    |         \----- PT 02100029(411)
```

```
|         |                    |    \-99-+
|         |                    |         |         /----- OS SBCC017229(316)
|         |                    |         \---100---+
|         |                    |                   \----- OS IBCD016493(359)
|         |                    |
|         |                    +------------------------- DD B0216601(71)
|         |                    |
|         |                    +------------------------- TP 107522(76)
|         |                    |
|         |                    |                   /----- TP 112325(78)
|         |                    +--------95---------+
|         |                    |                   \----- PS 136489(269)
|         |                    |
|         |                    +------------------------- TP 116492(79)
|         |                    |
|         |                    |    /-------------------- CE K11D9.1A(100)
|         |                    |    |
|         |                    |    |         /---------- CI 0100146176(182)
|         |                    |    |         |
|         |                    |    |    /-60-+    /----- HS 3024057(229)
|         |                    +-98-+    |    \-95-+
|         |                    |    |    |         \----- HS 49355831(243)
|         |                    |    |    |
|         |                    |    |    |         /----- DM CG12192 PA(189)
|         |                    |    \-56-+    /-81-+
|         |                    |         |    |    \----- DM CG3219 PA(198)
|         +---------54---------+         +-98-+
|         |                    |         |    \---------- DM CG1453 PA(192)
|         |                    |         |
|         |                    |         \--------------- HS 1695882(223)
|         |                    |
|         |                    +------------------------- CR C 730040(135)
|         |                    |
|         |                    +------------------------- GL 16945(151)
|         |                    |
|         |                    +------------------------- PS 135741(267)
|         |                    |
|         |                    +------------------------- PS 136872(270)
|         |                    |
|         |                    +------------------------- PS 142524(285)
|         |                    |
|         |                    |                   /----- LM F01.0030(436)
|         |                    +--------67---------+
|         |                    |                   \----- LM F13.1610(446)
|         |                    |
|         |                    +------------------------- LM F13.0130(444)
|         |                    |
|         |                    +------------------------- LM F24.0640(468)
|         |                    |
|         |                    +------------------------- LM F31.0290(479)
|         |                    |
|         |                    +------------------------- LM F35.4700(488)
|         |                    |
|         |                    +------------------------- PC PC.13.63.1(505)
|         |                    |
|         |                    \------------------------- PF L2165W(516)
|         |
|         |                              /--------------- AT 3G17360(26)
|         |                              |
|         |                              |         /----- AT 3G19050(27)
|         |                         /-95-+    /-96-+
|         |                         |    |    |    \----- PT 01300020(409)
|         |                         |    \-89-+
|         |                         |         |    /----- OS SBCC038188(332)
|         |                    /100-+         \100-+
|         |                    |    |              \----- OS IBCD035525(378)
|         |                    |    |
|         |                    |    |              /----- AT 3G44050(31)
|         +---------52---------+    \-----100------+
|         |                    |                   \----- PT LG IX000131(421)
|         |                    |
|         |                    \------------------------- CR C 1350007(118)
|         |
|         |                                        /----- AT 3G20150(28)
|         |                                   /-64-+
|         |                                   |    \----- PT 00012593(394)
|         |                         /---100---+
|         |                         |         |    /----- OS SBCC011576(308)
|         |                         |         \100-+
|         |                         |              \----- OS IBCD010981(349)
|         |                         |
|         |                         |              /----- AT 3G23670(29)
|         +-----------99------------+         /100-+
|         |                         |         |    \----- AT 4G14150(40)
|         |                         |    /100-+
|         |                         |    |    |    /----- PT LG XIV000891(427)
|         |                         |    |    \-99-+
|         |                         \100-+         \----- PT LG II001048(431)
|         |                              |
|         |                              |         /----- OS SBCC014640(310)
|         |                              \---100---+
|         |                                        \----- OS IBCD013910(352)
|         |
|         |                                   /---------- AT 3G49650(34)
|         |                                   |
```

```
|         |                              /100-+    /----- OS SBCC012732(309)
|         |                              |    \100-+
|         +-------------100--------------+         \----- OS IBCD012108(350)
|         |                              |
|         |                              \--------------- CR C 1310024(117)
|         |
|         |                         /-------------------- AT 3G50240(35)
|         |                         |
|         |                         |         /---------- AT 5G47820(56)
|         |                         |         |
|         |                    /100-+         |    /----- OS SBCC029113(326)
|         |                    |    |    /-65-+100-+
|         |                    |    |    |    |    \----- OS IBCD027529(371)
|         |                    |    |    |    |
|         |                    |    \-64-+    \---------- PT LG II000522(429)
|         |                    |         |
|         |                    |         \--------------- PT LG VII000272(432)
|         |              /-97--+
|         |              |     |              /---------- AT 5G60930(58)
|         |              |     |              |
|         |              |     |         /-96-+    /----- PT 00020976(396)
|         |              |     |         |    \100-+
|         |              |     |         |         \----- PT 02310007(412)
|         +------83------+     \---100---+
|         |              |               |         /----- OS SBCC008366(301)
|         |              |               \---100---+
|         |              |                         \----- OS IBCD007936(342)
|         |              |
|         |              \------------------------------- CR C 120157(116)
|         |
|         |                                   /---------- AT 3G63480(38)
|         |                                   |
|         +----------------100----------------+    /----- OS SBCC026309(323)
|         |                                   \100-+
|         |                                        \----- OS IBCD024826(366)
|         |
|         |                                        /----- AT 4G05190(39)
|         |                                   /-99-+
|         |                                   |    \----- AT 4G21270(42)
|         |                         /---81----+
|         |                         |         \---------- PT LG XI0089(390)
|         |                         |
|         |                         |              /----- AT 4G27180(44)
|         |                         |         /100-+
|         |                         |         |    \----- AT 5G54670(57)
|         |                         +---77----+
|         |                         |         \---------- PT LG XI000300(433)
|         |                         |
|         |                         |              /----- OS SBCC016426(313)
|         |                    /-98-+-----100------+
|         |                    |    |              \----- OS IBCD015695(356)
|         |                    |    |
|         |                    |    |              /----- OS SBCC023227(322)
|         |                    |    |         /-90-+
|         |                    |    |         |    \----- OS IBCD022126(365)
|         |                    |    |    /-74-+
|         +---------57---------+    |    |    |    /----- OS SBSC051135(334)
|         |                    |    |    |    \100-+
|         |                    |    \100-+         \----- OS IBCD012736(351)
|         |                    |         |
|         |                    |         \--------------- OS IBCD031252(375)
|         |                    |
|         |                    \------------------------- CR C 60218(131)
|         |
|         |                                   /---------- AT 4G14330(41)
|         |                                   |
|         |                              /-91-+    /----- PT LG VIII001511(424)
|         |                              |    \100-+
|         |                              |         \----- PT LG X000373(425)
|         +-------------100--------------+
|         |                              |         /----- OS SBCC008809(303)
|         |                              \---100---+
|         |                                        \----- OS IBCD008347(344)
|         |
|         |                                   /---------- AT 5G02370(47)
|         |                                   |
|         |                              /100-+    /----- OS SBCC019161(318)
|         |                              |    \100-+
|         |                              |         \----- OS IBCD018270(361)
|         |                              |
|         +--------------96--------------+         /----- AT 5G23910(50)
|         |                              |    /-92-+
|         |                              |    |    \----- PT 00151235(406)
|         |                              \-88-+
|         |                                   |    /----- OS SBCC014774(311)
|         |                                   \100-+
|         |                                        \----- OS IBCD014035(353)
|         |
|         |                                        /----- AT 5G10470(49)
|         |                                   /100-+
|         |                                   |    \----- AT 5G65460(59)
|         |                              /-79-+
|         |                              |    \---------- PT LG VII001210(422)
|         +-------------100--------------+
|         |                              |         /----- OS SBCC020793(320)
```

```
|         |                              \---100---+
|         |                                        \----- OS IBCD019841(363)
|         |
|         |                                   /---------- AT 5G65930(60)
|         |                                   |
|         |                              /-51-+    /----- OS SBCC016659(314)
|         |                              |    \100-+
|         |                         /100-+         \----- OS IBCD015937(357)
|         |                         |    |
|         |                    /-98-+    \--------------- PT LG XI1218(391)
|         |                    |    |
|         |                    |    \-------------------- CR C 1030008(114)
|         |                    |
|         +---------96---------+------------------------- TP 12124(81)
|         |                    |
|         |                    +------------------------- CI 0100131201(166)
|         |                    |
|         |                    \------------------------- PS 141193(281)
|         |
|         +---------------------------------------------- DD B0166988(62)
|         |
|         |                    /------------------------- DD B0189215(66)
|         |                    |
|         |                    |              /---------- CE R144.1(105)
|         |                    |              |
|         +---------62---------+         /-81-+    /----- CI 0100136694(175)
|         |                    |         |    \-82-+
|         |                    |    /-98-+         \----- CI 0100137520(176)
|         |                    |    |    |
|         |                    \-93-+    \--------------- CI 0100134202(173)
|         |                         |
|         |                         \-------------------- PS 108377(251)
|         |
|         +---------------------------------------------- DD B0189377(67)
|         |
|         +---------------------------------------------- DD B0189854(68)
|         |
|         +---------------------------------------------- DD B0204045(69)
|         |
|         +---------------------------------------------- DD B0204609(70)
|         |
|         +---------------------------------------------- DD B0217523(72)
|         |
|         |                                        /----- DD B0218612(73)
|         +-------------------50-------------------+
|         |                                        \----- PS 142668(287)
|         |
|         +---------------------------------------------- DD B0219839(74)
|         |
|         +---------------------------------------------- TP 105812(75)
|         |
|         |                                        /----- TP 110182(77)
|         +------------------100-------------------+
|         |                                        \----- TP 156428(92)
|         |
|         +---------------------------------------------- TP 119229(80)
|         |
|         |                                        /----- TP 121289(82)
|         +-------------------99-------------------+
|         |                                        \----- TP 163717(93)
|         |
|         |                                        /----- TP 122963(83)
|         +-------------------58-------------------+
|         |                                        \----- PS 142636(286)
|         |
|         +---------------------------------------------- TP 123312(84)
|         |
|         |                                   /---------- TP 124560(85)
|         |                                   |
|         |                              /-54-+    /----- TP 151754(90)
|         |                              |    \-64-+
|         |                         /-89-+         \----- PS 124499(291)
|         |                         |    |
|         +-----------61------------+    \--------------- PS 129079(260)
|         |                         |
|         |                         \-------------------- CR C 120136(115)
|         |
|         +---------------------------------------------- TP 132625(86)
|         |
|         +---------------------------------------------- TP 137385(88)
|    /100-+
|    |    |                                   /---------- TP 142737(89)
|    |    |                                   |
|    |    +----------------68-----------------+    /----- CR C 250150(122)
|    |    |                                   \-51-+
|    |    |                                        \----- PS 137042(272)
|    |    |
|    |    +---------------------------------------------- TP 155696(91)
|    |    |
|    |    |                                        /----- TP 133437(94)
|    |    +-------------------52-------------------+
|    |    |                                        \----- PS 137039(271)
|    |    |
|    |    +---------------------------------------------- CE 01083(95)
|    |    |
```

```
|    |    |                                       /----- CE C06G3.2(96)
|    |    +------------------100------------------+
|    |    |                                       \----- CE C33H5.4A(97)
|    |    |
|    |    |                                       /----- CE C41G7.2(98)
|    |    |                                  /100-+
|    |    |                                  |    \----- CE M01E11.6(102)
|    |    +----------------100---------------+
|    |    |                                  \---------- CE W02B12.7(108)
|    |    |
|    |    |                                  /---------- CE M02B7.3A(101)
|    |    |                                  |
|    |    |                             /-75-+    /----- CI 0100134965(174)
|    |    |                             |    \-55-+
|    |    |                        /-75-+         \----- HS HCP48743(217)
|    |    |                        |    |
|    |    |                        |    \--------------- DM CG17461 PA(196)
|    |    |                        |
|    |    |                        |    /--------------- CE F20C5.2B(111)
|    |    |                        |    |
|    |    |                        +-77-+    /---------- CI 0100133723(172)
|    |    |                        |    |    |
|    |    |                        |    \-76-+    /----- HS 3913957(233)
|    |    |                        |         \-66-+
|    |    |                        |              \----- HS 3913958(234)
|    |    |                        |
|    |    |                        +-------------------- CR C 160226(120)
|    |    |                        |
|    |    |                        +-------------------- CR C 1880008(121)
|    |    +-----------81-----------+
|    |    |                        |              /----- GL 16456(149)
|    |    |                        +------89------+
|    |    |                        |              \----- GL 17333(153)
|    |    |                        |
|    |    |                        |              /----- CI 0100148992(183)
|    |    |                        |         /-99-+
|    |    |                        |         |    \----- HS 3851492(232)
|    |    |                        +---97----+
|    |    |                        |         \---------- DM CG10642 PA(186)
|    |    |                        |
|    |    |                        +-------------------- DM CG7293 PA(203)
|    |    |                        |
|    |    |                        |              /----- PS 108563(253)
|    |    |                        \-----100------+
|    |    |                                       \----- PS 120306(255)
|    |    |
|    |    |                        /-------------------- CE M03D4.1A(103)
|    |    |                        |
|    |    |                        |         /---------- CI 0100154383(185)
|    |    +-----------100----------+         |
|    |    |                        |    /-85-+    /----- HS HCP49543(218)
|    |    |                        |    |    \100-+
|    |    |                        \-91-+         \----- HS 400264(238)
|    |    |                             |
|    |    |                             \--------------- DM CG1258 PA(191)
|    |    |
|    |    |              /------------------------------ CE R05D3.7(104)
|    |    |              |
|    |    |              |              /--------------- CI 0100143504(178)
|    |    |              |              |
|    |    |              |              |         /----- HS 2497520(226)
|    |    +-----100------+         /-92-+    /-97-+
|    |    |              |         |    |    |    \----- HS 304358(230)
|    |    |              |         |    \-59-+
|    |    |              |    /-86-+         \---------- HS 417216(240)
|    |    |              |    |    |
|    |    |              \-83--+    \-------------------- SPU 47550911(527)
|    |    |                   |
|    |    |                   \------------------------- DM CG7765 PA(204)
|    |    |
|    |    |                                  /---------- CE T01G1.1A(106)
|    |    |                                  |
|    |    |                                  +---------- CI 0100130413(164)
|    |    |                                  |
|    |    +----------------95----------------+---------- DM CG5300 PA(200)
|    |    |                                  |
|    |    |                                  |    /----- HS HCP1631302(211)
|    |    |                                  \-87-+
|    |    |                                       \----- HS 5360129(244)
|    |    |
|    |    |                        /-------------------- CE UNC 104(107)
|    |    |                        |
|    |    +-----------100----------+    /--------------- CI 0100131206(167)
|    |    |                        |    |
|    |    |                        \-54-+    /---------- DM CG8566 PD(207)
|    |    |                             |    |
|    |    |                             \-73-+    /----- HS 2497523(227)
|    |    |                                  \-80-+
|    |    |                                       \----- HS 3913961(235)
|    |    |
|    |    +--------------------------------------------- CE Y43F4B.6(109)
|    |    |
|    |    |                   /------------------------- CE F56E3.3(110)
|    |    |                   |
|    |    |                   |         /--------------- CI 0100143686(179)
```

```
|    |    |                    |         |
|    |    +---------97---------+         |         /----- HS HCP34632(212)
|    |    |                    |    /-67-+    /100-+
|    |    |                    |    |    |    |    \----- HS 8896164(249)
|    |    |                    |    |    \-80-+
|    |    |                    \-71-+         \---------- HS 11761611(220)
|    |    |                         |
|    |    |                         \-------------------- DM CG8183 PA(206)
|    |    |
|    |    +---------------------------------------------- CE 7499692(112)
|    |    |
|    |    |                         /-------------------- CE K12F2.2A(113)
|    |    |                         |
|    |    +-----------95------------+    /--------------- DM CG14535 PA(193)
|    |    |                         |    |
|    |    |                         \-89-+    /---------- HS 29732867(228)
|    |    |                              |    |
|    |    |                              \100-+    /----- HS 6330751(246)
|    |    |                                   \100-+
|    |    |                                        \----- MM 51767056(382)
|    |    |
|    |    +---------------------------------------------- CR C 1480016(119)
|    |    |
|    |    |                                        /----- CR C 260066(123)
|    |    +-------------------82-------------------+
|    |    |                                        \----- LM F06.0180(439)
|    |    |
|    |    +---------------------------------------------- CR C 260133(124)
|    |    |
|    |    +---------------------------------------------- CR C 340020(125)
|    |    |
|    |    +---------------------------------------------- CR C 460075(126)
|    |    |
|    |    |                              /--------------- CR C 50080(127)
|    |    |                              |
|    |    |                              |    /---------- CI 0100145013(180)
|    |    |                              |    |
|    |    |                              +-77-+    /----- HS 11275982(219)
|    |    +--------------64--------------+    \100-+
|    |    |                              |         \----- MM 5295882(383)
|    |    |                              |
|    |    |                              +--------------- PS 130522(263)
|    |    |                              |
|    |    |                              \--------------- PS 139555(277)
|    |    |
|    |    +---------------------------------------------- CR C 570090(130)
|    |    |
|    |    |                                        /----- CR C 620070(132)
|    |    +-------------------81-------------------+
|    |    |                                        \----- LM F34.1540(485)
|    |    |
|    |    +---------------------------------------------- CR C 670066(133)
|    |    |
|    |    |                                        /----- CR C 710026(134)
|    |    |                                   /-72-+
|    |    |                                   |    \----- PS 128334(258)
|    |    +----------------100----------------+
|    |    |                                   \---------- LM F25.1970(471)
|    |    |
|    |    +---------------------------------------------- CR C 790050(136)
|    |    |
|    |    +---------------------------------------------- GL 7874(137)
|    |    |
|    |    +---------------------------------------------- GL 8886(138)
|    |    |
|    |    +---------------------------------------------- GL 10137(139)
|    |    |
|    |    +---------------------------------------------- GL 11442(140)
|    |    |
|    |    +---------------------------------------------- GL 13797(141)
|    |    |
|    |    +---------------------------------------------- GL 13825(142)
|    |    |
|    |    +---------------------------------------------- GL 14070(143)
|    |    |
|    |    +---------------------------------------------- GL 15134(144)
|    |    |
|    |    +---------------------------------------------- GL 15962(145)
|    |    |
|    |    +---------------------------------------------- GL 16161(146)
|    |    |
|    |    +---------------------------------------------- GL 16224(147)
|    |    |
|    |    +---------------------------------------------- GL 16650(150)
|    |    |
|    |    +---------------------------------------------- GL 17264(152)
|    |    |
|    |    +---------------------------------------------- GL 4371(154)
|    |    |
|    |    |                                        /----- GL 102101(155)
|    |    |                                   /100-+
|    |    |                                   |    \----- GL 112846(160)
|    |    +----------------100----------------+
|    |    |                                   \---------- GL 6262(157)
|    |    |
```

```
|    |    +---------------------------------------------- GL 102455(156)
|    |    |
|    |    +---------------------------------------------- GL 6404(158)
|    |    |
|    |    +---------------------------------------------- GL 112729(159)
|    |    |
|    |    |                                        /----- CI 0100130156(161)
|    |    |                                   /-88-+
|    |    |                                   |    \----- HS 452517(242)
|    |    +----------------80-----------------+
|    |    |                                   \---------- DM CG10718 PA(187)
|    |    |
|    |    |                                        /----- CI 0100130230(162)
|    |    |                                        |
|    |    +-------------------85-------------------+----- HS 3978240(236)
|    |    |                                        |
|    |    |                                        \----- HS 5911999(245)
|    |    |
|    |    |                                   /---------- CI 0100130295(163)
|    |    |                                   |
|    |    +----------------85-----------------+    /----- HS 9910266(250)
|    |    |                                   \100-+
|    |    |                                        \----- MM 40644653(381)
|    |    |
|    |    |                                        /----- CI 0100131135(165)
|    |    |                                   /-73-+
|    |    |                                   |    \----- DM CG15844 PA(194)
|    |    +----------------85-----------------+
|    |    |                                   \---------- HS HCP45833(216)
|    |    |
|    |    |                                        /----- CI 0100131275(168)
|    |    +-------------------59-------------------+
|    |    |                                        \----- HS 7266951(248)
|    |    |
|    |    +---------------------------------------------- CI 0100131488(169)
|    |    |
|    |    +---------------------------------------------- CI 0100133346(170)
|    |    |
|    |    +---------------------------------------------- CI 0100133471(171)
|    |    |
|    |    |                                   /---------- CI 0100141905(177)
|    |    |                                   |
|    |    +----------------100----------------+    /----- HS 4519443(241)
|    |    |                                   \100-+
|    |    |                                        \----- MM 21704182(380)
+-98-+    |
|    |    +---------------------------------------------- CI 0100145354(181)
|    |    |
|    |    |                                        /----- CI 0100151891(184)
|    |    +-------------------88-------------------+
|    |    |                                        \----- HS 4115553(239)
|    |    |
|    |    |                                   /---------- DM CG10923 PA(188)
|    |    |                                   |
|    |    +----------------94-----------------+    /----- HS HCP43728(214)
|    |    |                                   \-85-+
|    |    |                                        \----- HS 12053149(221)
|    |    |
|    |    +---------------------------------------------- DM CG12298 PA(190)
|    |    |
|    |    +---------------------------------------------- DM CG1708 PA(195)
|    |    |
|    |    +---------------------------------------------- DM CG1763 PA(197)
|    |    |
|    |    |                                        /----- DM CG32955 PE(199)
|    |    +------------------100-------------------+
|    |    |                                        \----- DM CG6392 PA(202)
|    |    |
|    |    |                                        /----- DM CG5658 PA(201)
|    |    +-------------------99-------------------+
|    |    |                                        \----- HS 6522736(247)
|    |    |
|    |    +---------------------------------------------- DM CG7831 PA(205)
|    |    |
|    |    +---------------------------------------------- DM CG8590 PA(208)
|    |    |
|    |    +---------------------------------------------- DM CG9913 PA(210)
|    |    |
|    |    +---------------------------------------------- HS HCP41593(213)
|    |    |
|    |    +---------------------------------------------- HS HCP44045(215)
|    |    |
|    |    +---------------------------------------------- HS 12654739(222)
|    |    |
|    |    +---------------------------------------------- HS 23397458(225)
|    |    |
|    |    +---------------------------------------------- HS 3702453(231)
|    |    |
|    |    +---------------------------------------------- HS 399227(237)
|    |    |
|    |    +---------------------------------------------- PS 109123(254)
|    |    |
|    |    |                                        /----- PS 121412(256)
|    |    +-------------------65-------------------+
|    |    |                                        \----- LM F19.0260(459)
```

```
|    |    |
|    |    +----------------------------------------------- PS 127973(257)
|    |    |
|    |    +----------------------------------------------- PS 128382(259)
|    |    |
|    |    +----------------------------------------------- PS 130051(261)
|    |    |
|    |    +----------------------------------------------- PS 130250(262)
|    |    |
|    |    +----------------------------------------------- PS 131621(264)
|    |    |
|    |    +----------------------------------------------- PS 132151(265)
|    |    |
|    |    +----------------------------------------------- PS 134623(266)
|    |    |
|    |    +----------------------------------------------- PS 136418(268)
|    |    |
|    |    |                                         /----- PS 137485(273)
|    |    +-------------------71--------------------+
|    |    |                                         \----- PS 142481(284)
|    |    |
|    |    +----------------------------------------------- PS 137622(274)
|    |    |
|    |    +----------------------------------------------- PS 137761(275)
|    |    |
|    |    +----------------------------------------------- PS 138580(276)
|    |    |
|    |    +----------------------------------------------- PS 140636(278)
|    |    |
|    |    +----------------------------------------------- PS 140964(279)
|    |    |
|    |    |                                         /----- PS 141078(280)
|    |    +-------------------67--------------------+
|    |    |                                         \----- PS 142793(288)
|    |    |
|    |    |                                         /----- PS 141849(282)
|    |    +-------------------56--------------------+
|    |    |                                         \----- PS 143005(290)
|    |    |
|    |    +----------------------------------------------- PS 141959(283)
|    |    |
|    |    +----------------------------------------------- PS 142980(289)
|    |    |
|    |    +----------------------------------------------- PS 121577(292)
|    |    |
|    |    +----------------------------------------------- PS 121181(293)
|    |    |
|    |    +----------------------------------------------- LM F05.0630(437)
|    |    |
|    |    +----------------------------------------------- LM F05.0760(438)
|    |    |
|    |    +----------------------------------------------- LM F06.1030(440)
|    |    |
|    |    +----------------------------------------------- LM F09.0120(441)
|    |    |
|    |    +----------------------------------------------- LM F09.0290(442)
|    |    |
|    |    +----------------------------------------------- LM F11.0870(443)
|    |    |
|    |    +----------------------------------------------- LM F13.0700(445)
|    |    |
|    |    +----------------------------------------------- LM F14.0810(447)
|    |    |
|    |    |                                         /----- LM F14.1100(448)
|    |    |                                    /-64-+
|    |    |                                    |    \----- LM F14.1120(450)
|    |    +----------------100-----------------+
|    |    |                                    \---------- LM F14.1110(449)
|    |    |
|    |    |                                         /----- LM F16.1460(451)
|    |    +------------------100--------------------+
|    |    |                                         \----- LM F16.1470(452)
|    |    |
|    |    |                                         /----- LM F16.1580(453)
|    |    +-------------------93--------------------+
|    |    |                                         \----- LM F35.2090(487)
|    |    |
|    |    +----------------------------------------------- LM F17.0160(454)
|    |    |
|    |    |                                         /----- LM F17.0800(455)
|    |    +-------------------99--------------------+
|    |    |                                         \----- LM F32.0680(482)
|    |    |
|    |    +----------------------------------------------- LM F17.1110(456)
|    |    |
|    |    +----------------------------------------------- LM F18.1530(457)
|    |    |
|    |    +----------------------------------------------- LM F18.1600(458)
|    |    |
|    |    |                                         /----- LM F19.0680(460)
|    |    |                                    /100-+
|    |    |                                    |    \----- LM F19.0690(461)
|    |    +----------------88------------------+
|    |    |                                    \---------- LM F19.0700(462)
|    |    |
```

```
|    |    +---------------------------------------------- LM F20.0640(463)
|    |    |
|    |    +---------------------------------------------- LM F21.1040(464)
|    |    |
|    |    +---------------------------------------------- LM F22.0560(465)
|    |    |
|    |    +---------------------------------------------- LM F22.0960(466)
|    |    |
|    |    +---------------------------------------------- LM F23.0560(467)
|    |    |
|    |    +---------------------------------------------- LM F24.1430(469)
|    |    |
|    |    +---------------------------------------------- LM F25.1950(470)
|    |    |
|    |    +---------------------------------------------- LM F28.1850(472)
|    |    |
|    |    +---------------------------------------------- LM F29.0970(473)
|    |    |
|    |    |                                        /----- LM F29.2380(474)
|    |    +------------------100-------------------+
|    |    |                                        \----- LM F29.2390(475)
|    |    |
|    |    +---------------------------------------------- LM F30.0350(476)
|    |    |
|    |    +---------------------------------------------- LM F30.1450(477)
|    |    |
|    |    +---------------------------------------------- LM F30.3060(478)
|    |    |
|    |    +---------------------------------------------- LM F31.2710(480)
|    |    |
|    |    +---------------------------------------------- LM F32.0420(481)
|    |    |
|    |    +---------------------------------------------- LM F33.2140(483)
|    |    |
|    |    +---------------------------------------------- LM F33.2560(484)
|    |    |
|    |    +---------------------------------------------- LM F34.4260(486)
|    |    |
|    |    +---------------------------------------------- LM F36.5150(489)
|    |    |
|    |    +---------------------------------------------- CM O070C(491)
|    |    |
|    |    +---------------------------------------------- CM Q429C(492)
|    |    |
|    |    +---------------------------------------------- CM R497C(493)
|    |    |
|    |    +---------------------------------------------- CM T097C(494)
|    |    |
|    |    |                                        /----- SC KAR3(496)
|    |    |                                   /-54-+
|    |    |                                   |    \----- SP AC3A11.14C(521)
|    |    |                              /-78-+
|    |    |                              |    \---------- SP AC664.10(522)
|    |    +--------------74--------------+
|    |    |                              \--------------- PC GW.73.22.1(503)
|    |    |
|    |    |                                        /----- SC KIP2(498)
|    |    +-------------------67-------------------+
|    |    |                                        \----- SP BC1604.20C(524)
|    |    |
|    |    |                                        /----- SC KIP3(499)
|    |    |                                   /-74-+
|    |    |                                   |    \----- SP BC1685.15C(525)
|    |    |                              /-54-+
|    |    |                              |    \---------- SP BC2F12.13(526)
|    |    +--------------91--------------+
|    |    |                              \--------------- PC PC.95.48.1(508)
|    |    |
|    |    +---------------------------------------------- SC SMY1(500)
|    |    |
|    |    +---------------------------------------------- PC GW.4.121.1(501)
|    |    |
|    |    |                                        /----- PC GW.41.36.1(502)
|    |    +-------------------52-------------------+
|    |    |                                        \----- SP AC1834.07(519)
|    |    |
|    |    +---------------------------------------------- PC PC.11.256.1(504)
|    |    |
|    |    +---------------------------------------------- PC PC.3.10.1(506)
|    |    |
|    |    +---------------------------------------------- PF MAL8P1.132(509)
|    |    |
|    |    +---------------------------------------------- PF 07 0104(510)
|    |    |
|    |    +---------------------------------------------- PF 11 0478(511)
|    |    |
|    |    +---------------------------------------------- PF A0535C(512)
|    |    |
|    |    +---------------------------------------------- PF C0860W(514)
|    |    |
|    |    +---------------------------------------------- PF L0545W(515)
|    |    |
|    |    +---------------------------------------------- PF L2190C(517)
|    |    |
|    |    +---------------------------------------------- SP AC144.14(518)
```

```
|    |    |
|    |    +---------------------------------------------- SP BC15D4.01C(523)
|    |    |
|    |    +---------------------------------------------- TP 152375(528)
|    |    |
|    |    \---------------------------------------------- TP 128511(529)
|    |
|    |                                             /----- AT 3G54870(37)
|    |                                        /-74-+
|    |                                        |    \----- PT LG VIII000260(423)
|    \-------------------76-------------------+
|                                             |    /----- OS SBCC009381(305)
|                                             \100-+
|                                                  \----- OS IBCD008900(346)
|
|                                                  /----- AT 1G12430(3)
|                                             /-83-+
|                                             |    \----- PT LG III0949(386)
|                                             |
|                                             |    /----- OS SBCC020273(319)
\---------------------57----------------------+100-+
                                              |    \----- OS IBCD019322(362)
                                              |
                                              \---------- PT LG II000635(430)
```
