# Supplementary material for: Comprehensive comparative analysis of kinesins in photosynthetic eukaryotes
Source: BMC Genomics. 2006 Jan 31;7:18. doi: 10.1186/1471-2164-7-18 (PMC1434745; doi:10.1186/1471-2164-7-18)
Supplement: Additional file 20 — Supplemental Fig 20. Unrooted Bayesian tree with posterior probabilities inferred only from amino acid characters. [file 1471-2164-7-18-S20.pdf]

Supplemental Figure 20. Unrooted Bayesian tree with posterior probabilities inferred only from amino acid characters

Bayesian motor AA only 50% Majority-rule consensus of 30919 trees (all trees equally weighted):

```
/--------------------------------------------------------------------------- AT 1G01950(1)
|
|                                                                      /---- AT 1G09170(2)
|                                                                  /--89
|                                                                  |   \---- AT 5G27000(51)
|                                                              /-100
|                                                              |   \-------- PT 01330083(410)
|                                                              |
|                                                         /---97       /---- AT 2G47500(20)
|                                                         |    |   /--99
|                                                         |    |   |   \---- PT 00140705(404)
|                                                         |    \-100
|                                                         |        |   /---- OS SBCC010387(307)
|                                         /-------------100        \-100
|                                         |               |            \---- OS IBCD009838(348)
|                                         |               |
|                                         |               |            /---- OS SBCC037918(331)
|                                         |               \----------100
|                                         |                            \---- OS IBCD035259(377)
|                                         |
|                                         |                            /---- AT 1G18410(5)
|                                         |                        /--53
|                                         |                        |   \---- PT 00151235(406)
|                                         |                    /--82
|                                         |                    |   \-------- AT 1G73860(13)
|                                         |               /---82
|                                         |               |    |       /---- PT 00120480(401)
|                                         |               |    \-----100
|                                         |               |            \---- PT 00150291(405)
|                                     /--86          /--81
|                                     |   |           |   |            /---- OS SBCC022107(321)
|                                     |   |           |   |        /-100
|                                     |   |           |   |        |   \---- OS IBCD021045(364)
|                                     |   |           |   \-------96
|                                     |   |       /--86            \-------- OS IBCD005609(340)
|                                     |   |       |   |
|                                     |   |       |   |                /---- AT 1G63640(11)
|                                     |   |       |   |            /-100
|                                     |   |       |   |            |   \---- AT 5G41310(54)
|                                     |   |   /--87   \----------100
|                                     |   |   |   |                \-------- PT LG III001053(420)
|                                /---87   |   |   |
|                                |    |   |   |   |                    /---- OS SBCC003463(298)
|                                |    |   |   |   \------------------100
|                                |    |   \--87                        \---- OS IBCD003270(338)
|                                |    |       |
|                                |    |       |                        /---- AT 3G44730(32)
|                                |    |       |                    /-100
|                                |    |       |                    |   \---- PT 1400045(384)
|                            /--87    |       \------------------100
|                            |   |    |                            \-------- OS IBCD014642(355)
|                            |   |    |
|                            |   |    |                                /---- AT 3G10310(22)
|                            |   |    \------------------------------100
|                        /--67   |                                    \---- PT 00060440(397)
|                        |   |   |
|                        |   |   \------------------------------------------ CR C 510041(128)
|                        |   |
|                        |   \---------------------------------------------- CM T097C(494)
|                        |
|                        |                                         /-------- AT 1G72250(12)
|                        |                                         |
|                        |                                     /--96   /---- PT 00111158(400)
|                        |                                     |   \-100
|                        |                                     |       \---- PT LG I003040(417)
|                        |                                /--100
|                    /--62                                |    |       /---- OS SBCC038356(333)
|                    |   |                                |    \-----100
|                    |   |                                |            \---- OS IBCD035672(379)
|                    |   |                            /-100
|                    |   |                            |   |            /---- AT 2G22610(16)
|                    |   |                            |   |        /--83
```

```
|                    |    |                             |   |        |   \---- PT LG II001016(419)
|                    |    |                             |   \------100
|                    |    |                             |            |   /---- OS SBCC009123(304)
|                    |    |                             |            \-100
|                    |    |                             |                \---- OS IBCD008627(345)
|                    |    \---------------------------100
|                    |                                  |        /------------ AT 5G27550(52)
|                /--61                                  |        |
|                |   |                                  |   /---81   /-------- PT 00130189(403)
|                |   |                                  |   |    |   |
|                |   |                                  |   |    \-100   /---- PT 02560013(413)
|                |   |                                  |   |        \-100
|                |   |                                  \-100            \---- PT 07000001(414)
|                |   |                                      |
|                |   |                                      |            /---- OS SBCC018779(317)
|                |   |                                      \----------100
|                |   |                                                   \---- OS IBCD017918(360)
|                |   |
|                |   |                                                   /---- TP 122963(83)
|                |   \-------------------------------------------------100
|                |                                                       \---- PS 142636(286)
|                |
|                |                                                       /---- AT 1G55550(9)
|                |                                                   /-100
|                |                                                   |   \---- PT LG I000011(415)
|                |                                          /------100
|                |                                          |        |   /---- OS SBCC001075(294)
|                |                                          |        \-100
|                |                                          |            \---- OS IBCD001052(335)
|                |                                          |
|                |                                      /-100            /---- AT 5G27950(53)
|                |                                      |   |    /-----100
|                |                                      |   |    |       \---- PT LG XIII000327(426)
|            /--74                                      |   |    |
|            |   |                                      |   \--100       /---- OS SBCC029770(327)
|            |   +-------------------------------------53        |   /--98
|            |   |                                      |        |   |   \---- OS IBCD025572(368)
|            |   |                                      |        \-100
|            |   |                                      |            \-------- OS IBCD025570(367)
|            |   |                                      |
|            |   |                                      \--------------------- CE 01083(95)
|            |   |
|            |   |                                                   /-------- TP 123312(84)
|            |   |                                                   |
|            |   |                                               /--99   /---- CI 0100151891(184)
|            |   |                                               |   \-100
|            |   +----------------------------------------------89       \---- HS 4115553(239)
|            |   |                                               |
|            |   |                                               \------------ PS 140964(279)
|            |   |
|            |   |                                               /------------ CR C 670066(133)
|            |   |                                               |
|            |   +----------------------------------------------86   /-------- PS 109123(254)
|            |   |                                               |   |
|            |   |                                               \--55   /---- PS 121412(256)
|            |   |                                                   \-100
|            |   |                                                       \---- LM F19.0260(459)
|            |   |
|            |   |                                                       /---- HS HCP41593(213)
|            |   \-----------------------------------------------------100
|            |                                                           \---- HS 12654739(222)
|            |
|            |                                                           /---- AT 4G05190(39)
|            |                                                       /-100
|            |                                                       |   \---- AT 4G21270(42)
|            |                                                   /--88
|            |                                                   |   \-------- PT LG XI0089(390)
|            |                                                   |
|            |                                              /---59       /---- AT 4G27180(44)
|            |                                              |    |   /-100
|            |                                              |    |   |   \---- AT 5G54670(57)
|            |                                              |    \-100
|            |                                              |        \-------- PT LG XI000300(433)
|            |                                              |
|            |                                          /--64            /---- OS SBCC023227(322)
|            |                                          |   |        /--92
|            |                                          |   |        |   \---- OS IBCD022126(365)
|            |                                          |   |    /--90
|            |                                          |   |    |   |   /---- OS SBSC051135(334)
|            |                                          |   |    |   \-100
|            |                                      /-100   \--100       \---- OS IBCD012736(351)
```

```
|             |                                     |   |         |
|             |                                     |   |         \------------ OS IBCD031252(375)
|        /---55                                     |   |
|        |    |                                 /--99   |                 /---- OS SBCC016426(313)
|        |    |                                 |   |   \---------------100
|        |    |                                 |   |                     \---- OS IBCD015695(356)
|        |    |                                 |   |
|        |    |                                 |   \-------------------------- CR C 60218(131)
|        |    |                                 |
|        |    |                             /--71                         /---- TP 124560(85)
|        |    |                             |   |                     /--57
|        |    |                             |   |                     |   \---- PS 129079(260)
|        |    |                             |   |                 /-100
|        |    |                             |   |                 |   |   /---- TP 151754(90)
|        |    |                             |   |                 |   \--56
|        |    |                             |   \----------------99       \---- PS 124499(291)
|        |    |                             |                     |
|        |    |                             |                     \------------ CR C 120136(115)
|        |    |                             |
|        |    |                             |               /------------------ DD B0189377(67)
|        |    |                             |               |
|        |    |                         /--65               |             /---- SC KAR3(496)
|        |    |                         |   |               |         /--96
|        |    |                         |   |           /--97         |   \---- SP AC3A11.14C(521)
|        |    |                         |   |           |   |     /-100
|        |    |                         |   |           |   |     |   \-------- SP AC664.10(522)
|        |    |                         |   +----------80   \--100
|        |    |                         |   |           |         \------------ PC GW.73.22.1(503)
|        |    |                         |   |           |
|        |    |                         |   |           \---------------------- HS 3702453(231)
|        |    |                         |   |
|        |    |                         |   +---------------------------------- GL 8886(138)
|        |    |                         |   |
|        |    |                         |   \---------------------------------- CM R497C(493)
|        |    |                         |
|        |    |                         |                                 /---- CE C41G7.2(98)
|        |    |                    /---50                             /-100
|        |    |                    |    |                             |   \---- CE M01E11.6(102)
|        |    |                    |    |                         /-100
|        |    |                    |    |                         |   \-------- CE W02B12.7(108)
|        |    |                    |    |                   /---56
|        |    |                    |    |                   |     \------------ LM F28.1850(472)
|        |    |                    |    +------------------54
|        |    |                    |    |                   |             /---- CI 0100133346(170)
|        |    |                    |    |                   \------------86
|        |    |                    |    |                                 \---- DM CG7831 PA(205)
|        |    |                    |    |
|        |    |                    |    +-------------------------------------- PS 137761(275)
|        |    |                    |    |
|        |    |                    |    |                                 /---- PS 121577(292)
|        |    |                    |    \--------------------------------68
|        |    |                    |                                      \---- TP 152375(528)
|        |    \-------------------57
|        |                         |                                      /---- AT 5G10470(49)
|        |                         |                                  /-100
|        |                         |                                  |   \---- AT 5G65460(59)
|        |                         |                                  |
|        |                         |                                  |   /---- OS SBCC020793(320)
|        |                         |                /---------------100-100
|        |                         |                |                 |   \---- OS IBCD019841(363)
|        |                         |                |                 |
|        |                         |                |                 \-------- PT LG VII001210(422)
|        |                         |                |
|        |                         |                |                     /---- AT 5G65930(60)
|        |                         |                |                 /-100
|        |                         |                |                 |   \---- PT LG XI1218(391)
|        |                         |                |             /-100
|        |                         \---------------95             |   |   /---- OS SBCC016659(314)
|        |                                          |             |   \-100
|        |                                          |       /--100        \---- OS IBCD015937(357)
|        |                                          |       |     |
|        |                                          |       |     \------------ CR C 1030008(114)
|        |                                          |   /-100
|        |                                          |   |   |         /-------- TP 12124(81)
|        |                                          |   |   |         |
|        |                                          |   |   \--------54   /---- CI 0100131201(166)
|        |                                          \-100             \-100
|        |                                              |                 \---- PS 141193(281)
|        |                                              |
|        |                                              \---------------------- GL 13797(141)
|        |
```

```
|       |                                                              /---- AT 1G18370(4)
|       |                                                          /-100
|       |                                                          |   \---- PT LG XII000185(434)
|       |                                                      /--93
|       |                                                      |   |   /---- OS SBCC002024(295)
|       |                                                      |   \-100
|       |                                         /----------100       \---- OS IBCD001985(336)
|       |                                         |            |
|       |                                         |            |       /---- AT 3G43210(30)
|       |                                         |            \-----100
|       |                                         |                    \---- PT SCAFFOLD 2800(428)
|       |                                         |
|       |                                         |                    /---- AT 2G21300(14)
|       |                                         |                /-100
|       |                                         |                |   \---- AT 4G38950(45)
|       |                                         |            /-100
|       |                                         |            |   \-------- PT LG IX0409(388)
|       |                                         |       /--100
|       |                                     /-100       |    |       /---- OS SBCC007775(300)
|       |                                     |   |       |    \-----100
|       |                                     |   |       |            \---- OS IBCD007402(341)
|       |                                     |   |       |
|       |                                     |   |       |            /---- AT 3G51150(36)
|       |                                     |   |   /--87        /-100
|       |                                     |   |   |   |        |   \---- AT 5G66310(61)
|       |                                     |   |   |   |    /-100
|       |                                     |   |   |   |    |   \-------- PT 570109(385)
|       |                                     |   |   |   |    |
|       |                                     |   |   |   \---54       /---- AT 4G24170(43)
|       |                                     |   |   |        |   /-100
|       |                                 /--99   \-100        |   |   \---- AT 5G42490(55)
|       |                                 |   |       |        \--53
|       |                                 |   |       |            \-------- PT LG II000243(418)
|       |                                 |   |       |
|       |                                 |   |       |                /---- OS SBCC028926(324)
|       |                                 |   |       |            /-100
|       |                                 |   |       |            |   \---- OS IBCD027313(369)
|       |                             /--97   |       \----------100
|       |                             |   |   |                    |   /---- OS SBCC030982(329)
|       |                             |   |   |                    \-100
|       |                             |   |   |                        \---- OS IBCD029182(373)
|       |                             |   |   |
|       |                             |   |   \----------------------------- CR C 1480016(119)
|       |                             |   |
|       |                             |   \--------------------------------- CM Q429C(492)
|       |                             |
|       |                             |                                /---- AT 1G21730(8)
|       |                             |                            /--54
|       |                             |                            |   \---- PT 00020745(395)
|       |                             |                   /-------97
|       |                             |                   |        |   /---- OS SBCC015147(312)
|       |                             |                   |        \-100
|       |                             |                   |            \---- OS IBCD014393(354)
|       |                             |                   |
|       |                        /---78               /-100            /---- AT 3G12020(23)
|       |                        |    |               |   |        /-100
|       |                        |    |               |   |        |   \---- AT 5G06670(48)
|       |                        |    |               |   |    /-100
|       |                        |    |               |   |    |   \-------- PT 00160532(407)
|       |                        |    |               |   \--100
|       |                        |    |               |        |       /---- OS SBCC008574(302)
|       |                        |    |           /-100        \-----100
|       |                        |    |           |   |                \---- OS IBCD008139(343)
|       |                        |    |           |   |
|       |                        |    |           |   |                /---- AT 2G21380(15)
|       |                        |    |           |   |            /--98
|       |                        |    |           |   |            |   \---- AT 4G39050(46)
|       |                        |    |       /--79   \----------100
|       |                        |    |       |   |                |   /---- OS IBCD031186(374)
|       |                        |    |       |   |                \--94
|       |                        |    |       |   |                    \---- PT LG IV1283(387)
|       |                        |    \------97   |
|       |                        |            |   \------------------------- DD B0189194(65)
|       |                        |            |
|       |                        |            \----------------------------- PS 138580(276)
|       |                        |
|       |                        |                                     /---- AT 1G59540(10)
|       |                        |                                 /-100
|       |                        |                                 |   \---- PT LG XIV000016(435)
|       |                        |                             /-100
|       +-----------------------54                             |   |   /---- OS SBCC035459(330)
```

```
|       |                         |                              |   \-100
|       |                         |                              |       \---- OS IBCD033171(376)
|       |                         |                         /--100
|       |                         |                         |    |       /---- AT 3G10180(21)
|       |                         |                         |    |   /-100
|       |                         |                         |    |   |   \---- PT LG VI0681(389)
|       |                         |                         |    \-100
|       |                         +------------------------61        |   /---- OS SBCC030333(328)
|       |                         |                         |        \-100
|       |                         |                         |            \---- OS IBCD028585(372)
|       |                         |                         |
|       |                         |                         \----------------- DD B0186344(63)
|       |                         |
|       |                         |                                      /---- TP 121289(82)
|       |                         +-------------------------------------54
|       |                         |                                      \---- PS 127973(257)
|       |                         |
|       |                         |                                      /---- CI 0100133471(171)
|       |                         |                                  /--97
|       |                         |                                  |   \---- HS 399227(237)
|       |                         +---------------------------------71
|       |                         |                                  |   /---- DM CG32955 PE(199)
|       |                         |                                  \-100
|       |                         |                                      \---- DM CG6392 PA(202)
|       |                         |
|       |                         \------------------------------------------- PF L0545W(515)
|       |
|       |                                                                /---- AT 1G18550(6)
|       |                                                            /-100
|       |                                                            |   \---- PT 00120495(402)
|       |                                                        /-100
|       |                                                        |   |   /---- OS SBCC002630(296)
|       |                                                        |   \-100
|       |                                   /-------------------67       \---- OS IBCD002607(337)
|       |                                   |                    |
|       |                                   |                    \------------ PF C0860W(514)
|       |                                   |
|       |                                   |                        /-------- AT 3G49650(34)
|       |                                   |                        |
|       |                                   |                    /-100   /---- OS SBCC012732(309)
|       |                                   |                    |   \-100
|       |                                   |               /--100       \---- OS IBCD012108(350)
|       |                                   |               |    |
|       |                                   |           /--83    \------------ CR C 1310024(117)
|       +---------------------------------100           |   |
|       |                                   |       /--98   \----------------- PS 141959(283)
|       |                                   |       |   |
|       |                                   |       |   \--------------------- PF A0535C(512)
|       |                                   |   /--99
|       |                                   |   |   |                /-------- CE 7499692(112)
|       |                                   |   |   |                |
|       |                                   |   |   \---------------99   /---- DM CG9913 PA(210)
|       |                                   |   |                    \--66
|       |                                   |   |                        \---- HS 23397458(225)
|       |                                   |   |
|       |                                   \--76                        /---- DD B0219839(74)
|       |                                       |       /---------------62
|       |                                       |       |                \---- GL 4371(154)
|       |                                       |       |
|       |                                       |       |            /-------- DM CG10923 PA(188)
|       |                                       |       |            |
|       |                                       \------98   /------100   /---- HS HCP43728(214)
|       |                                               |   |        \-100
|       |                                               |   |            \---- HS 12053149(221)
|       |                                               |   |
|       |                                               |   |            /---- SC KIP3(499)
|       |                                               \--62        /--81
|       |                                                   |        |   \---- SP BC1685.15C(525)
|       |                                                   |    /-100
|       |                                                   |    |   \-------- SP BC2F12.13(526)
|       |                                                   \--100
|       |                                                        \------------ PC PC.95.48.1(508)
|       |
|       |                                                                /---- AT 1G20060(7)
|       |                                                            /--85
|       |                                                            |   \---- PT LG XIII0655(392)
|       |                                                        /-100
|       |                                                        |   |   /---- OS SBCC004978(299)
|       |                                                        |   \-100
|       |                              /------------------------99       \---- OS IBCD004715(339)
|       |                              |                         |
```

```
|       |                             |                        \------------ TP 137385(88)
|       |                             |
|       |                             |       /----------------------------- DD B0189854(68)
|       |                             |       |
|       |                             |       |           /----------------- CE M03D4.1A(103)
|       |                             |       |           |
|       |                             |       |           |        /-------- CI 0100154383(185)
|       |                        /---71       |       /-100        |
|       |                        |    |       |       |   |    /-100   /---- HS HCP49543(218)
|       |                        |    |   /--55       |   |    |   \-100
|       |                        |    |   |   |       |   \--100       \---- HS 400264(238)
|       |                        |    |   |   |   /--50        |
|       |                        |    |   |   |   |   |        \------------ DM CG1258 PA(191)
|       |                        |    |   |   |   |   |
|       |                        |    |   |   |   |   \--------------------- PC PC.11.256.1(504)
|       |                        |    |   |   \--78
|       +-----------------------72    \--71       |                /-------- CI 0100130230(162)
|       |                        |        |       |                |
|       |                        |        |       \--------------100   /---- HS 3978240(236)
|       |                        |        |                        \--98
|       |                        |        |                            \---- HS 5911999(245)
|       |                        |        |
|       |                        |        \--------------------------------- PS 140636(278)
|       |                        |
|       |                        \------------------------------------------ DM CG12298 PA(190)
|       |
|       |                                                              /---- AT 2G28620(17)
|       |                                                          /--64
|       |                                                          |   \---- AT 3G45850(33)
|       |                                                      /-100
|       |                                                      |   |   /---- PT 00160756(408)
|       |                                                      |   \--83
|       |                                                 /--100       \---- PT LG I001798(416)
|       |                                                 |    |
|       |                                                 |    |       /---- OS SBCC016982(315)
|       |                                                 |    \-----100
|       |                                                 |            \---- OS IBCD016250(358)
|       |                                             /--96
|       |                                             |   |    /------------ AT 2G37420(19)
|       |                                             |   |    |
|       |                                             |   |    |       /---- OS SBCC010233(306)
|       |                                             |   \--100   /-100
|       |                                             |        |   |   \---- OS IBCD009684(347)
|       |                                         /-100        \--55
|       |                                         |   |            \-------- PT 00060694(398)
|       |                                         |   |
|       |                                         |   |                /---- AT 2G36200(18)
|       |                                         |   |            /-100
|       |                                         |   |            |   \---- PT LG XVI0226(393)
|       |                                     /-100   \----------100
|       |                                     |   |                |   /---- OS SBCC028993(325)
|       |                                     |   |                \-100
|       |                                     |   |                    \---- OS IBCD027399(370)
|       |                                     |   |
|       |                                     |   \------------------------- CR C 530027(129)
|       |                                     |
|       |                                     |                        /---- CE F23B12.8(99)
|       |                                     |                    /--56
|       |                                 /-100                    |   \---- DM CG9191 PA(209)
|       |                                 |   |                /-100
|       |                                 |   |                |   \-------- HS 1706622(224)
|       |                                 |   |           /---85
|       |                                 |   |           |    \------------ PS 108445(252)
|       |                                 |   |       /--87
|       |                                 |   |       |   \----------------- CM C157C(490)
|       |                                 |   |       |
|       |                                 |   |       |                /---- SC CIN8(495)
|       |                             /--71   \------71            /--52
|       |                             |   |           |            |   \---- SC KIP1(497)
|       |                             |   |           |        /-100
|       |                             |   |           |        |   \-------- SP AC25G10.07C(520)
|       |                             |   |           \------100
|       |                             |   |                    \------------ PC PC.41.67.1(507)
|       |                        /---98   |
|       |                        |    |   |                            /---- GL 16425(148)
|       |                        |    |   \--------------------------100
|       |                        |    |                                \---- PF C0770C(513)
|       +-----------------------55    |
|       |                        |    \------------------------------------- DD B0187903(64)
|       |                        |
|       |                        \------------------------------------------ TP 135880(87)
```

```
    |       |
    |       |                                                              /---- AT 3G16060(24)
    |       |                                                 /----------100
    |       |                                                 |            \---- OS SBCC002748(297)
    |       |                                                 |
    |       |                                                 |       /-------- AT 3G16630(25)
    |       |                                             /-100       |
    |       |                                             |   |   /-100   /---- PT 00100152(399)
    |       |                                             |   |   |   \--86
    |       |                                             |   |   |       \---- PT 02100029(411)
    |       |                                             |   \--100
    |       |                                         /-100       |       /---- OS SBCC017229(316)
    |       |                                         |   |       \-----100
    |       |                                         |   |                \---- OS IBCD016493(359)
    |       |                                 /------75   |
    |       |                                 |       |   \--------------------- CR C 730040(135)
    |       |                                 |       |
    |       |                                 |       \------------------------- PF L2165W(516)
    |       |                                 |
    |       |                                 |   /----------------------------- DD B0216601(71)
    |       |                             /--81   |
    |       |                             |   |   |                        /---- TP 112325(78)
    |       |                             |   |   |                    /--99
    |       |                             |   |   |                    |   \---- PS 136489(269)
    |       |                             |   |   |                /--81
    |       |                             |   \--75                |   \-------- PS 135741(267)
    |       |                             |       |   /-----------81
    |       |                             |       |   |            \------------ PS 136872(270)
    |       |                             |       |   |
    |       |                             |       |   |   /--------------------- CE K11D9.1A(100)
    |       |                             |       \--81   |
    |       |                             |           |   |        /------------ CI 0100146176(182)
    |       |                             |           |   |        |
    |       |                        /---81           |   |   /---90   /-------- HS 1695882(223)
    |       |                        |    |           \-100   |    |   |
    |       |                        |    |               |   |    \--72   /---- HS 3024057(229)
    |       |                        |    |               |   |        \-100
    |       |                        |    |               |   |            \---- HS 49355831(243)
    |       |                        |    |               \--99
    |       |                        |    |                   |            /---- DM CG12192 PA(189)
    |       |                        |    |                   |        /-100
    |       |                        |    |                   |        |   \---- DM CG3219 PA(198)
    |       |                        |    |                   \------100
    |       |                    /--81    |                            \-------- DM CG1453 PA(192)
    |       |                    |   |    |
    |       |                    |   |    |                                /---- TP 116492(79)
    |       |                    |   |    \------------------------------66
    |       |                    |   |                                     \---- GL 16945(151)
    |       |                    |   |
    |       |                /--56   |                                     /---- LM F01.0030(436)
----+       |                |   |   |                                 /--94
    |       |                |   |   |                                 |   \---- LM F13.1610(446)
    |       |                |   |   \--------------------------------55
    |       |            /--78   |                                     \-------- LM F24.0640(468)
    |       |            |   |   |
    |       |            |   |   \---------------------------------------------- LM F31.0290(479)
    |       |            |   |
    |       |            |   \-------------------------------------------------- LM F13.0130(444)
    |       +----------100
    |       |            |                                             /-------- TP 107522(76)
    |       |            |                                             |
    |       |            |                                         /--88   /---- LM F35.4700(488)
    |       |            |                                         |   \--59
    |       |            \----------------------------------------91       \---- PC PC.13.63.1(505)
    |       |                                                      |
    |       |                                                      \------------ PS 142524(285)
    |       |
    |       |                                                      /------------ AT 3G17360(26)
    |       |                                                      |
    |       |                                                      |       /---- AT 3G19050(27)
    |       |                                                 /--100   /-100
    |       |                                                 |    |   |   \---- PT 01300020(409)
    |       |                                                 |    \-100
    |       |                                                 |        |   /---- OS SBCC038188(332)
    |       |                                             /-100        \-100
    |       |                                             |   |            \---- OS IBCD035525(378)
    |       |                                             |   |
    |       |                                             |   |            /---- AT 3G44050(31)
    |       |                                         /-100   \----------100
    |       |                                         |   |                \---- PT LG IX000131(421)
    |       |                                         |   |
```

```
|       |                                         |   \--------------------- CR C 1350007(118)
|       |                                         |
|       |                                         |                    /---- AT 3G20150(28)
|       |                                         |                /--98
|       |                                     /-100                |   \---- PT 00012593(394)
|       |                                     |   |       /------100
|       |                                     |   |       |        |   /---- OS SBCC011576(308)
|       |                                     |   |       |        \-100
|       |                                     |   |       |            \---- OS IBCD010981(349)
|       |                                     |   |       |
|       |                                     |   |       |            /---- AT 3G23670(29)
|       |                                     |   \-----100        /-100
|       |                                     |           |        |   \---- AT 4G14150(40)
|       |                                 /-100           |    /-100
|       |                                 |   |           |    |   |   /---- PT LG XIV000891(427)
|       |                                 |   |           |    |   \-100
|       |                                 |   |           \--100       \---- PT LG II001048(431)
|       |                                 |   |                |
|       |                                 |   |                |       /---- OS SBCC014640(310)
|       |                                 |   |                \-----100
|       |                                 |   |                        \---- OS IBCD013910(352)
|       |                                 |   |
|       +--------------------------------86   \----------------------------- CM O070C(491)
|       |                                 |
|       |                                 |                            /---- TP 132625(86)
|       |                                 |                        /--84
|       |                                 |                        |   \---- PS 143005(290)
|       |                                 |               /-------86
|       |                                 |               |        \-------- PS 141849(282)
|       |                                 |               |
|       |                                 |               |            /---- CE C06G3.2(96)
|       |                                 \--------------86    /-----100
|       |                                                 |    |       \---- CE C33H5.4A(97)
|       |                                                 |    |
|       |                                                 \--100   /-------- CI 0100130295(163)
|       |                                                      |   |
|       |                                                      \--98   /---- HS 9910266(250)
|       |                                                          \-100
|       |                                                              \---- MM 40644653(381)
|       |
|   /-100
|   |   |                                                              /---- AT 3G50240(35)
|   |   |                                                      /------64
|   |   |                                                      |       \---- PT LG VII000272(432)
|   |   |                                                      |
|   |   |                                                 /--100       /---- AT 5G47820(56)
|   |   |                                                 |    |   /--55
|   |   |                                                 |    |   |   \---- PT LG II000522(429)
|   |   |                                                 |    \--97
|   |   |                                                 |        |   /---- OS SBCC029113(326)
|   |   |                                                 |        \-100
|   |   |                                                 |            \---- OS IBCD027529(371)
|   |   |                                             /-100
|   |   |                                             |   |        /-------- AT 5G60930(58)
|   |   |                                             |   |        |
|   |   |                                             |   |    /-100   /---- PT 00020976(396)
|   |   |                                             |   |    |   \-100
|   |   |                                             |   |    |       \---- PT 02310007(412)
|   |   |                                     /-----100   \--100
|   |   |                                     |       |        |       /---- OS SBCC008366(301)
|   |   |                                     |       |        \-----100
|   |   |                                     |       |                \---- OS IBCD007936(342)
|   |   |                                     |       |
|   |   |                                     |       \--------------------- CR C 120157(116)
|   |   |                                     |
|   |   |                                     |                        /---- DD B0218612(73)
|   |   |                                     |   /------------------100
|   |   |                                     |   |                    \---- PS 142668(287)
|   |   |                                     |   |
|   |   |                                     |   |       /----------------- CE T01G1.1A(106)
|   |   |                                     |   |       |
|   |   |                                 /--97--50       |        /-------- CI 0100130413(164)
|   |   |                                 |   |   |   /-100        |
|   |   |                                 |   |   |   |   |    /--75   /---- HS HCP1631302(211)
|   |   |                                 |   |   |   |   |    |   \-100
|   |   |                                 |   |   |   |   \---51       \---- HS 5360129(244)
|   |   |                                 |   |   \--95        |
|   |   |                                 |   |       |        \------------ DM CG5300 PA(200)
|   |   |                                 |   |       |
|   |   |                                 |   |       |                /---- CR C 260133(124)
|   |   |                                 |   |       \---------------98
|   |   |                                 |   |                        \---- CR C 340020(125)
```

```
|   |   |                                 |   |
|   |   +--------------------------------75   |                        /---- CI 0100131275(168)
|   |   |                                 |   \----------------------100
|   |   |                                 |                            \---- HS 7266951(248)
|   |   |                                 |
|   |   |                                 |                            /---- TP 119229(80)
|   |   |                                 |                            |
|   |   |                                 +---------------------------97---- GL 16650(150)
|   |   |                                 |                            |
|   |   |                                 |                            \---- DM CG8590 PA(208)
|   |   |                                 |
|   |   |                                 |                            /---- HS HCP44045(215)
|   |   |                                 \---------------------------77
|   |   |                                                              \---- PC PC.3.10.1(506)
|   |   |
|   |   |                                                          /-------- AT 3G63480(38)
|   |   |                                                          |
|   |   |                                                      /-100   /---- OS SBCC026309(323)
|   |   |                                                      |   \-100
|   |   |                                     /---------------65       \---- OS IBCD024826(366)
|   |   |                                     |                |
|   |   |                                     |                \------------ PS 142980(289)
|   |   |                                     |
|   |   |                                     +----------------------------- DD B0204045(69)
|   |   |                                     |
|   |   |                                     |   /------------------------- CE R05D3.7(104)
|   |   |                                     |   |
|   |   |                                     |   |            /------------ CI 0100143504(178)
|   |   |                                     |   |            |
|   |   |                                 /--53   |            |       /---- HS 2497520(226)
|   |   |                                 |   +-100       /--100   /-100
|   |   |                                 |   |   |       |    |   |   \---- HS 304358(230)
|   |   |                                 |   |   |       |    \--97
|   |   |                                 |   |   |   /--91        \-------- HS 417216(240)
|   |   |                                 |   |   |   |   |
|   |   |                                 |   |   \--56   \----------------- SPU 47550911(527)
|   |   |                                 |   |       |
|   |   |                                 |   |       \--------------------- DM CG7765 PA(204)
|   |   |                                 |   |
|   |   |                                 |   \----------------------------- PS 130051(261)
|   |   |                                 |
|   |   +--------------------------------86                    /------------ DD B0166988(62)
|   |   |                                 |                    |
|   |   |                                 |                    |       /---- TP 133437(94)
|   |   |                                 +-------------------68   /--97
|   |   |                                 |                    |   |   \---- TP 128511(529)
|   |   |                                 |                    \-100
|   |   |                                 |                        \-------- PS 137039(271)
|   |   |                                 |
|   |   |                                 +--------------------------------- GL 13825(142)
|   |   |                                 |
|   |   |                                 |                            /---- PC GW.41.36.1(502)
|   |   |                                 \--------------------------100
|   |   |                                                              \---- SP AC1834.07(519)
|   |   |
|   |   |                                                          /-------- AT 4G14330(41)
|   |   |                                                          |
|   |   |                                                          |   /---- OS SBCC008809(303)
|   |   |                                                          +-100
|   |   +--------------------------------------------------------100   \---- OS IBCD008347(344)
|   |   |                                                          |
|   |   |                                                          |   /---- PT LG VIII001511(424)
|   |   |                                                          \-100
|   |   |                                                              \---- PT LG X000373(425)
|   |   |
|   |   |                                                          /-------- AT 5G02370(47)
|   |   |                                                          |
|   |   |                                                      /-100   /---- OS SBCC019161(318)
|   |   |                                                      |   \-100
|   |   |                                                      |       \---- OS IBCD018270(361)
|   |   |                                                 /---87
|   |   |                                                 |    |   /-------- AT 5G23910(50)
|   |   |                                                 |    |   |
|   |   |                                                 |    \--87   /---- OS SBCC014774(311)
|   |   |                                                 |        \-100
|   |   +------------------------------------------------87            \---- OS IBCD014035(353)
|   |   |                                                 |
|   |   |                                                 |        /-------- CI 0100141905(177)
|   |   |                                                 |        |
|   |   |                                                 \------100   /---- HS 4519443(241)
|   |   |                                                          \-100
```

```
|   |   |                                                              \---- MM 21704182(380)
|   |   |
|   |   |                                             /--------------------- DD B0189215(66)
|   |   |                                             |
|   |   |                                             |                /---- CE R144.1(105)
|   |   |                                             |            /--77
|   |   |                                             |            |   \---- CI 0100136694(175)
|   |   |                                         /--99        /--98
|   |   |                                         |   |        |   \-------- CI 0100137520(176)
|   |   |                                         |   |   /--100
|   |   |                                         |   |   |    \------------ CI 0100134202(173)
|   |   |                                         |   \-100
|   |   |                                     /--97       \----------------- PS 108377(251)
|   |   |                                     |   |
|   |   |                                     |   |                    /---- GL 102101(155)
|   |   |                                     |   |                /-100
|   |   |                                     |   |                |   \---- GL 112846(160)
|   |   |                                 /--79   \--------------100
|   |   |                                 |   |                    \-------- GL 6262(157)
|   |   |                                 |   |
|   |   |                                 |   |                        /---- DM CG5658 PA(201)
|   |   |                                 |   \----------------------100
|   |   |                                 |                            \---- HS 6522736(247)
|   |   |                                 |
|   |   |                             /--72               /----------------- CE UNC 104(107)
|   |   |                             |   |               |
|   |   |                             |   |           /-100    /------------ CI 0100131206(167)
|   |   |                             |   |           |   |    |
|   |   |                             |   |           |   \--100   /-------- DM CG8566 PD(207)
|   |   |                             |   |           |        |   |
|   |   |                             |   \----------89        \--99   /---- HS 2497523(227)
|   |   |                             |               |            \-100
|   |   |                             |               |                \---- HS 3913961(235)
|   |   |                        /---66               |
|   |   |                        |    |               \--------------------- PC GW.4.121.1(501)
|   |   |                        |    |
|   |   |                        |    |               /--------------------- CE F56E3.3(110)
|   |   |                        |    |               |
|   |   |                        |    |               |                /---- CI 0100143686(179)
|   |   |                        |    |               |        /------75
|   |   |                        |    |               |        |       \---- PS 128382(259)
|   |   |                        |    \--------------75        |
|   |   |                        |                    |   /---75       /---- HS HCP34632(212)
|   |   |                        |                    |   |    |   /-100
|   |   |                        |                    |   |    |   |   \---- HS 8896164(249)
|   |   |                        |                    \--74    \-100
|   |   |                        |                        |        \-------- HS 11761611(220)
|   |   |                        |                        |
|   |   |                        |                        \----------------- DM CG8183 PA(206)
|   |   |                        |
|   |   |                        |                                     /---- CI 0100130156(161)
|   |   |                        |                                 /-100
|   |   |                        |                                 |   \---- HS 452517(242)
|   |   |                        +-------------------------------100
|   |   |                        |                                 \-------- DM CG10718 PA(187)
|   |   |                        |
|   |   |                        |                                     /---- LM F14.0810(447)
|   |   |                        +------------------------------------95
|   |   |                    /--56                                     \---- LM F23.0560(467)
|   |   |                    |   |
|   |   |                    |   |                                     /---- LM F14.1100(448)
|   |   |                    |   |                                     |
|   |   |                    |   |                             /-----100---- LM F14.1110(449)
|   |   |                    |   |                             |       |
|   |   |                    |   |                             |       \---- LM F14.1120(450)
|   |   |                    |   |                             |
|   |   |                    |   |                        /---97       /---- LM F16.1460(451)
|   |   |                    |   |                        |    |   /-100
|   |   |                    |   |                        |    |   |   \---- LM F16.1470(452)
|   |   |                    |   |                    /--68    \-100
|   |   |                    |   |                    |   |        \-------- LM F29.0970(473)
|   |   |                /--99   |                    |   |
|   |   |                |   |   |                    |   \----------------- LM F22.0960(466)
|   |   |                |   |   |                    |
|   |   |                |   |   +-------------------84                /---- LM F19.0680(460)
|   |   |                |   |   |                    |            /-100
|   |   |                |   |   |                    |            |   \---- LM F19.0690(461)
|   |   |                |   |   |                    |        /-100
|   |   |                |   |   |                    |        |   \-------- LM F19.0700(462)
|   |   |                |   |   |                    \-------68
|   |   |            /-100   |   |                             \------------ LM F21.1040(464)
```

```
|   |   |            |   |   |   |
|   |   |            |   |   |   \------------------------------------------ SP AC144.14(518)
|   |   |            |   |   |
|   |   |            |   |   \---------------------------------------------- LM F34.4260(486)
|   |   |            |   |
|   |   |            |   |                                             /---- LM F29.2380(474)
|   |   |        /-100   |                                         /-100
|   |   |        |   |   |                                         |   \---- LM F29.2390(475)
|   |   |        |   |   \----------------------------------------97
|   |   |        |   |                                             \-------- LM F33.2560(484)
|   |   |        |   |
|   |   |        |   |                                                 /---- PS 141078(280)
|   |   |        |   \-----------------------------------------------100
|   |   |        |                                                     \---- PS 142793(288)
|   |   |        |
|   |   |        |                                                     /---- CE M02B7.3A(101)
|   |   |        |                                                     |
|   |   |        |                                                 /-100---- CI 0100134965(174)
|   |   |        |                                                 |   |
+--98   |        |                        /-----------------------95   \---- HS HCP48743(217)
|   |   |        |                        |                        |
|   |   |        |                        |                        \-------- DM CG17461 PA(196)
|   |   |        |                        |
|   |   |        |                        |                    /------------ CE F20C5.2B(111)
|   |   |        |                        |                    |
|   |   |        |                        |               /---76   /-------- CI 0100133723(172)
|   |   +-------51                        |               |    |   |
|   |   |        |                        |               |    \--89   /---- HS 3913957(233)
|   |   |        |                        |           /--56        \--97
|   |   |        |                        |           |   |            \---- HS 3913958(234)
|   |   |        |                    /--98           |   |
|   |   |        |                    |   |           |   \----------------- DM CG7293 PA(203)
|   |   |        |                    |   |       /-100
|   |   |        |                    |   |       |   |                /---- CI 0100148992(183)
|   |   |        |                    |   |       |   |            /-100
|   |   |        |                    |   |       |   |            |   \---- HS 3851492(232)
|   |   |        |                    |   |   /--53   \----------100
|   |   |        |                    |   |   |   |                \-------- DM CG10642 PA(186)
|   |   |        |                    |   |   |   |
|   |   |        |               /---97   |   |   |                    /---- CR C 160226(120)
|   |   |        |               |    |   \-100   \-------------------75
|   |   |        |               |    |       |                        \---- CR C 1880008(121)
|   |   |        |               |    |       |
|   |   |        |               |    |       |                        /---- PS 108563(253)
|   |   |        |               |    |       \----------------------100
|   |   |        |               |    |                                \---- PS 120306(255)
|   |   |        \--------------98    |
|   |   |                        |    |                                /---- GL 16456(149)
|   |   |                        |    \-------------------------------98
|   |   |                        |                                     \---- GL 17333(153)
|   |   |                        |
|   |   |                        |                                     /---- LM F17.0800(455)
|   |   |                        \-----------------------------------100
|   |   |                                                              \---- LM F32.0680(482)
|   |   |
|   |   +------------------------------------------------------------------- DD B0204609(70)
|   |   |
|   |   +------------------------------------------------------------------- DD B0217523(72)
|   |   |
|   |   +------------------------------------------------------------------- TP 105812(75)
|   |   |
|   |   |                                                              /---- TP 110182(77)
|   |   |                                                          /-100
|   |   |                                                          |   \---- TP 156428(92)
|   |   +--------------------------------------------------------100
|   |   |                                                          \-------- PS 134623(266)
|   |   |
|   |   |                                                              /---- TP 142737(89)
|   |   |                                                          /--99
|   |   |                                                          |   \---- PS 137042(272)
|   |   |                                                      /-100
|   |   |                                                      |   \-------- CR C 250150(122)
|   |   |                                                      |
|   |   |                                                      |       /---- GL 10137(139)
|   |   |                                                 /--100------52
|   |   |                                                 |    |       \---- LM F05.0630(437)
|   |   |                                                 |    |
|   |   |                                             /--94    \------------ CI 0100145354(181)
|   |   |                                             |   |
|   |   |                                             |   \----------------- PS 136418(268)
|   |   |                                             |
```

```
|   |   |                                             |           /-------- CR C 50080(127)
|   |   |                                             |           |
|   |   |                                             |       /--99   /---- PS 130522(263)
|   |   +------------------------------------------100        |   \-100
|   |   |                                             |       |       \---- PS 139555(277)
|   |   |                                             |   /--100
|   |   |                                             |   |   |   /-------- CI 0100145013(180)
|   |   |                                             |   |   |   |
|   |   |                                             |   |   \-100   /---- HS 11275982(219)
|   |   |                                             \-100       \-100
|   |   |                                                 |           \---- MM 5295882(383)
|   |   |                                                 |
|   |   |                                                 \---------------- GL 6404(158)
|   |   |
|   |   +------------------------------------------------------------------ TP 155696(91)
|   |   |
|   |   |                                                             /---- TP 163717(93)
|   |   +------------------------------------------------------------54
|   |   |                                                             \---- LM F22.0560(465)
|   |   |
|   |   +------------------------------------------------------------------ CE Y43F4B.6(109)
|   |   |
|   |   |                                                 /---------------- CE K12F2.2A(113)
|   |   |                                                 |
|   |   +-----------------------------------------------100    /------------ DM CG14535 PA(193)
|   |   |                                                 |   |
|   |   |                                                 \--100  /-------- HS 29732867(228)
|   |   |                                                     |   |
|   |   |                                                     \--91   /---- HS 6330751(246)
|   |   |                                                         \-100
|   |   |                                                             \---- MM 51767056(382)
|   |   |
|   |   |                                                             /---- CR C 260066(123)
|   |   |                                                         /-100
|   |   |                                                         |   \---- LM F06.0180(439)
|   |   |                                                     /--98
|   |   |                                                     |   \-------- PS 121181(293)
|   |   |                                                     |
|   |   |                                                 /--100      /---- CI 0100131135(165)
|   |   |                                                 |   |   /--81
|   |   |                                                 |   |   |   \---- HS HCP45833(216)
|   |   |                                                 |   \-100
|   |   +-----------------------------------------------96        \-------- DM CG15844 PA(194)
|   |   |                                                 |
|   |   |                                                 |           /---- CR C 620070(132)
|   |   |                                                 \----------96
|   |   |                                                             \---- LM F34.1540(485)
|   |   |
|   |   +------------------------------------------------------------------ CR C 460075(126)
|   |   |
|   |   +------------------------------------------------------------------ CR C 570090(130)
|   |   |
|   |   |                                                             /---- CR C 710026(134)
|   |   |                                                         /--79
|   |   |                                                         |   \---- PS 128334(258)
|   |   |                                                     /-100
|   |   |                                                     |   \-------- LM F25.1970(471)
|   |   +----------------------------------------------------86
|   |   |                                                     |       /---- GL 7874(137)
|   |   |                                                     \------89
|   |   |                                                             \---- GL 16161(146)
|   |   |
|   |   |                                                             /---- CR C 790050(136)
|   |   +------------------------------------------------------------86
|   |   |                                                             \---- LM F20.0640(463)
|   |   |
|   |   |                                                             /---- GL 11442(140)
|   |   +------------------------------------------------------------92
|   |   |                                                             \---- LM F09.0290(442)
|   |   |
|   |   |                                                             /---- GL 14070(143)
|   |   +------------------------------------------------------------67
|   |   |                                                             \---- GL 17264(152)
|   |   |
|   |   |                                                             /---- GL 15134(144)
|   |   +------------------------------------------------------------82
|   |   |                                                             \---- LM F31.2710(480)
|   |   |
|   |   +------------------------------------------------------------------ GL 15962(145)
|   |   |
|   |   +------------------------------------------------------------------ GL 16224(147)
```

```
|   |   |
|   |   +-------------------------------------------------------------------- GL 102455(156)
|   |   |
|   |   +-------------------------------------------------------------------- GL 112729(159)
|   |   |
|   |   +-------------------------------------------------------------------- CI 0100131488(169)
|   |   |
|   |   |                                                               /---- DM CG1708 PA(195)
|   |   +--------------------------------------------------------------52
|   |   |                                                               \---- DM CG1763 PA(197)
|   |   |
|   |   +-------------------------------------------------------------------- PS 130250(262)
|   |   |
|   |   +-------------------------------------------------------------------- PS 131621(264)
|   |   |
|   |   +-------------------------------------------------------------------- PS 132151(265)
|   |   |
|   |   |                                                               /---- PS 137485(273)
|   |   |                                                           /--90
|   |   |                                                           |   \---- PS 142481(284)
|   |   +----------------------------------------------------------77
|   |   |                                                           \-------- PF 07 0104(510)
|   |   |
|   |   |                                                               /---- PS 137622(274)
|   |   +--------------------------------------------------------------58
|   |   |                                                               \---- PF MAL8P1.132(509)
|   |   |
|   |   +-------------------------------------------------------------------- LM F05.0760(438)
|   |   |
|   |   +-------------------------------------------------------------------- LM F06.1030(440)
|   |   |
|   |   +-------------------------------------------------------------------- LM F09.0120(441)
|   |   |
|   |   +-------------------------------------------------------------------- LM F11.0870(443)
|   |   |
|   |   +-------------------------------------------------------------------- LM F13.0700(445)
|   |   |
|   |   +-------------------------------------------------------------------- LM F16.1580(453)
|   |   |
|   |   +-------------------------------------------------------------------- LM F17.0160(454)
|   |   |
|   |   +-------------------------------------------------------------------- LM F17.1110(456)
|   |   |
|   |   +-------------------------------------------------------------------- LM F18.1530(457)
|   |   |
|   |   +-------------------------------------------------------------------- LM F18.1600(458)
|   |   |
|   |   |                                                               /---- LM F24.1430(469)
|   |   +--------------------------------------------------------------90
|   |   |                                                               \---- LM F30.1450(477)
|   |   |
|   |   +-------------------------------------------------------------------- LM F25.1950(470)
|   |   |
|   |   +-------------------------------------------------------------------- LM F30.0350(476)
|   |   |
|   |   +-------------------------------------------------------------------- LM F30.3060(478)
|   |   |
|   |   +-------------------------------------------------------------------- LM F32.0420(481)
|   |   |
|   |   +-------------------------------------------------------------------- LM F33.2140(483)
|   |   |
|   |   +-------------------------------------------------------------------- LM F35.2090(487)
|   |   |
|   |   +-------------------------------------------------------------------- LM F36.5150(489)
|   |   |
|   |   |                                                               /---- SC KIP2(498)
|   |   +-------------------------------------------------------------100
|   |   |                                                               \---- SP BC1604.20C(524)
|   |   |
|   |   +-------------------------------------------------------------------- SC SMY1(500)
|   |   |
|   |   +-------------------------------------------------------------------- PF 11 0478(511)
|   |   |
|   |   +-------------------------------------------------------------------- PF L2190C(517)
|   |   |
|   |   \-------------------------------------------------------------------- SP BC15D4.01C(523)
|   |
|   |                                                                   /---- AT 3G54870(37)
|   |                                                               /--84
|   |                                                               |   \---- PT LG VIII000260(423)
|   \--------------------------------------------------------------99
```

```
|                                                                  |   /---- OS SBCC009381(305)
|                                                                  \-100
|                                                                      \---- OS IBCD008900(346)
|
|                                                                      /---- AT 1G12430(3)
|                                                                  /--99
|                                                                  |   \---- PT LG III0949(386)
|                                                              /--83
|                                                              |   |   /---- OS SBCC020273(319)
|                                                              |   \-100
\-------------------------------------------------------------96       \---- OS IBCD019322(362)
                                                               |
                                                               \------------ PT LG II000635(430)
```
