# Supplementary material for: Comprehensive comparative analysis of kinesins in photosynthetic eukaryotes
Source: BMC Genomics. 2006 Jan 31;7:18. doi: 10.1186/1471-2164-7-18 (PMC1434745; doi:10.1186/1471-2164-7-18)
Supplement: Additional file 2 — Supplemental Table 2. H. sapiens kinesins and their structural features. [file 1471-2164-7-18-S2.pdf]

Supplemental Table 2 - *H. sapiens* kinesins and their structural features

Gene ID	Protein length	EST	Additional Domains	MD location	# of exons	Family
2497520	1032	Yes	CC	N	23	1
3043586[a]	957	Yes	CC	N	27[d]	1
417216	963	Yes	CC	N	25	1
hCP48743	1040	-	CC	N	15	2
3913957	793	Yes	CC	N	8	2
3913958	747	Yes	CC	N	8	2
3851492	702	Yes	CC	N	17	2
6522736[a]	412	Yes		ND	-	3
2497523	1690	Yes	CC, FHA, PH	N	46	3
3913961	1103	Yes	CC, FHA	N	21	3
hCP34632	1520	-	CC	N	36	3
8896164	1826	Yes	CC, FHA, CAP-Gly	N	40	3
11761611	1805	No	CC, FHA	N	39	3
452517[a]	1648	Yes	CC, FHA	I	29	3
hCP1631302	1720	-	CC, WD-40 repeat	N	38	4
5360129[c]	633	Yes	CC	N	-	4
7266951	1232	Yes	CC	N	30	4
hCP44045	348	-		ND	5	4
1706622	1057	Yes		N	22	5
hCP49543	969	-	CC	N	21	6
400264[a]	960	Yes	CC	N	23	6
3978240	890	Yes	CC	N	18	6
5911999	1780	Yes	CC	N	33	6
29732867[b]	512	No		C	-	6
6330751[a]	1481	Yes	CC	N	15[d]	6
399227	2663	Yes	CC	N	50	7
23397458	548	Yes	CC	N	12	8
12053149	898	Yes	CC	N	16	8
hCP43728	433	-		N	8	8
11275982	725	Yes		N	19	9
hCP45833	350	-		ND	10	9
4519443	665	Yes	CC, HHH	N	14	10
9910266	1388	Yes	CC	N	35	12
1695882	725	Yes	CC	I	21	13
3024057	679	Yes	CC	I	19	13
49355831	673	Yes		I	1	13
hCP41593	754	-	CC	C	17	14
12654739	694	Yes	CC	C	15	14
4115553	384	No		ND	8	14
3702453	673	Yes		C	11	14

hCP refers to Celera predictions. All others are NCBI gi accessions. [a]Record replaced by 40788283, 10280530, 40788943, 18274011, 20521808 respectively at NCBI. [b]Record has been discontinued at NCBI. [c]Information on length and number of exons is not available. [d]Refers to exon number for 40788283 and 20521808. Possible kinesins with truncated motor domains (motor cores less than 290 amino acids): 19923949. In our analysis we did not include two human sequences (3043706 and 7243191) as they did not contain a motor domain. However, these are replaced with new sequences (29421178 and 27529873) that do contain a motor domain. These two replaced sequences are not included in our gene tree analysis. ND, Not determined; CC, coiled-coil; FHA, Fork head associated; PH, Pleckstrin Homology; CAP-Gly, Glycine rich domain found in Cytoskeleton Associated Proteins (CAPs); WD-40, A 40 amino acid repeat motif with W and D dipeptides at the terminus; HHH, Helix-hairpin-helix; N, N-terminal; I, Internal; C, C-terminal.
