# Supplementary material for: Comprehensive comparative analysis of kinesins in photosynthetic eukaryotes
Source: BMC Genomics. 2006 Jan 31;7:18. doi: 10.1186/1471-2164-7-18 (PMC1434745; doi:10.1186/1471-2164-7-18)
Supplement: Additional file 3 — Supplemental Table 3. C. elegans kinesins and their structural features. [file 1471-2164-7-18-S3.pdf]

Supplemental Table 3 - *C. elegans* kinesins and their structural features

Gene ID	Protein length	cDNA	Additional Domains	MD location	# of exons	Family
CeR05D3.7	815	Yes	CC	N	7	1
CeM02B7.3[a]	671	Yes	CC	N	9	2
F20C5.2b	1130	Yes[b]	CC	N	20	2
CeR144.1	928	Yes	FHA	N	13	3
CeUNC-104	1584	Yes	CC, FHA, PH	N	22	3
CeF56E3.3	1595	Yes[b]	CC, FHA	N	23	3
CeT01G1.1[a]	1605	Yes	CC, WD-40 repeat	N	20	4
CeF23B12.8	958	Yes	CC	N	8	5
CeM03D4.1[a]	775	Yes	CC	N	8	6
K12F2.2a	1066	Yes	CC	N	15	6
Ce7499692[a]	595	Yes		N	10	8
CeC06G3.2	932	Yes	CC	N	7	12
CeC33H5.4[a]	690	Yes	CC	N	7	12
CeK11d9.1[a]	747	Yes		C/I	7	13
Ce01083	598	Yes[b]	CC	C	11	14
CeC41G7.2	587	Yes		C	4	14
CeM01E11.6	587	Yes		C	4	14
CeW02B12.7	471	Yes		C	5	14
CeY43F4B.6	1083	Yes[b]	CC	N	7	UG

When searching at http://wormbase.org, remove the "Ce" prefix. [a]Obtained from NCBI using gil7499692. b. Partial cDNA confirmation. CC, Coiled-coil; FHA, Fork head associated; PH, Pleckstrin homology; UG, Ungrouped; N, N-terminal; I, Internal; C, C-terminal.
