# Supplementary material for: Comprehensive comparative analysis of kinesins in photosynthetic eukaryotes
Source: BMC Genomics. 2006 Jan 31;7:18. doi: 10.1186/1471-2164-7-18 (PMC1434745; doi:10.1186/1471-2164-7-18)
Supplement: Additional file 4 — Supplemental Table 4. S. cerevisiae kinesins their structural features. [file 1471-2164-7-18-S4.pdf]

Supplemental Table 4 - *S. cerevisiae* kinesins their structural features

Gene ID	Protein length	Microarray expression	Additional Domains	MD location	# of exons	Family
CIN8	1000	Yes	CC	N	1	5
KIP1	1111	Yes	CC	N	1	5
KIP3	805	Yes	CC	N	1	8
SMY1	656	Yes	CC	N	1	11
KIP2	706	Yes	CC	I	1	13
KAR3	729	Yes	CC	C	1	14

CC, Coiled-coil; N, N-terminal; I, Internal; C, C-terminal.
