# Supplementary material for: Comprehensive comparative analysis of kinesins in photosynthetic eukaryotes
Source: BMC Genomics. 2006 Jan 31;7:18. doi: 10.1186/1471-2164-7-18 (PMC1434745; doi:10.1186/1471-2164-7-18)
Supplement: Additional file 5 — Supplemental Table 5. S. pombe kinesins and their structural features. [file 1471-2164-7-18-S5.pdf]

**Supplemental Table 5 - *S. pombe* kinesins and their structural features**

| Gene ID | Protein length | Microarray | Additional Domains | MD location | # of exons | Family |
|---|---|---|---|---|---|---|
| SPAC1834.07 | 554 | Yes | | N | 5 | 1 |
| SPAC144.14 | 511 | Yes | CC | N | 2 | 3 |
| SPAC25G10.07c | 1085 | Yes | CC | N | 1 | 5 |
| SPBC1685.15c | 784 | Yes | CC | N | 2 | 8 |
| SPBC2F12.13 | 883 | Yes | CC | N | 2 | 8 |
| SPBC1604.20c | 628 | Yes | CC | I | 1 | 13 |
| SPAC3A11.14c | 832 | Yes | CC | C | 4 | 14 |
| SPAC664.10 | 817 | Yes | CC | C | 2 | 14 |
| SPBC15D4.01c | 633 | Yes | CC | N | 3 | UG |

CC, Coiled-coil; UG, Ungrouped; N, N-terminal; I, Internal; C, C-terminal.
