# Supplementary material for: Comprehensive comparative analysis of kinesins in photosynthetic eukaryotes
Source: BMC Genomics. 2006 Jan 31;7:18. doi: 10.1186/1471-2164-7-18 (PMC1434745; doi:10.1186/1471-2164-7-18)
Supplement: Additional file 6 — Supplemental Table 6. P. chryosporium kinesins and their structural features. [file 1471-2164-7-18-S6.pdf]

Supplemental Table 6 - *P. chryosporium* kinesins and their structural features

Gene ID	Protein length	EST	Additional Domains	MD location	# of exons	Family
genewise2nd.41.36.1	927	No	CC	N	15	1
genewise2nd.4.121.1	1285	No	CC, FHA	N	15	3
pc.3.10.1	527	No	CC	N	4	4
pc.41.67.1	821	No	CC	N	5	5
pc.11.256.1	611	No	CC	N	6	6
pc.95.48.1	421	No	CC	C	2	8
pc.13.63.1	354	No		C	7	13
genewise2nd.73.22.1	511	No		C	8	14

In gene tree figures, "gw" refers to “genwise2nd” prefix. CC, Coiled-coil; FHA, Fork head associated; N, N-terminal; I, Internal; C, C-terminal.
