# Supplementary material for: Comprehensive comparative analysis of kinesins in photosynthetic eukaryotes
Source: BMC Genomics. 2006 Jan 31;7:18. doi: 10.1186/1471-2164-7-18 (PMC1434745; doi:10.1186/1471-2164-7-18)
Supplement: Additional file 7 — Supplemental Table 7. P. sojae kinesins and their structural features. [file 1471-2164-7-18-S7.pdf]

Supplemental Table 7 - *P. sojae* kinesins and their structural features

Gene ID	Protein length	EST	Additional Domains	MD location	# of exons	Family
137039	1488	Yes	CC	I	4	1
130051	430	Yes		N	2	1
142980	878	Yes	CC	N	4	1
108563	714	Yes	CC	N	4	2
120306	674	Yes	CC	N	2	2
108377	384	Yes		N	11	3
128382	1665	Yes	PH	N	6	3
141078	1744	Yes	CC	N	3	3
142793	724	Yes	CC	N	1	3
142668	1513	Yes	CC	N	1	4
108445	398	Yes		ND	3	5
140636	628	Yes	CC	N	2	6
138580	835	Yes	CC	N	5	7
127973	1899	Yes	CC	N	1	7
128334	1129	Yes	CC	N	1	7
141959	612	Yes	CC	I	2	8
137042	856	Yes	CC	N	4	9
136418	679	Yes	PH	N	6	9
130522	1250	Yes	CC, 3' exoribonuclease	I	7	9
139555	875	Yes	CC	N	1	9
121181	369	Yes		ND	1	9
143005	1992	Yes	CC, Ankyrin repeat	N	4	12
141849	1195	Yes	CC	N	3	12
136489	610	Yes		N	8	13
135741	516	Yes		I	4	13
136872	1158	Yes	CC, CNB, TPR	N	3	13
142524	429	Yes		ND	1	13
142636	1145	Yes	CC	I	3	14
121412	365	Yes		ND	1	14
109123	348	Yes		ND	1	14
140964	801	Yes	CC	I	3	14
137761	705	Yes	CC	N	5	14
141193	2127	Yes	CC, MFS, TM domain	C	7	14
124499	355	Yes		ND	4	14
129079	1215	Yes	CC	C	1	14
121577	333	Yes	WD-40 repeat	ND	7	14
134623	901	Yes	CC	N	7	UG
137622	1709	Yes	CC, IPRP, C2	N	10	UG

142481	515	Yes	CC	N	2	UG
**130250**	1542	Yes	CC, WD-40 repeat	C	4	UG
**131621**	675	Yes	CC	N	4	UG
**132151**	954	Yes	CC	N	6	UG
**137485**	756	Yes	CC	I	2	UG

Sequences shown in bold correspond to the unresolved *P. sojae* block in Fig. 2. Possible kinesin with truncated motor domain: 136415. ND, Not determined; CC, Coiled-coil; MFS, Major facilitator superfamily; TM, Transmembrane domain; CNB, Cyclic nucleotide binding; TPR, Tetratricopeptide repeat; WD-40, A 40 amino acid repeat motif with W and D dipeptides at the terminus; C2, Protein kinase C conserved region 2; IPRP, Inositol polyphosphate related phosphatase; UG, Ungrouped, N, N-terminal; I, Internal; C, C-terminal.
