# Supplementary material for: Comprehensive comparative analysis of kinesins in photosynthetic eukaryotes
Source: BMC Genomics. 2006 Jan 31;7:18. doi: 10.1186/1471-2164-7-18 (PMC1434745; doi:10.1186/1471-2164-7-18)
Supplement: Additional file 8 — Supplemental Table 8. C. intestinalis kinesins and their structural features. [file 1471-2164-7-18-S8.pdf]

Supplemental Table 8 - *C. intestinalis* kinesins and their structural features

Gene ID	Protein length	EST	Additional Domains	MD location	# of exons	Family
ci0100143504	866	No	CC	N	20	1
ci0100134965	368	No		ND	8	2
ci0100133723	739	No	CC	N	16	2
ci0100148992	654	No	CC	N	14	2
ci0100136694	524	No	CC	N	12	3
ci0100137520	537	No	CC, FHA	N	12	3
ci0100134202	756	No	FHA	N	19	3
ci0100131206	1558	No	CC, FHA	N	32	3
ci0100143686	1249	No	CC, C2	N	27	3
ci0100130156	1322	No	CC, FHA	I	24	3
ci0100130413	1262	No	CC, WD-40 repeat	N	26	4
ci0100131275	1128	No	CC	N	24	4
ci0100154383	781	Yes	CC	N	18	6
ci0100130230	1424	No	CC	N	31	6
ci0100133471	466	No		N	13	7
ci0100145354	432	No		N	3	9
ci0100145013	792	No	CC	N	1	9
ci0100131135	605	No	CC	I	12	9
ci0100141905	493	No		N	1	10
ci0100130295	828	No	CC	N	19	12
ci0100146176	740	No	CC	I	17	13
ci0100151891	588	No	CC	C	11	14
ci0100131201	894	No	CC	N	2	14
ci0100133346	724	No		C	17	14
ci0100131488	658	No	CC	C	12	UG

Other possible kinesins with truncated motor domains: ci0100153316, ci0100134548, ci0100131508, ci0100131211. ND, Not determined; CC, Coiled-coil; FHA, Fork head associated; WD-40, A 40 amino acid repeat motif with W and D dipeptides at the terminus; C2, Protein kinase C conserved region; UG, Ungrouped; N, N-terminal; I, Internal, C, C-terminal.
