# Supplementary material for: Comprehensive comparative analysis of kinesins in photosynthetic eukaryotes
Source: BMC Genomics. 2006 Jan 31;7:18. doi: 10.1186/1471-2164-7-18 (PMC1434745; doi:10.1186/1471-2164-7-18)
Supplement: Additional file 9 — Supplemental Table 9. P. falciparum kinesins and their structural features. [file 1471-2164-7-18-S9.pdf]

Supplemental Table 9 - *P. falciparum* kinesins and their structural features

Gene ID	Protein length	Microarray	Additional Domains	MD location	# of exons	Family
PFC0770c	1619	Yes		N	2	5
PFL2190c	1605	Yes		N	1	5
PFL0545w	1844	Yes	CC	N	3	7
PFC0860w	1200	Yes		I	1	8
PFA0535c	1669	Yes	CC	I	6	8
PFL2165w	1351	Yes		N	2	13
MAL8P1.132	929	Yes	CC	N	5	UG
PF07_0104	1897	Yes	CC	N/I	1	UG
PF11_0478	735	Yes	CC	N	7	UG

CC, Coiled-coil; UG, Ungrouped; N, N-terminal; I, Internal; C, C-terminal.
