# Supplementary material for: Comprehensive comparative analysis of kinesins in photosynthetic eukaryotes
Source: BMC Genomics. 2006 Jan 31;7:18. doi: 10.1186/1471-2164-7-18 (PMC1434745; doi:10.1186/1471-2164-7-18)
Supplement: Additional file 10 — Supplemental Table 10. D. discoideum kinesins and their structural features. [file 1471-2164-7-18-S10.pdf]

Supplemental Table 10 - *D. discoideum* kinesins and their structural features

Gene ID	Protein length	cDNA	Additional Domains	MD location	# of exons	Family
DDB0166988	990	Yes	CC	N	4	1
DDB0204045	1193	Yes	CC	N	3	1
DDB0204609	1255	Yes	CC, TM	N	4	1
DDB0217523	1222	Yes	CC, TM	I	2	1
DDB0189215	2205	Yes	CC, FHA, PH	N	3	3
DDB0218612	1873	Yes	CC, WD-40 repeat	N	3	4
DDB0187903	1265	Yes[a]	CC	N	4	5
DDB0189854	1499	Yes[a]	CC	N	3	6
DDB0189194	685	Yes[a]	CC	N	1	7
DDB0186344	1922	Yes	CC	N	2	7
DDB0219839	1238	Yes	CC	N	1	8
DDB0216601	1030	Yes	CC	I	3	13
DDB0189377	792	Yes	CC	C	2	14

[a]Expression data is based on EST evidence only. CC, Coiled-coil; TM, Transmembrane domain; FHA, Fork head associated; PH, Pleckstrin homology; WD-40, A 40 amino acid repeat motif with W and D dipeptides at the terminus; N, N-terminal; I, Internal; C, C-terminal.
