# Supplementary material for: Comprehensive comparative analysis of kinesins in photosynthetic eukaryotes
Source: BMC Genomics. 2006 Jan 31;7:18. doi: 10.1186/1471-2164-7-18 (PMC1434745; doi:10.1186/1471-2164-7-18)
Supplement: Additional file 11 — Supplemental Table 11. L. major kinesins and their structural features. [file 1471-2164-7-18-S11.pdf]

Supplemental Table 11 - *L. major* kinesins and their structural features

Gene ID	Protein length	EST	Additional Domains	MD location	# of exons	Family
LmjF17.0800	938	No	CC	N	1	2
LmjF32.0680	1117	No	CC	N	1	2
LmjF34.4260	1548	No	CC	N	1	3
LmjF14.0810	1315	No	CC	N	1	3
LmjF14.1100	2765	No	CC	N	1	3
LmjF14.1120	2976	No	CC	N	1	3
LmjF14.1110	1229	No	CC	N	1	3
LmjF16.1460	1991	No	CC,	N	1	3
LmjF16.1470	807	No	CC	N	1	3
LmjF29.0970	1665	No	CC	N	1	3
LmjF19.0680	1061	No	CC	N	1	3
LmjF19.0690	1034	No	CC	N	1	3
LmjF19.0700	2221	No	CC	N	1	3
LmjF23.0560	656	No	CC, IQ Calmodulin binding region	N	1	3
LmjF21.1040	2121	No	CC	N	1	3
LmjF22.0960	1257	No	CC	N	1	3
LmjF29.2380	590	No	CC	N	1	3
LmjF29.2390	589	No	CC, C2	N	1	3
LmjF33.2560	2078	No		N	1	3
LmjF30.0350	607	No	CC	N	1	5
LmjF25.1970	1064	No	CC	N	1	7
LmjF13.0700	1435	No	CC	C	1	9
LmjF17.0160	1072	No	CC	N	1	9
LmjF05.0630	1227	No	CC	N	1	9
LmjF06.0180	672	No		N	1	9
LmjF34.1540	943	No	CC	N	1	9
LmjF36.5150	625	No	CC	N	1	10
LmjF01.0030	668	No		I	1	13
LmjF13.1610	728	No		I	1	13
LmjF24.0640	577	No		N	1	13
LmjF31.0290	840	No	CC	I	1	13
LmjF13.0130	729	No	Zn-finger TRAF	N	1	13
LmjF35.4700	480	No		I	1	13
LmjF19.0260	839	No	CC, VHS	C	1	14
LmjF24.1430	3275	No	CC	I	1	14
LmjF30.1450	1212	No	CC	N	1	14
LmjF28.1850	783	No	CC	C	1	14
LmjF31.2710	1084	No	CC, MORN repeat	C	1	UG
LmjF30.3060	1191	No	CC	N	1	UG
LmjF22.0560	891	No	CC	N	1	UG

LmjF20.0640	1261	No	CC	N/I	1	UG
LmjF18.1530	1242	No	CC	N	1	UG
LmjF33.2140	956	No	CC	N	1	UG
LmjF16.1580	1083	No		N	1	UG
LmjF35.2090	698	No	CC	N	1	UG
**LmjF05.0760**	1254	No		N	1	UG
**LmjF06.1030**	886	No	CC	N	1	UG
**LmjF09.0120**	1065	No	CC	N	1	UG
**LmjF09.0290**	1264	No		N	1	UG
**LmjF11.0870**	1417	No	CC	N	1	UG
**LmjF17.1110**	1430	No	CC, ARM, C2	N	1	UG
**LmjF18.1600**	796	No		N	1	UG
**LmjF25.1950**	1062	No		N	1	UG
**LmjF32.0420**	718	No	CC, MORN repeat	N	1	UG

Sequences shown in bold correspond to the unresolved *Leishmania* block in Fig. 2.
CC, Coiled-coil; ARM, Armadillo repeat; VHS, Domain present in Vps-27, Hrs, Stam; MORN, Membrane occupation and recognition nexus; C2, Protein kinase C conserved region 2; UG, Ungrouped; N, N-terminal; I, Internal; C, C-terminal.
