# Supplementary material for: Comprehensive comparative analysis of kinesins in photosynthetic eukaryotes
Source: BMC Genomics. 2006 Jan 31;7:18. doi: 10.1186/1471-2164-7-18 (PMC1434745; doi:10.1186/1471-2164-7-18)
Supplement: Additional file 19 — Supplemental Fig 19. Unrooted parsimony jackknife tree inferred from both amino acid and gap characters. [file 1471-2164-7-18-S19.pdf]

Supplemental Figure 19. Unrooted parsimony jackknife tree inferred from both amino acid and gap characters

```
Parsimony motor domain AA + SIC 50% majority-rule consensus of 1000
trees (all trees equally weighted)
```

```
/-------------------------------------------------------- AT 1G01950(1)
|
|                                                   /---- AT 1G09170(2)
|                                          /---56---+
|                                          |        \---- AT 5G27000(51)
|                                          |
|                                          |        /---- AT 2G47500(20)
|                                          |   /-94-+
|                                          |   |    \---- PT 00140705(404)
|                                          +83-+
|                                          |   |    /---- OS SBCC010387(307)
|                             /-----62-----+   \100-+
|                             |            |        \---- OS IBCD009838(348)
|                             |            |
|                             |            |        /---- OS SBCC037918(331)
|                             |            +--100---+
|                             |            |        \---- OS IBCD035259(377)
|                             |            |
|                             |            \------------- PT 01330083(410)
|                             |
|                             |                     /---- AT 1G18410(5)
|                             |                /100-+
|                             |                |    \---- AT 1G73860(13)
|                             |            /66-+
|                             |            |   |    /---- PT 00120480(401)
|                             |            |   \100-+
|                             |            |        \---- PT 00150291(405)
|                         /62-+            |
|                         |   |            |        /---- AT 1G63640(11)
|                         |   |            |   /100-+
|                         |   |        /67-+   |    \---- AT 5G41310(54)
|                         |   |        |   +73-+
|                         |   |        |   |   \--------- PT LG III001053(420)
|                         |   |        |   |
|                         |   |        |   |        /---- OS SBCC022107(321)
|                         |   |        |   |   /100-+
|                         |   |   /-95-+   |   |    \---- OS IBCD021045(364)
|                         |   |   |    |   \100+
|                         |   |   |    |       \--------- OS IBCD005609(340)
|                     /96-+   |   |    |
|                     |   |   |   |    |            /---- OS SBCC003463(298)
|                     |   |   |   |    \----100-----+
|                     |   |   \63-+                 \---- OS IBCD003270(338)
|                     |   |       |
|                     |   |       |                 /---- AT 3G44730(32)
|                     |   |       |            /-99-+
|                     |   |       |            |    \---- PT 1400045(384)
|                /-88-+   |       \-----94-----+
|                |    |   |                    \--------- OS IBCD014642(355)
|                |    |   |
|                |    |   |                         /---- AT 3G10310(22)
|                |    |   \-----------100-----------+
|                |    |                             \---- PT 00060440(397)
|                |    |
|                |    \---------------------------------- CR C 510041(128)
|                |
|                |                                  /---- AT 1G18370(4)
|                |                             /100-+
|                |                             |    \---- PT LG XII000185(434)
|                |                         /88-+
|                |                         |   |    /---- OS SBCC002024(295)
|                |                         |   \100-+
|                |                /---88---+        \---- OS IBCD001985(336)
|                |                |        |
|                |                |        |        /---- AT 3G43210(30)
|                |                |        \---66---+
|                |                |                 \---- PT SCAFFOLD 2800(428)
|                |                |
|                |                |                 /---- AT 2G21300(14)
|                |                |            /100-+
|                |                |            |    \---- AT 4G38950(45)
|                |                |        /100+
```

```
|                |                |        |   \--------- PT LG IX0409(388)
|                |                |    /77-+
|                |            /100+    |   |        /---- OS SBCC007775(300)
|                |            |   |    |   \--100---+
|                |            |   |    |            \---- OS IBCD007402(341)
|                |            |   |    |
|                |            |   |    |            /---- AT 3G51150(36)
|                |            |   |    |       /100-+
|                |            |   |    |       |    \---- AT 5G66310(61)
|                |            |   |    +--98---+
|                |            |   |    |       \--------- PT 570109(385)
|                |            |   |    |
|                |            |   |    |            /---- AT 4G24170(43)
|                |            |   \100-+----100-----+
|                +-----54-----+        |            \---- AT 5G42490(55)
|                |            |        |
|                |            |        |            /---- OS SBCC028926(324)
|                |            |        |       /100-+
|                |            |        |       |    \---- OS IBCD027313(369)
|                |            |        +--98---+
|                |            |        |       |    /---- OS SBCC030982(329)
|                |            |        |       \100-+
|                |            |        |            \---- OS IBCD029182(373)
|                |            |        |
|                |            |        \----------------- PT LG II000243(418)
|                |            |
|                |            \-------------------------- CR C 1480016(119)
|                |
|                |                                  /---- AT 1G18550(6)
|                |                             /-74-+
|                |                             |    \---- PT 00120495(402)
|                +-------------100-------------+
|                |                             |    /---- OS SBCC002630(296)
|                |                             \100-+
|                |                                  \---- OS IBCD002607(337)
|                |
|                |                                  /---- AT 1G20060(7)
|                |                             /-59-+
|                |                             |    \---- PT LG XIII0655(392)
|                +-------------100-------------+
|                |                             |    /---- OS SBCC004978(299)
|                |                             \100-+
|                |                                  \---- OS IBCD004715(339)
|                |
|                |                             /--------- AT 1G21730(8)
|                |                             |
|                |                             |    /---- OS SBCC015147(312)
|                |                     /--68---+100-+
|                |                     |       |    \---- OS IBCD014393(354)
|                |                     |       |
|                |                     |       \--------- PT 00020745(395)
|                |                     |
|                |                /-91-+            /---- AT 3G12020(23)
|                |                |    |       /-83-+
|                |                |    |       |    \---- AT 5G06670(48)
|                |                |    |   /93-+
|                |                |    |   |   \--------- PT 00160532(407)
|                |                |    \92-+
|                |                |        |        /---- OS SBCC008574(302)
|                |            /100+        \---98---+
|                |            |   |                 \---- OS IBCD008139(343)
|                |            |   |
|                |            |   |                 /---- AT 2G21380(15)
|                |            |   |            /-67-+
|                |            |   |            |    \---- AT 4G39050(46)
|                +-----52-----+   \----100-----+
|                |            |                |    /---- OS IBCD031186(374)
|                |            |                \-59-+
|                |            |                     \---- PT LG IV1283(387)
|                |            |
|                |            \-------------------------- DD B0189194(65)
|                |
|                |                                  /---- AT 1G55550(9)
|                |                             /100-+
|                |                             |    \---- PT LG I000011(415)
|                |                     /--96---+
|                |                     |       |    /---- OS SBCC001075(294)
|                |                     |       \100-+
|                |                     |            \---- OS IBCD001052(335)
|                |                     |
|                +---------100---------+            /---- AT 5G27950(53)
```

```
|                |                    |   /--100---+
|                |                    |   |        \---- PT LG XIII000327(426)
|                |                    |   |
|                |                    \100+        /---- OS SBCC029770(327)
|                |                        |   /-94-+
|                |                        |   |    \---- OS IBCD025572(368)
|                |                        \100+
|                |                            \--------- OS IBCD025570(367)
|                |
|                |                                 /---- AT 1G59540(10)
|                |                            /-98-+
|                |                            |    \---- PT LG XIV000016(435)
|                |                        /100+
|                |                        |   |    /---- OS SBCC035459(330)
|                |                        |   \100-+
|                |                        |        \---- OS IBCD033171(376)
|                |                    /94-+
|                |                    |   |        /---- AT 3G10180(21)
|                |                    |   |   /100-+
|                |                    |   |   |    \---- PT LG VI0681(389)
|                |                    |   \100+
|                +---------51----------+       |    /---- OS SBCC030333(328)
|                |                    |       \100-+
|                |                    |            \---- OS IBCD028585(372)
|                |                    |
|                |                    \----------------- DD B0186344(63)
|                |
|                |                            /--------- AT 1G72250(12)
|                |                            |
|                |                        /51-+    /---- PT 00111158(400)
|                |                        |   \100-+
|                |                        |        \---- PT LG I003040(417)
|                |                    /98-+
|                |                    |   |        /---- OS SBCC038356(333)
|                |                    |   \--100---+
|                |                    |            \---- OS IBCD035672(379)
|                |               /-90-+
|                |               |    |            /---- AT 2G22610(16)
|                |               |    |       /-67-+
|                |               |    |       |    \---- PT LG II001016(419)
|                |               |    \--100--+
|                |               |            |    /---- OS SBCC009123(304)
|                |               |            \100-+
|                |               |                 \---- OS IBCD008627(345)
|                +------100-------+
|                |               |        /------------- AT 5G27550(52)
|                |               |        |
|                |               |    /81-+   /--------- PT 00130189(403)
|                |               |    |   |   |
|                |               |    |   \100+    /---- PT 02560013(413)
|                |               |    |       \100-+
|                |               \-99-+            \---- PT 07000001(414)
|                |                    |
|                |                    |            /---- OS SBCC018779(317)
|                |                    \----100-----+
|                |                                 \---- OS IBCD017918(360)
|                |
|                |                                 /---- AT 2G28620(17)
|                |                                 |
|                |                                 +---- AT 3G45850(33)
|                |                            /-73-+
|                |                            |    +---- PT 00160756(408)
|                |                            |    |
|                |                    /--82---+    \---- PT LG I001798(416)
|                |                    |       |
|                |                    |       |    /---- OS SBCC016982(315)
|                |                    |       \100-+
|                |                    |            \---- OS IBCD016250(358)
|                |               /-64-+
|                |               |    |   /------------- AT 2G37420(19)
|                |               |    |   |
|                |               |    |   |        /---- OS SBCC010233(306)
|                |               |    \90-+   /100-+
|                |               |        |   |    \---- OS IBCD009684(347)
|                |           /83-+        \77-+
|                |           |   |            \--------- PT 00060694(398)
|                |           |   |
|                |           |   |                 /---- AT 2G36200(18)
|                |           |   |            /-90-+
|                |           |   |            |    \---- PT LG XVI0226(393)
|                |       /100+   \----100-----+
```

```
|                |        |   |                |    /---- OS SBCC028993(325)
|                |        |   |                \100-+
|                |        |   |                     \---- OS IBCD027399(370)
|                |        |   |
|                |        |   \-------------------------- CR C 530027(129)
|                |        |
|                |        |                         /---- DD B0187903(64)
|                |        |                         |
|                |        +-----------73------------+---- GL 16425(148)
|                |        |                         |
|                |        |                         \---- PF C0770C(513)
|                |        |
|                |        |                         /---- CE F23B12.8(99)
|                |    /81-+                         |
|                |    |   +-----------57------------+---- DM CG9191 PA(209)
|                |    |   |                         |
|                |    |   |                         \---- HS 1706622(224)
|                |    |   |
|                |    |   +------------------------------ PS 108445(252)
|                |    |   |
|                |    |   +------------------------------ CM C157C(490)
|                |    |   |
|                +-60-+   |                         /---- SC CIN8(495)
|                |    |   |                    /-77-+
|                |    |   |                    |    \---- SC KIP1(497)
|                |    |   |                /72-+
|                |    |   |                |   \--------- SP AC25G10.07C(520)
|                |    |   \-------87-------+
|                |    |                    \------------- PC PC.41.67.1(507)
|                |    |
|                |    \---------------------------------- TP 135880(87)
|                |
|                |                                   /---- AT 3G16060(24)
|                |                     /-----99-----+
|                |                     |            \---- OS SBCC002748(297)
|                |                     |
|                |                     |       /--------- AT 3G16630(25)
|                |                /100-+       |
|                |                |    |   /91-+    /---- PT 00100152(399)
|                |                |    |   |   \-64-+
|                |                |    |   |        \---- PT 02100029(411)
|                |                |    \99-+
|                |                |        |        /---- OS SBCC017229(316)
|                |                |        \--100---+
|                |                |                 \---- OS IBCD016493(359)
|                |                |
|                |                +---------------------- DD B0216601(71)
|                |                |
|                |                |                 /---- TP 107522(76)
|                |                |                 |
|                |                +-------60--------+---- LM F35.4700(488)
|                |                |                 |
|                |                |                 \---- PC PC.13.63.1(505)
|                |                |
|                |                |                 /---- TP 112325(78)
|                |                +-------99--------+
|                |                |                 \---- PS 136489(269)
|                |                |
|                |                +---------------------- TP 116492(79)
|                |                |
|                |                |    /----------------- CE K11D9.1A(100)
|                |                |    |
|                |                |    |       /--------- CI 0100146176(182)
|                |                |    |       |
|                |                |    |   /52-+    /---- HS 3024057(229)
|                |                +-98-+   |   \-97-+
|                |                |    |   |        \---- HS 49355831(243)
|                +-------77-------+    |   |
|                |                |    |   |        /---- DM CG12192 PA(189)
|                |                |    \56-+   /-94-+
|                |                |        |   |    \---- DM CG3219 PA(198)
|                |                |        +99-+
|                |                |        |   \--------- DM CG1453 PA(192)
|                |                |        |
|                |                |        \------------- HS 1695882(223)
|                |                |
|                |                +---------------------- CR C 730040(135)
|                |                |
|                |                +---------------------- GL 16945(151)
|                |                |
|                |                +---------------------- PS 135741(267)
```

```
|                |                |
|                |                +---------------------- PS 136872(270)
|                |                |
|                |                +---------------------- PS 142524(285)
|                |                |
|                |                |                 /---- LM F01.0030(436)
|                |                +-------61--------+
|                |                |                 \---- LM F13.1610(446)
|                |                |
|                |                +---------------------- LM F13.0130(444)
|                |                |
|                |                +---------------------- LM F24.0640(468)
|                |                |
|                |                +---------------------- LM F31.0290(479)
|                |                |
|                |                \---------------------- PF L2165W(516)
|                |
|                |                         /------------- AT 3G17360(26)
|                |                         |
|                |                         |        /---- AT 3G19050(27)
|                |                     /96-+   /-96-+
|                |                     |   |   |    \---- PT 01300020(409)
|                |                     |   \88-+
|                |                     |       |    /---- OS SBCC038188(332)
|                |                /100-+       \100-+
|                |                |    |            \---- OS IBCD035525(378)
|                |                |    |
|                |                |    |            /---- AT 3G44050(31)
|                +-------58-------+    \----100-----+
|                |                |                 \---- PT LG IX000131(421)
|                |                |
|                |                \---------------------- CR C 1350007(118)
|                |
|                |                                  /---- AT 3G20150(28)
|                |                             /-75-+
|                |                             |    \---- PT 00012593(394)
|                |                     /--100--+
|                |                     |       |    /---- OS SBCC011576(308)
|                |                     |       \100-+
|                |                     |            \---- OS IBCD010981(349)
|                |                     |
|                |                     |            /---- AT 3G23670(29)
|                +---------100---------+       /100-+
|                |                     |       |    \---- AT 4G14150(40)
|                |                     |   /98-+
|                |                     |   |   |    /---- PT LG XIV000891(427)
|                |                     |   |   \-99-+
|                |                     \100+        \---- PT LG II001048(431)
|                |                         |
|                |                         |        /---- OS SBCC014640(310)
|                |                         \--100---+
|                |                                  \---- OS IBCD013910(352)
|                |
|                |                             /--------- AT 3G49650(34)
|                |                             |
|                |                         /100+    /---- OS SBCC012732(309)
|                |                         |   \100-+
|                +-----------100-----------+        \---- OS IBCD012108(350)
|                |                         |
|                |                         \------------- CR C 1310024(117)
|                |
|                |                     /----------------- AT 3G50240(35)
|                |                     |
|                |                     |       /--------- AT 5G47820(56)
|                |                     |       |
|                |                /100-+       |    /---- OS SBCC029113(326)
|                |                |    |   /54-+100-+
|                |                |    |   |   |    \---- OS IBCD027529(371)
|                |                |    |   |   |
|                |                |    \54-+   \--------- PT LG II000522(429)
|                |                |        |
|                |                |        \------------- PT LG VII000272(432)
|                |            /96-+
|                |            |   |            /--------- AT 5G60930(58)
|                |            |   |            |
|                |            |   |        /91-+    /---- PT 00020976(396)
|                |            |   |        |   \100-+
|                |            |   |        |        \---- PT 02310007(412)
|                +-----75-----+   \--100---+
|                |            |            |        /---- OS SBCC008366(301)
|                |            |            \--100---+
```

```
|                |            |                     \---- OS IBCD007936(342)
|                |            |
|                |            \-------------------------- CR C 120157(116)
|                |
|                |                             /--------- AT 3G63480(38)
|                |                             |
|                +-------------100-------------+    /---- OS SBCC026309(323)
|                |                             \100-+
|                |                                  \---- OS IBCD024826(366)
|                |
|                |                                  /---- AT 4G05190(39)
|                |                             /-99-+
|                |                             |    \---- AT 4G21270(42)
|                |                     /--76---+
|                |                     |       \--------- PT LG XI0089(390)
|                |                     |
|                |                     |            /---- AT 4G27180(44)
|                |                     |       /100-+
|                |                     |       |    \---- AT 5G54670(57)
|                |                     +--64---+
|                |                     |       \--------- PT LG XI000300(433)
|                |                     |
|                |                     |            /---- OS SBCC016426(313)
|                |                /-98-+----100-----+
|                |                |    |            \---- OS IBCD015695(356)
|                |                |    |
|                |                |    |            /---- OS SBCC023227(322)
|                |                |    |       /-89-+
|                |                |    |       |    \---- OS IBCD022126(365)
|                |                |    |   /70-+
|                +-------76-------+    |   |   |    /---- OS SBSC051135(334)
|                |                |    |   |   \100-+
|                |                |    \100+        \---- OS IBCD012736(351)
|                |                |        |
|                |                |        \------------- OS IBCD031252(375)
|                |                |
|                |                \---------------------- CR C 60218(131)
|                |
|                |                             /--------- AT 4G14330(41)
|                |                             |
|                |                         /98-+    /---- PT LG VIII001511(424)
|                |                         |   \-99-+
|                |                         |        \---- PT LG X000373(425)
|                +-----------100-----------+
|                |                         |        /---- OS SBCC008809(303)
|                |                         \--100---+
|                |                                  \---- OS IBCD008347(344)
|                |
|                |                             /--------- AT 5G02370(47)
|                |                             |
|                |                         /100+    /---- OS SBCC019161(318)
|                |                         |   \100-+
|                |                         |        \---- OS IBCD018270(361)
|                |                         |
|                |                     /100+        /---- AT 5G23910(50)
|                |                     |   |   /-92-+
|                |                     |   |   |    \---- PT 00151235(406)
|                |                     |   \96-+
|                |                     |       |    /---- OS SBCC014774(311)
|                +---------73----------+       \100-+
|                |                     |            \---- OS IBCD014035(353)
|                |                     |
|                |                     |       /--------- CI 0100141905(177)
|                |                     |       |
|                |                     \--100--+    /---- HS 4519443(241)
|                |                             \100-+
|                |                                  \---- MM 21704182(380)
|                |
|                |                                  /---- AT 5G10470(49)
|                |                             /100-+
|                |                             |    \---- AT 5G65460(59)
|                |                         /82-+
|                |                         |   \--------- PT LG VII001210(422)
|                +-----------100-----------+
|                |                         |        /---- OS SBCC020793(320)
|                |                         \--100---+
|                |                                  \---- OS IBCD019841(363)
|                |
|                |                             /--------- AT 5G65930(60)
|                |                             |
|                |                         /65-+    /---- OS SBCC016659(314)
```

```
|                |                         |   \100-+
|                |                     /100+        \---- OS IBCD015937(357)
|                |                     |   |
|                |                /-98-+   \------------- PT LG XI1218(391)
|                |                |    |
|                |                |    \----------------- CR C 1030008(114)
|                |            /53-+
|                |            |   +---------------------- TP 12124(81)
|                |            |   |
|                +-----96-----+   \---------------------- CI 0100131201(166)
|                |            |
|                |            \-------------------------- PS 141193(281)
|                |
|                +--------------------------------------- DD B0166988(62)
|                |
|                |                /---------------------- DD B0189215(66)
|                |                |
|                |                |            /--------- CE R144.1(105)
|                |                |            |
|                +-------60-------+        /90-+    /---- CI 0100136694(175)
|                |                |        |   \-91-+
|                |                |    /98-+        \---- CI 0100137520(176)
|                |                |    |   |
|                |                \-88-+   \------------- CI 0100134202(173)
|                |                     |
|                |                     \----------------- PS 108377(251)
|                |
|                +--------------------------------------- DD B0189377(67)
|                |
|                +--------------------------------------- DD B0189854(68)
|                |
|                +--------------------------------------- DD B0204045(69)
|                |
|                +--------------------------------------- DD B0204609(70)
|                |
|                +--------------------------------------- DD B0217523(72)
|                |
|                +--------------------------------------- DD B0218612(73)
|                |
|                |                                  /---- DD B0219839(74)
|                +----------------58----------------+
|                |                                  \---- GL 4371(154)
|                |
|                +--------------------------------------- TP 105812(75)
|                |
|                |                                  /---- TP 110182(77)
|                +---------------100----------------+
|                |                                  \---- TP 156428(92)
|                |
|                +--------------------------------------- TP 119229(80)
|                |
|                |                                  /---- TP 121289(82)
|                +---------------100----------------+
|                |                                  \---- TP 163717(93)
|                |
|                +--------------------------------------- TP 122963(83)
|                |
|                +--------------------------------------- TP 123312(84)
|                |
|                |                             /--------- TP 124560(85)
|                |                             |
|                |                         /51-+    /---- TP 151754(90)
|                |                         |   \-59-+
|                +-----------92------------+        \---- PS 124499(291)
|                |                         |
|                |                         \------------- PS 129079(260)
|            /100+
|            |   |                                  /---- TP 132625(86)
|            |   |                                  |
|            |   +----------------51----------------+---- PS 141849(282)
|            |   |                                  |
|            |   |                                  \---- PS 143005(290)
|            |   |
|            |   +--------------------------------------- TP 137385(88)
|            |   |
|            |   |                                  /---- TP 142737(89)
|            |   |                                  |
|            |   |                /-------74--------+---- CR C 250150(122)
|            |   |                |                 |
|            |   |                |                 \---- PS 137042(272)
|            |   |                |
```

```
|            |   |                 |        /------------- CR C 50080(127)
|            |   |                 |        |
|            |   |                 |        |   /--------- CI 0100145013(180)
|            |   |                 |        |   |
|            |   |                 |        +78-+    /---- HS 11275982(219)
|            |   |                 |    /57-+   \100-+
|            |   |                 |    |   |        \---- MM 5295882(383)
|            |   |                 |    |   |
|            |   +--------65-------+    |   +------------- PS 130522(263)
|            |   |                 +-65-+   |
|            |   |                 |    |   \------------- PS 139555(277)
|            |   |                 |    |
|            |   |                 |    \----------------- GL 6404(158)
|            |   |                 |
|            |   |                 +---------------------- GL 10137(139)
|            |   |                 |
|            |   |                 +---------------------- CI 0100145354(181)
|            |   |                 |
|            |   |                 +---------------------- PS 136418(268)
|            |   |                 |
|            |   |                 \---------------------- LM F05.0630(437)
|            |   |
|            |   +---------------------------------------- TP 155696(91)
|            |   |
|            |   |                                   /---- TP 133437(94)
|            |   +-----------------51----------------+
|            |   |                                   \---- PS 137039(271)
|            |   |
|            |   +---------------------------------------- CE 01083(95)
|            |   |
|            |   |                                   /---- CE C06G3.2(96)
|            |   |                          /--100---+
|            |   |                          |        \---- CE C33H5.4A(97)
|            |   |                          |
|            |   +------------53------------+   /--------- CI 0100130295(163)
|            |   |                          |   |
|            |   |                          \79-+    /---- HS 9910266(250)
|            |   |                              \100-+
|            |   |                                   \---- MM 40644653(381)
|            |   |
|            |   |                                   /---- CE C41G7.2(98)
|            |   |                              /100-+
|            |   |                              |    \---- CE M01E11.6(102)
|            |   +--------------100-------------+
|            |   |                              \--------- CE W02B12.7(108)
|            |   |
|            |   |                                   /---- CE M02B7.3A(101)
|            |   |                                   |
|            |   |                              /-74-+---- CI 0100134965(174)
|            |   |                              |    |
|            |   |                      /--78---+    \---- HS HCP48743(217)
|            |   |                      |       |
|            |   |                      |       \--------- DM CG17461 PA(196)
|            |   |                      |
|            |   |                      |   /------------- CE F20C5.2B(111)
|            |   |                      |   |
|            |   |                      +70-+   /--------- CI 0100133723(172)
|            |   |                      |   |   |
|            |   |                      |   \72-+    /---- HS 3913957(233)
|            |   |                      |       \-59-+
|            |   |                      |            \---- HS 3913958(234)
|            |   |                      |
|            |   |                      +----------------- CR C 160226(120)
|            |   |                 /-63-+
|            |   |                 |    +----------------- CR C 1880008(121)
|            |   |                 |    |
|            |   |                 |    |            /---- CI 0100148992(183)
|            |   |                 |    |       /-99-+
|            |   |                 |    |       |    \---- HS 3851492(232)
|            |   |                 |    +--97---+
|            |   |                 |    |       \--------- DM CG10642 PA(186)
|            |   +--------75-------+    |
|            |   |                 |    +----------------- DM CG7293 PA(203)
|            |   |                 |    |
|            |   |                 |    |            /---- PS 108563(253)
|            |   |                 |    \----100-----+
|            |   |                 |                 \---- PS 120306(255)
|            |   |                 |
|            |   |                 |                 /---- GL 16456(149)
|            |   |                 \-------85--------+
|            |   |                                   \---- GL 17333(153)
```

```
|            |   |
|            |   |                     /----------------- CE M03D4.1A(103)
|            |   |                     |
|            |   |                     |       /--------- CI 0100154383(185)
|            |   +---------100---------+       |
|            |   |                     |   /80-+    /---- HS HCP49543(218)
|            |   |                     |   |   \100-+
|            |   |                     \84-+        \---- HS 400264(238)
|            |   |                         |
|            |   |                         \------------- DM CG1258 PA(191)
|            |   |
|            |   |            /------------------------- CE R05D3.7(104)
|            |   |            |
|            |   |            |            /------------- CI 0100143504(178)
|            |   |            |            |
|            |   |            |            |        /---- HS 2497520(226)
|            |   +----100-----+        /92-+   /-97-+
|            |   |            |        |   |   |    \---- HS 304358(230)
|            |   |            |        |   \58-+
|            |   |            |   /-86-+       \--------- HS 417216(240)
|            |   |            |   |    |
|            |   |            \81-+    \----------------- SPU 47550911(527)
|            |   |                |
|            |   |                \---------------------- DM CG7765 PA(204)
|            |   |
|            |   |                             /--------- CE T01G1.1A(106)
|            |   |                             |
|            |   |                             +--------- CI 0100130413(164)
|            |   |                             |
|            |   +-------------94--------------+--------- DM CG5300 PA(200)
|            |   |                             |
|            |   |                             |    /---- HS HCP1631302(211)
|            |   |                             \-88-+
|            |   |                                  \---- HS 5360129(244)
|            |   |
|            |   |                     /----------------- CE UNC 104(107)
|            |   |                     |
|            |   +---------100---------+   /------------- CI 0100131206(167)
|            |   |                     |   |
|            |   |                     \56-+   /--------- DM CG8566 PD(207)
|            |   |                         |   |
|            |   |                         \58-+    /---- HS 2497523(227)
|            |   |                             \-76-+
|            |   |                                  \---- HS 3913961(235)
|            |   |
|            |   +--------------------------------------- CE Y43F4B.6(109)
|            |   |
|            |   |                /---------------------- CE F56E3.3(110)
|            |   |                |
|            |   |                |        /------------- CI 0100143686(179)
|            |   |                |        |
|            |   +-------99-------+        |        /---- HS HCP34632(212)
|            |   |                |    /76-+   /100-+
|            |   |                |    |   |   |    \---- HS 8896164(249)
|            |   |                |    |   \77-+
|            |   |                \-73-+       \--------- HS 11761611(220)
|            |   |                     |
|            |   |                     \----------------- DM CG8183 PA(206)
|            |   |
|            |   +--------------------------------------- CE 7499692(112)
|            |   |
|            |   |                     /----------------- CE K12F2.2A(113)
|            |   |                     |
|            |   +---------100---------+   /------------- DM CG14535 PA(193)
|            |   |                     |   |
|            |   |                     \94-+   /--------- HS 29732867(228)
|            |   |                         |   |
|            |   |                         \99-+    /---- HS 6330751(246)
|            |   |                             \100-+
|            |   |                                  \---- MM 51767056(382)
|            |   |
|            |   +--------------------------------------- CR C 120136(115)
|            |   |
|            |   |                                  /---- CR C 260066(123)
|            |   +----------------94----------------+
|            |   |                                  \---- LM F06.0180(439)
|            |   |
|            |   +--------------------------------------- CR C 260133(124)
|            |   |
|            |   +--------------------------------------- CR C 340020(125)
|            |   |
```

```
|            |   +--------------------------------------- CR C 460075(126)
|            |   |
|            |   +--------------------------------------- CR C 570090(130)
|            |   |
|            |   |                                  /---- CR C 620070(132)
|            |   +----------------91----------------+
|            |   |                                  \---- LM F34.1540(485)
|            |   |
|            |   |                             /--------- CR C 670066(133)
|            |   |                             |
|            |   |                             +--------- PS 109123(254)
|            |   +-------------54--------------+
|            |   |                             |    /---- PS 121412(256)
|            |   |                             \-69-+
|            |   |                                  \---- LM F19.0260(459)
|            |   |
|            |   |                                  /---- CR C 710026(134)
|            |   |                             /-53-+
|            |   |                             |    \---- PS 128334(258)
|            |   +-------------99--------------+
|            |   |                             \--------- LM F25.1970(471)
|            |   |
|            |   +--------------------------------------- CR C 790050(136)
|            |   |
|            |   |                                  /---- GL 7874(137)
|            |   +----------------60----------------+
|            |   |                                  \---- GL 16161(146)
|            |   |
|            |   +--------------------------------------- GL 8886(138)
|            |   |
|            |   +--------------------------------------- GL 11442(140)
|            |   |
|            |   +--------------------------------------- GL 13797(141)
|            |   |
|            |   +--------------------------------------- GL 13825(142)
|            |   |
|            |   +--------------------------------------- GL 14070(143)
|            |   |
|            |   +--------------------------------------- GL 15134(144)
|            |   |
|            |   +--------------------------------------- GL 15962(145)
|            |   |
|            |   +--------------------------------------- GL 16224(147)
|            |   |
|            |   +--------------------------------------- GL 16650(150)
|            |   |
|            |   +--------------------------------------- GL 17264(152)
|            |   |
|            |   |                                  /---- GL 102101(155)
|            |   |                             /100-+
|            |   |                             |    \---- GL 112846(160)
|            |   +-------------100-------------+
|            |   |                             \--------- GL 6262(157)
|            |   |
|            |   +--------------------------------------- GL 102455(156)
|            |   |
|            |   +--------------------------------------- GL 112729(159)
|            |   |
|            |   |                                  /---- CI 0100130156(161)
|            |   |                             /-84-+
|            |   |                             |    \---- HS 452517(242)
|            |   +-------------88--------------+
|            |   |                             \--------- DM CG10718 PA(187)
|            |   |
|            |   |                                  /---- CI 0100130230(162)
|            |   |                                  |
|            |   +----------------90----------------+---- HS 3978240(236)
|            |   |                                  |
|            |   |                                  \---- HS 5911999(245)
|            |   |
|            |   |                                  /---- CI 0100131135(165)
|            |   |                             /-75-+
|            |   |                             |    \---- DM CG15844 PA(194)
|        /99-+   +-------------96--------------+
|        |   |   |                             \--------- HS HCP45833(216)
|        |   |   |
|        |   |   |                                  /---- CI 0100131275(168)
|        |   |   +----------------69----------------+
|        |   |   |                                  \---- HS 7266951(248)
|        |   |   |
|        |   |   +--------------------------------------- CI 0100131488(169)
```

```
|        |   |   |
|        |   |   +--------------------------------------- CI 0100133346(170)
|        |   |   |
|        |   |   +--------------------------------------- CI 0100133471(171)
|        |   |   |
|        |   |   |                                  /---- CI 0100151891(184)
|        |   |   +----------------93----------------+
|        |   |   |                                  \---- HS 4115553(239)
|        |   |   |
|        |   |   |                             /--------- DM CG10923 PA(188)
|        |   |   |                             |
|        |   |   +-------------96--------------+    /---- HS HCP43728(214)
|        |   |   |                             \-85-+
|        |   |   |                                  \---- HS 12053149(221)
|        |   |   |
|        |   |   +--------------------------------------- DM CG12298 PA(190)
|        |   |   |
|        |   |   +--------------------------------------- DM CG1708 PA(195)
|        |   |   |
|        |   |   +--------------------------------------- DM CG1763 PA(197)
|        |   |   |
|        |   |   |                                  /---- DM CG32955 PE(199)
|        |   |   +---------------100----------------+
|        |   |   |                                  \---- DM CG6392 PA(202)
|        |   |   |
|        |   |   |                                  /---- DM CG5658 PA(201)
|        |   |   +----------------99----------------+
|        |   |   |                                  \---- HS 6522736(247)
|        |   |   |
|        |   |   +--------------------------------------- DM CG7831 PA(205)
|        |   |   |
|        |   |   +--------------------------------------- DM CG8590 PA(208)
|        |   |   |
|        |   |   +--------------------------------------- DM CG9913 PA(210)
|        |   |   |
|        |   |   +--------------------------------------- HS HCP41593(213)
|        |   |   |
|        |   |   +--------------------------------------- HS HCP44045(215)
|        |   |   |
|        |   |   +--------------------------------------- HS 12654739(222)
|        |   |   |
|        |   |   +--------------------------------------- HS 23397458(225)
|        |   |   |
|        |   |   +--------------------------------------- HS 3702453(231)
|        |   |   |
|        |   |   +--------------------------------------- HS 399227(237)
|        |   |   |
|        |   |   +--------------------------------------- PS 127973(257)
|        |   |   |
|        |   |   +--------------------------------------- PS 128382(259)
|        |   |   |
|        |   |   +--------------------------------------- PS 130051(261)
|        |   |   |
|        |   |   +--------------------------------------- PS 130250(262)
|        |   |   |
|        |   |   +--------------------------------------- PS 131621(264)
|        |   |   |
|        |   |   +--------------------------------------- PS 132151(265)
|        |   |   |
|        |   |   +--------------------------------------- PS 134623(266)
|        |   |   |
|        |   |   |                                  /---- PS 137485(273)
|        |   |   +----------------88----------------+
|        |   |   |                                  \---- PS 142481(284)
|        |   |   |
|        |   |   +--------------------------------------- PS 137622(274)
|        |   |   |
|        |   |   +--------------------------------------- PS 137761(275)
|        |   |   |
|        |   |   +--------------------------------------- PS 138580(276)
|        |   |   |
|        |   |   +--------------------------------------- PS 140636(278)
|        |   |   |
|        |   |   +--------------------------------------- PS 140964(279)
|        |   |   |
|        |   |   |                                  /---- PS 141078(280)
|        |   |   +----------------58----------------+
|        |   |   |                                  \---- PS 142793(288)
|        |   |   |
|        |   |   +--------------------------------------- PS 141959(283)
|        |   |   |
```

```
|        |   |   +--------------------------------------- PS 142636(286)
|        |   |   |
|        |   |   +--------------------------------------- PS 142668(287)
|        |   |   |
|        |   |   +--------------------------------------- PS 142980(289)
|        |   |   |
|        |   |   +--------------------------------------- PS 121577(292)
|        |   |   |
|        |   |   +--------------------------------------- PS 121181(293)
|        |   |   |
|        |   |   +--------------------------------------- LM F05.0760(438)
|        |   |   |
|        |   |   |                                  /---- LM F06.1030(440)
|        |   |   +----------------69----------------+
|        |   |   |                                  \---- LM F09.0120(441)
|        |   |   |
|        |   |   +--------------------------------------- LM F09.0290(442)
|        |   |   |
|        |   |   +--------------------------------------- LM F11.0870(443)
|        |   |   |
|        |   |   +--------------------------------------- LM F13.0700(445)
|        |   |   |
|        |   |   +--------------------------------------- LM F14.0810(447)
|        |   |   |
|        |   |   |                                  /---- LM F14.1100(448)
|        |   |   |                             /-64-+
|        |   |   |                             |    \---- LM F14.1120(450)
|        |   |   +-------------100-------------+
|        |   |   |                             \--------- LM F14.1110(449)
|        |   |   |
|        |   |   |                                  /---- LM F16.1460(451)
|        |   |   +---------------100----------------+
|        |   |   |                                  \---- LM F16.1470(452)
|        |   |   |
|        |   |   |                                  /---- LM F16.1580(453)
|        |   |   +----------------99----------------+
|        |   |   |                                  \---- LM F35.2090(487)
|        |   |   |
|        |   |   +--------------------------------------- LM F17.0160(454)
|   /-54-+   |   |
|   |    |   |   |                                  /---- LM F17.0800(455)
|   |    |   |   +---------------100----------------+
|   |    |   |   |                                  \---- LM F32.0680(482)
|   |    |   |   |
|   |    |   |   +--------------------------------------- LM F17.1110(456)
|   |    |   |   |
|   |    |   |   +--------------------------------------- LM F18.1530(457)
|   |    |   |   |
|   |    |   |   +--------------------------------------- LM F18.1600(458)
|   |    |   |   |
|   |    |   |   |                                  /---- LM F19.0680(460)
|   |    |   |   |                             /100-+
|   |    |   |   |                             |    \---- LM F19.0690(461)
|   |    |   |   +-------------82--------------+
|   |    |   |   |                             \--------- LM F19.0700(462)
|   |    |   |   |
|   |    |   |   +--------------------------------------- LM F20.0640(463)
|   |    |   |   |
|   |    |   |   +--------------------------------------- LM F21.1040(464)
|   |    |   |   |
|   |    |   |   +--------------------------------------- LM F22.0560(465)
|   |    |   |   |
|   |    |   |   +--------------------------------------- LM F22.0960(466)
|   |    |   |   |
|   |    |   |   +--------------------------------------- LM F23.0560(467)
|   |    |   |   |
|   |    |   |   +--------------------------------------- LM F24.1430(469)
|   |    |   |   |
|   |    |   |   +--------------------------------------- LM F25.1950(470)
|   |    |   |   |
|   |    |   |   +--------------------------------------- LM F28.1850(472)
|   |    |   |   |
|   |    |   |   +--------------------------------------- LM F29.0970(473)
|   |    |   |   |
|   |    |   |   |                                  /---- LM F29.2380(474)
|   |    |   |   +---------------100----------------+
|   |    |   |   |                                  \---- LM F29.2390(475)
|   |    |   |   |
|   |    |   |   +--------------------------------------- LM F30.0350(476)
|   |    |   |   |
|   |    |   |   +--------------------------------------- LM F30.1450(477)
```

```
|   |    |   |   |
|   |    |   |   +-------------------------------------- LM F30.3060(478)
|   |    |   |   |
|   |    |   |   +-------------------------------------- LM F31.2710(480)
|   |    |   |   |
|   |    |   |   +-------------------------------------- LM F32.0420(481)
|   |    |   |   |
|   |    |   |   +-------------------------------------- LM F33.2140(483)
|   |    |   |   |
|   |    |   |   +-------------------------------------- LM F33.2560(484)
|   |    |   |   |
|   |    |   |   +-------------------------------------- LM F34.4260(486)
|   |    |   |   |
|   |    |   |   +-------------------------------------- LM F36.5150(489)
|   |    |   |   |
|   |    |   |   +-------------------------------------- CM O070C(491)
|   |    |   |   |
|   |    |   |   +-------------------------------------- CM Q429C(492)
|   |    |   |   |
|   |    |   |   +-------------------------------------- CM R497C(493)
|   |    |   |   |
|   |    |   |   +-------------------------------------- CM T097C(494)
|   |    |   |   |
|   |    |   |   |                                 /---- SC KAR3(496)
+57-+    |   |   |                            /-58-+
|   |    |   |   |                            |    \---- SP AC3A11.14C(521)
|   |    |   |   |                        /78-+
|   |    |   |   |                        |   \--------- SP AC664.10(522)
|   |    |   |   +----------73------------+
|   |    |   |   |                        \------------- PC GW.73.22.1(503)
|   |    |   |   |
|   |    |   |   |                                 /---- SC KIP2(498)
|   |    |   |   +---------------86----------------+
|   |    |   |   |                                 \---- SP BC1604.20C(524)
|   |    |   |   |
|   |    |   |   |                                 /---- SC KIP3(499)
|   |    |   |   |                            /-71-+
|   |    |   |   |                            |    \---- SP BC1685.15C(525)
|   |    |   |   |                        /64-+
|   |    |   |   |                        |   \--------- SP BC2F12.13(526)
|   |    |   |   +----------79------------+
|   |    |   |   |                        \------------- PC PC.95.48.1(508)
|   |    |   |   |
|   |    |   |   +-------------------------------------- SC SMY1(500)
|   |    |   |   |
|   |    |   |   +-------------------------------------- PC GW.4.121.1(501)
|   |    |   |   |
|   |    |   |   |                                 /---- PC GW.41.36.1(502)
|   |    |   |   +---------------55----------------+
|   |    |   |   |                                 \---- SP AC1834.07(519)
|   |    |   |   |
|   |    |   |   +-------------------------------------- PC PC.11.256.1(504)
|   |    |   |   |
|   |    |   |   +-------------------------------------- PC PC.3.10.1(506)
|   |    |   |   |
|   |    |   |   +-------------------------------------- PF MAL8P1.132(509)
|   |    |   |   |
|   |    |   |   +-------------------------------------- PF 07 0104(510)
|   |    |   |   |
|   |    |   |   +-------------------------------------- PF 11 0478(511)
|   |    |   |   |
|   |    |   |   +-------------------------------------- PF A0535C(512)
|   |    |   |   |
|   |    |   |   +-------------------------------------- PF C0860W(514)
|   |    |   |   |
|   |    |   |   +-------------------------------------- PF L0545W(515)
|   |    |   |   |
|   |    |   |   +-------------------------------------- PF L2190C(517)
|   |    |   |   |
|   |    |   |   +-------------------------------------- SP AC144.14(518)
|   |    |   |   |
|   |    |   |   +-------------------------------------- SP BC15D4.01C(523)
|   |    |   |   |
|   |    |   |   +-------------------------------------- TP 152375(528)
|   |    |   |   |
|   |    |   |   \-------------------------------------- TP 128511(529)
|   |    |   |
|   |    |   |                                     /---- AT 3G54870(37)
|   |    |   |                                /-71-+
|   |    |   |                                |    \---- PT LG VIII000260(423)
|   |    |   \--------------74----------------+
```

```
|   |    |                                     |    /---- OS SBCC009381(305)
|   |    |                                     \100-+
|   |    |                                          \---- OS IBCD008900(346)
|   |    |
|   |    |                                          /---- OS SBCC020273(319)
|   |    \-------------------100--------------------+
|   |                                               \---- OS IBCD019322(362)
|   |
|   |                                               /---- AT 1G12430(3)
|   \----------------------84-----------------------+
|                                                   \---- PT LG III0949(386)
|
\-------------------------------------------------------- PT LG II000635(430)
```
